# Synthetic, multi-dynamic hydrogels by uniting stress-stiffening and supramolecular polymers

**DOI:** 10.1126/sciadv.adr3209

**Published:** 2024-11-20

**Authors:** Laura Rijns, Martin G. T. A. Rutten, Riccardo Bellan, Hongbo Yuan, Mauro L. Mugnai, Susana Rocha, Emanuela del Gado, Paul H. J. Kouwer, Patricia Y. W. Dankers

**Affiliations:** ^1^ Institute for Complex Molecular Systems, Eindhoven University of Technology, P.O. Box 513, 5600 MB Eindhoven, Netherlands.; ^2^ Department of Biomedical Engineering, Laboratory of Chemical Biology, Eindhoven University of Technology, P.O. Box 513, 5600 MB Eindhoven, Netherlands.; ^3^ Molecular Imaging and Photonics, Department of Chemistry, KU Leuven, P.O. Box 2404, B3001 Leuven, Flanders, Belgium.; ^4^ Key Laboratory of Molecular Biophysics of Hebei Province, Institute of Biophysics, School of Health Sciences and Biomedical Engineering, Hebei University of Technology, Tianjin 300401, P.R. China.; ^*5*^ Institute for Soft Matter Synthesis and Metrology, Department of Physics, Georgetown University, Washington, D.C. 20057, USA.; ^6^ Radboud University, Institute for Molecules and Materials, P.O. Box 9010, 6525 AJ Nijmegen, Netherlands.; ^7^ Department of Chemical Engineering and Chemistry, Eindhoven University of Technology, P.O. Box 513, 5600 MB Eindhoven, Netherlands.

## Abstract

Nature uses discrete molecular building blocks to form polymers that assemble into multicomponent, multi-dynamic networks, inside (cytoskeleton) and outside (extracellular matrix) the cell. Both the intra-fibrous molecular dynamics and interactions between fibers dictate (non)linear mechanics, such as stress stiffening and relaxation, and ultimately biological function. Current synthetic systems capture only one dynamic process. Here, we present multi-dynamic hydrogels by uniting a stress-stiffening polymer with supramolecular polymers. Crucial is the molecular dynamics of the supramolecular polymers: They dictate the interaction strength with the stress-stiffening polymer and the subsequent dynamic mechanical properties of the mixed networks. The biological relevance of our multi-dynamic hydrogels is demonstrated by their ability to support fibroblast cell spreading. Future work may address the display of various dynamically presented bioactive cues to cells.

## Teaser

Multi-dynamic artificial hydrogels are created through uniting stress-stiffening and supramolecular polymers.

## INTRODUCTION

Single-component hydrogels are often used to mimic biological networks. Although they may contain multimode relaxation dynamics, they are not independently tunable (*1*–*8*). Examples of such dynamic processes include the fast molecular dynamics captured in peptide amphiphiles, stress-stiffening events in fibrin, or stress relaxation in collagen (*3*, *9*, *10*). Synthetic supramolecular polymers are emerging as a promising class of biomimetic hydrogels owing to their intrinsic dynamics arising at different length scales: (*11*) such as fast molecular dynamics at the fiber level due to monomer exchange from the supramolecular polymer fibers (*12*) and slow stress relaxation at the network level owing to fiber rearrangements (*11*, *13*). Excitingly, two-component networks are investigated to create more complex, life-like materials with emergent functions, allowing for the capture of multiple—often competing—desired properties within a single hydrogel, as pioneered by Gong *et al.* (*14*–*16*).

**Fig. 1.**
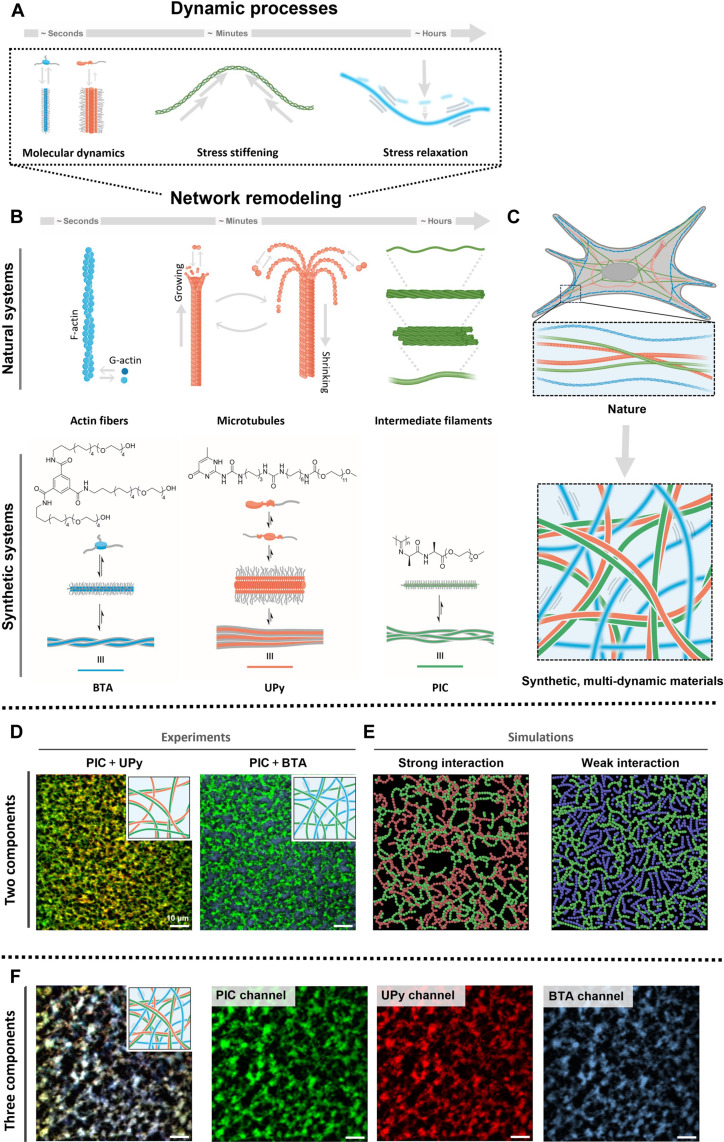
Fully synthetic, multi-dynamic hydrogel networks. (**A**) Biological relevant timescales in terms of mechanics and dynamics. (**B**) Timescales of important natural polymers and their corresponding synthetic counterparts, including the molecular design of the discrete monomeric building blocks used here. Left: BTA supramolecular monomers that form double helical fibers. Middle: UPy supramolecular monomers that form bundled fibers. Right: covalent PIC polymer that bundles into a soft network upon entropic desolvation. (**C**) The aim of this work is a fully synthetic hydrogel with control over various timescales, leading to nonlinear mechanics and eventually control over cellular function. (**D**) Fluorescence microscopy images of the colocalized PIC + UPy network and the interpenetrating PIC + BTA network, with PIC in green, UPy in red, and BTA in blue. Scale bar, 10 μm. (**E**) Model showing the structural organization of PIC + UPy having a strong interaction and of PIC + BTA having a weaker interaction. In green PIC, in red UPy, and in blue BTA. (**F**) Structural characterization of the three-component network consisting of the PIC, UPy, and BTA polymers, with PIC (green), UPy (red), BTA (blue). Scale bar, 10 μm.

Looking at nature, the dynamics within natural, biological polymers and the dynamic interactions between these different biological polymers in and outside the cell are tightly regulated to achieve biological function. Different dynamic processes can be distinguished, including molecular dynamics, such as monomer exchange, stress relaxation, induced by fiber and network remodeling, and stress stiffening owing to fiber stretching (Fig. 1, A and C). These different fibers interact (*17*–*19*). Intracellularly, dynamic microtubule fibers are protected by more ductile actin and intermediate fibers to accommodate higher compressing forces (Fig. 1B) (*18*). Extracellularly, in the native extracellular matrix, soft and dynamic hyaluronic acid reinforces and generates stress on rigid collagen fibers, leading to an increased linear stiffness and stress resistance (σ_max_, the maximum stress before network rupture) (*18*, *20*–*22*). The interaction between dynamic, flexible polymers with more robust networks yields unique (non)linear mechanics, which cover a wide range of timescales (*10*, *18*, *23*, *24*). Tight regulation of dynamic processes within and between the biological polymers is vital in nature, as any discrepancy can lead to dysfunction, like Alzheimer’s disease (*25*, *26*).

We found a multi-dynamic, fully artificial hydrogel by uniting a stress-stiffening polymer with supramolecular polymers displaying different dynamic behavior (Fig. 1, B and C). The supramolecular polymers exhibit different dynamics at both the molecular level (monomer exchange in fiber) as well as the bulk level (stress relaxation in hydrogel, Fig. 1B). Crucial is the molecular dynamics of the supramolecular polymers as they dictate the interaction strength with the stress-stiffening polymer and with that the (nonlinear) mechanical, structural, and dynamic properties of the hybrid hydrogel networks. Hence, using architecturally different networks that relax via completely different microstructural processes, we achieve more diverse function and independent tunability over dynamic and other properties. We found that tight control over all dynamic processes is required to induce cell adhesion.

## RESULTS AND DISCUSSION

As synthetic covalent stress-stiffening polymer (*27*), polyisocyanide (PIC) was combined with two types of supramolecular monomers forming either robust or more dynamic supramolecular polymers, respectively, to form two-component (Fig. 1, D and E) or three-component networks (Fig. 1F). Our ureido-pyrimidinone (UPy) monomers are able to form fibers with a monomer exchange of 10% in 1 hour and a stress-relaxing half-life time (τ_1/2_) > 1000 s (*12*, *28*, *29*), and the benzene-tricarboxamide (BTA) monomers are capable of forming double-helical fibers (with a monomer exchange of 35% in 1 hour and a τ_1/2_ ~ 200 s) (Fig. 1B) (*12*, *30*, *31*). Visualization of the networks after incorporation of fluorescent dyes on the networks (figs. S16 and S17) shows that PIC + UPy formed into an overlapping network (Pearson correlation coefficient, *r* = 0.77), while PIC + BTA formed a phase-separated network (*r* = −0.51), with BTA residing in the pores of PIC (Fig. 1D and figs. S28 to S30). The pores of PIC + UPy (2.0 μm) were almost twice as large as the pores of PIC + BTA (1.2 μm) (fig. S30), revealing large structural differences between the networks. The experimental results were supported by coarse-grained (CG) molecular dynamics simulations (Fig. 1E and figs. S24 and S26). In nature, controlled colocalization and phase separation constantly occurs, both intra- and extracellularly. For example, actin and filamin (an actin-binding protein) interact through a mechanism that involves both colocalization and phase separation (*32*). Colocation might be desired to achieve synergistic function: a state in which the properties of the hybrid network are greater than the sum of the individual components. On the contrary, phase separation with the formation of distinct domains in the cell might be desired to facilitate cell compartmentalization to regulate different processes.

**Fig. 2.**
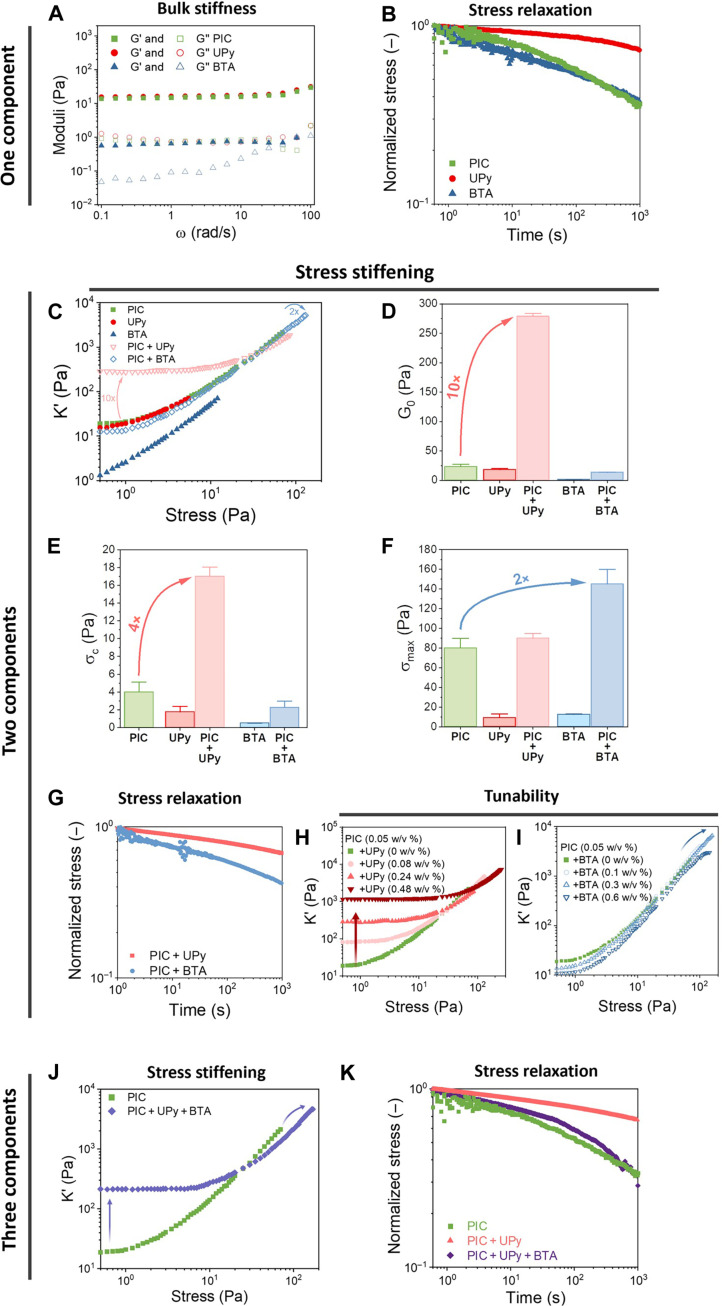
Synergistic properties in two- and three-component networks. (**A** and **B**) Mechanical and dynamic characterization of the one-component networks with (A) the bulk stiffness and (B) stress relaxation. (**C** to **I**) Mechanical and dynamic characterization of two-component networks. (C) The stress-stiffening response of the one- and two-component networks of PIC + UPy and PIC + BTA. (D to F) Quantification of stress-stiffening parameters of two-component networks with (D) the plateau modulus (G_0_), (E) the critical stress (σ_c_; onset of stress-stiffening), and (F) the stress resistance (σ_max_). (G) Dynamic quantification in terms of stress relaxation of two-component networks. (H and I) Tunable stress-stiffening response of (H) PIC + UPy showing tunable plateau modulus and of (I) PIC + BTA with tunable maximum stress. (**J**) Stress-stiffening and (**K**) stress relaxation behavior of the three-component network.

The mechanical properties of the two- and three-component networks were investigated with rheology using a prestress protocol that extracts the linear shear modulus *G*′ and the nonlinear stress-stiffening behavior, marked by the onset for stiffening (σ_c_) and the strength of the stiffening response in the stiffening index (*m*). While the single PIC and UPy networks formed relatively soft networks with a similar shear modulus of 20 Pa (Fig. 2A and fig. S13), the combination of the two networks increased *G*′ to 250 Pa (~10-fold increase; figs. S27 and S31). In addition, σ_c_ shifted to higher stress and *m* decreased toward 1 (Fig. 2, C to E, and fig. S21). We hypothesize that the increased σ_c_ and decreased *m* are caused by the higher network density with more steric crowding, in line with results from a synthetic bis-urea polymer gel (*33*) and aggrecan gels (*34*) that showed a decrease of *m* to 0.6 at increased concentrations. This higher network density might yield an increased bending rigidity owing to steric reinforcement (i.e., first bending of fibers and later followed by stretching of fibers), previously also observed in actin-vimentin networks (*35*).

By varying the UPy concentration (*c* = 0.08 to 0.48 w/v %), the enhancement in the shear modulus of the two-component gel could be tuned over an order of magnitude (*G*′ = 100 to 1000 Pa) (Fig. 2H and fig. S32). In contrast, PIC + BTA networks showed no increased *G*′ but did reveal synergistic effects in terms of maximum network strength before rupture (σ_max_); the two-component gel could be subjected to almost twice as much stress before rupture and reached a differential shear modulus *K*′ of ~500 times its initial value (Fig. 2, C and F, and figs. S21 and S31). This result suggests the presence of an additional mechanism, where BTA fibers are possibly able to dissipate energy around the PIC bundles upon increasing stresses (“unwinding” of BTA double helix), acting as a sacrificial component. In nature, microtubule fibers are known to act as a sacrificial component because its high bending stiffness suppresses subsequent bending of the surrounding actin fibers (*36*). Our enhanced stress resistance is again tunable via concentration changes of BTA (*c* = 0.1 to 0.6 w/v %) (Fig. 2I and fig. S32), allowing σ_max_ to vary between 80 and 160 Pa. At too high concentrations (*c* > 0.3 w/v %), however, BTA percolated and hindered PIC network formation, which compromises the stability of the two-component network at high stresses.

At low concentrations in dilute state (*c* ~ μM), UPy formed straight, parallel, and micrometer-long bundled fibers and the BTA shorter, more flexible double-helical fibers (fig. S12). The two-component mixtures in dilute state followed mostly the morphology of the long supramolecular fibers for the PIC + UPy network and of the smaller fibers for the PIC + BTA network (fig. S19). However, UPy and BTA seemed to interact differently with PIC; the robust UPy fibers slightly decreased PIC’s helicity (−7 to −6 mdeg) as measured with circular dichroism (CD) spectroscopy, while BTA slightly enhanced PIC’s helical structure (−7 to −9 mdeg) (fig. S22). Furthermore, super-resolution microscopy using stochastic optical reconstruction microscopy (STORM) showed that PIC formed around the robust UPy fibers, while the PIC coassembled with the more dynamic BTA fibers (fig. S20), further suggesting that the UPy versus BTA supramolecular fibers interacted differently with the PIC.

With regard to the dynamic properties of the networks, we previously found that for the single-component networks, BTA assemblies exhibit faster molecular dynamics than UPy (*28*, *37*), and formed a more dynamic network than UPy on gel level in bulk (Fig. 2B and fig. S14). Locally on 50-μm-length scales, fluorescence recovery after photobleaching (FRAP) experiments showed 30% FRAP for BTA versus 10% FRAP for UPy (fig. S15). The dynamic behavior of the single-component networks are translated into the two-component systems, where PIC + BTA forms a more dynamic network at both local length scales measured with FRAP (figs. S33 and S34) and in bulk measured with stress relaxation (τ_1/2_ = 400 s for PIC + BTA versus τ_1/2_ > 1000 s for PIC + UPy) (Fig. 2G and fig. S35).

Inspired by cross-talk between polymers in nature, for instance, between actin, microtubules, and intermediate filaments (*18*) we studied how we can expand the emerging mechanical behavior in three-component PIC + UPy + BTA gels, incorporating various timescales, i.e., molecular dynamics, stress stiffening, and stress relaxation (fiber/network remodeling). The three-component gels formed a highly colocalized network with almost complete overlap of the three components (Fig. 1F and fig. S48). Unexpectedly, the PIC + UPy + BTA network combined the various synergistic properties found before: an increased linear stiffness (*G*′ = 200 Pa, caused by UPy) and an extended stiffening regime due to the increased maximum stress (σ_max_ = 170 Pa, caused by the BTA) (Fig. 2J and fig. S49). Furthermore, the PIC + UPy + BTA network has similar stress relaxation behavior as compared to the PIC gel (τ_1/2_ ~ 100 s; Fig. 2K). These unique features only originate from the combination of these specific polymers; replacement of PIC with natural collagen (fig. S38) or removal of PIC (figs. S45 to S47) yielded networks with properties that are the mere average of the components lacking any synergistic effect.

**Fig. 3.**
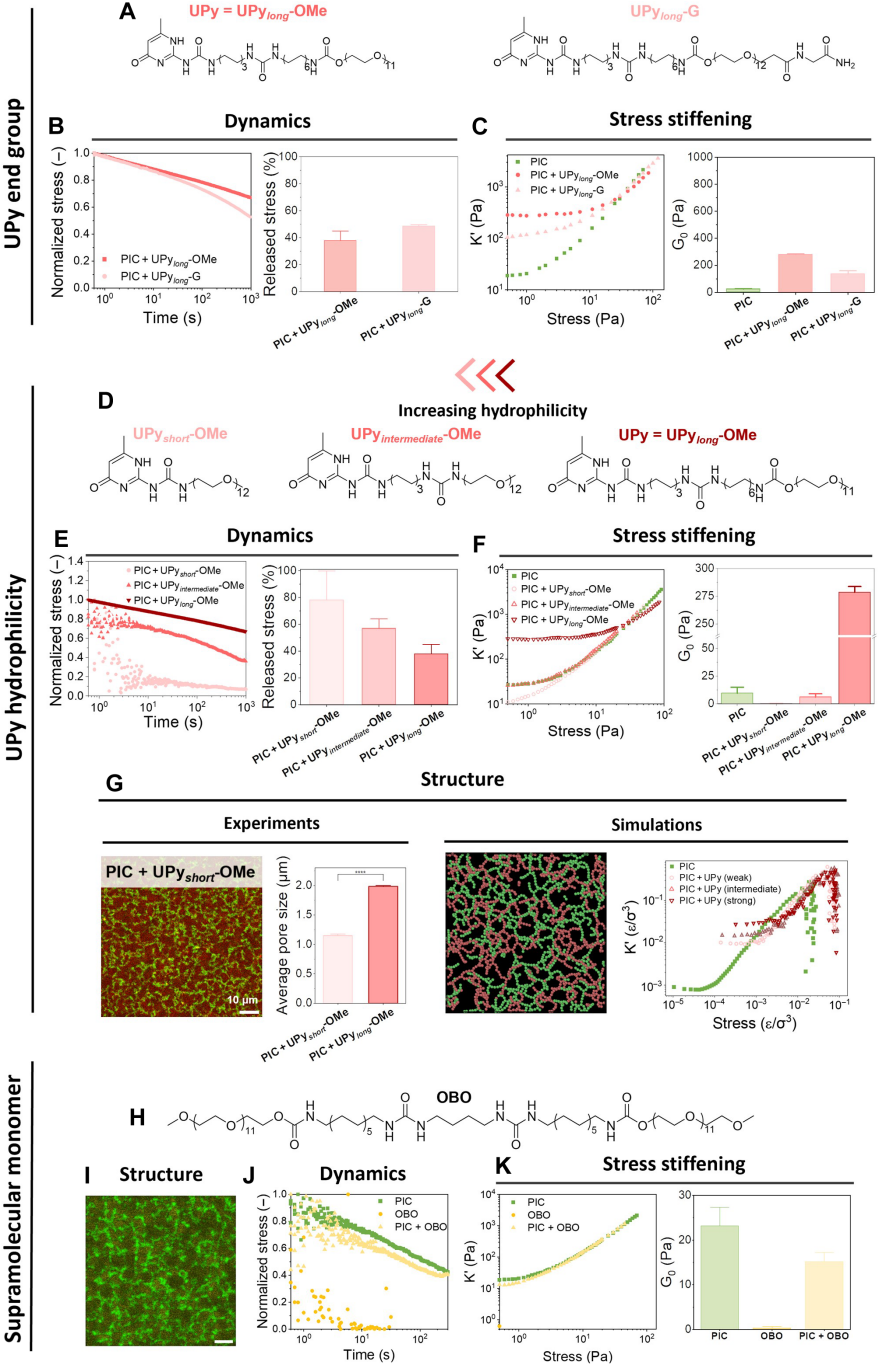
Synergistic properties are dictated by the molecular dynamics of supramolecular fibers. (**A** to **C**) The effect of UPy-end group (methoxy versus glycine) on stress-stiffening and bulk stiffness, with (A) chemical structures of UPy and UPy_long_-G and (B) a representative stress relaxation plot (left) and the released stress quantified after 1000 s (right), both exhibiting similar stress relaxation. (C) The stress-stiffening response (left) and the quantified plateau modulus G_0_ (right). (**D** to **G**) The effect of increased UPy hydrophilicity and with that increased molecular dynamics, on stress-stiffening and G_0_. (D) Chemical structures of the used UPy molecules with increased hydrophilicity by removing moieties as the urea group and hydrophobic spacers. (E) Representative stress relaxation plot (left) and the relaxed stress quantified after 1000 s (right) showing increased UPy hydrophilicity yielded increased dynamics. (F) Stress-stiffening response (left) and plateau modulus (right) of PIC combined with the various UPy’s. (G) Confocal image of PIC + UPy_short_-OMe and quantification of the pore size (left) and simulations of a PIC + UPy network with a weak PIC-UPy interaction, showing no overlapping network as well as marginal enhancement in the linear modulus (right). Scale bar, 10 μm. Data is plotted as mean with SEM. ****P < 0.0001, by Mann-Whitney. (**H** to **K**) Usage of bis-urea supramolecular building block as control for combination of PIC with supramolecular fibers. (H) Chemical structure of OBO. (I) Confocal image of the PIC (green) and OBO (yellow), showing no overlap. Scale bar, 10 μm. (J) Stress relaxation of PIC, OBO, and the two-component network. (K) Stress stiffening of PIC + OBO and the separate components (left) showing no enhanced properties in terms of plateau modulus (right).

To investigate the origin of the observed synergistic effects, we first investigated how molecular changes at the UPy fiber periphery (influence of UPy end group) affect the properties of the gels. Hence, we compared UPy (with a methoxy end group) to another supramolecular UPy monomer with a glycine end group (UPy-G; Fig. 3A and fig. S18), both forming robust, long fibers with, importantly, similar stress relaxation characteristics (Fig. 3B). Rheology showed that both the UPy and UPy_long_-G undergo a strong interaction with the PIC as for both gels, an enhanced *G*′ was measured (Fig. 3C and fig. S36). These results indicate that the UPy end groups (methoxy-methoxy interactions) are not controlling the interaction with PIC.

Next, we study the role of molecular dynamics on the interaction between PIC and UPy. We prepared two additional UPy molecules with decreasing length (UPy_short_ and UPy_intermediate_) (Fig. 3D). The decreased hydrophobicity of these molecules induces faster relaxation (reduced τ_1/2_) in the PIC gels (Fig. 3E). In line with these results, the more dynamic UPy shows more phase-separated networks with PIC (Fig. 3G, left), indicative of reduced interactions. Consequently, they show no enhancement in the linear stiffness (*G*′ ~ 10 Pa; Fig. 3F and fig. S37). Only the UPy molecule with a long hydrophobic spacer and additional urea moieties forms robust fibers (fig. S12) that are able to strongly interact with PIC and induce an increased linear stiffness (*G*′ = 250 Pa; Fig. 3F). Supplementary CG simulations based on a weaker PIC-UPy interaction strength also revealed the formation of a more phase-separated network (Fig. 3G, right, and fig. S26), lacking synergy with no increased bulk stiffness (Fig. 3G and fig. S25). Together, these data suggest that the molecular dynamics of the supramolecular fiber is the driving force for the interaction strength with PIC and the resulting synergistic linear stiffness increase. This conclusion was further underlined by using an even more dynamic supramolecular fiber, bis-urea (OBO) (τ_1/2_ < 1 s; Fig. 3, H and J), where mixing with PIC resulted in a phase-separated network (Fig. 3I) that lacked synergistic features with no increased bulk stiffness (Fig. 3K and figs. S40 and S53). Following both experimental and CG simulation data, we postulate that in case of fast molecular dynamics (fast monomer exchange), the hetero-fiber interactions are too weak (between PIC and dynamic BTA fibers) to achieve colocalization. On the contrary, in case of slow molecular dynamics (slow monomer exchange), the hetero-fiber interactions are strong enough (between PIC and robust UPy fibers), yielding an interacting, colocalized network with increased linear stiffness.

**Fig. 4.**
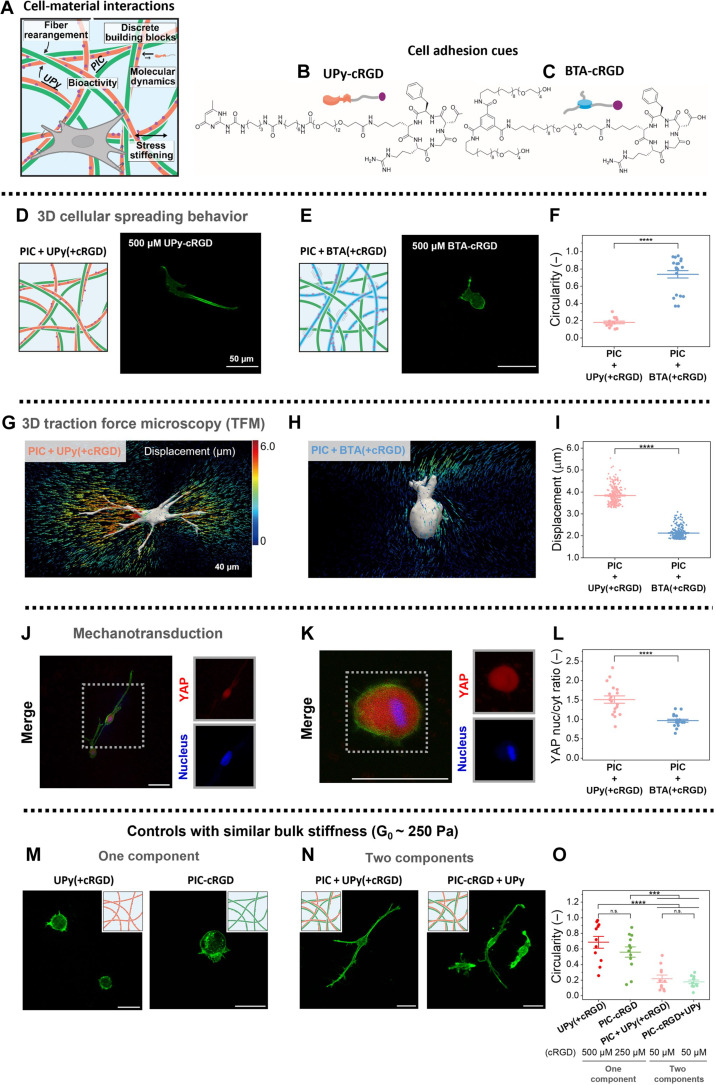
Cellular response in two-component networks dictated by control over timescales. (**A**) Two-component networks used as extracellular matrix mimics for cell culture. (**B** and **C**) Chemical structure of bioactive, cell-adhesive supramolecular monomers, with (B) UPy-cRGD and (C) BTA-cRGD. (**D** and **E**) Confocal fluorescent pictures (green = phalloidin = F-actin) of human normal dermal fibroblasts (hNDFs) cultured in 3D (D) PIC + UPy and (E) PIC + BTA and (**F**) quantification of cell circularity. Data points represent individual cells, plotted with means and SEM. ****P < 0.0001 by Mann-Whitney test. (**G** and **H**) 3D TFM experiments showing that cells can actively pull on the (G) PIC + UPy network and not on the (H) PIC + BTA network. (**I**) Quantification of the highest 5% bead displacements. Each data point represents a measured displacement, plotted with means and SEM. ****P < 0.0001 by Mann-Whitney test. (**J** to **L**) Investigation of mechanotransduction through YAP stainings, with (J) YAP confocal fluorescence images of PIC + UPy and of (K) PIC + BTA. Scale bar, 50 μm. (L) The quantified YAP nuclear/cytoplasmic ratio. Data points represent individual cells, plotted with means and SEM. ****P < 0.0001, by unpaired *t* test. (**M** to **O**) Cell culture in control hydrogels containing a similar bulk stiffness of ~250 Pa as PIC + UPy. Scale bar, 50 μm. (M) One-component network controls with UPy (left) and PIC (right) and (N) two-component networks with PIC + UPy(+cRGD) (left) and PIC-cRGD + UPy (right). (O) Quantified cell circularity of the control hydrogels. Data points represent individual cells, plotted with means and SEM. ***P < 0.001; ****P < 0.0001, by one-way analysis of variance (ANOVA) followed by Tukey post hoc. n.s., not significant.

To illustrate the biological relevance of the various timescales in our life-like materials, we investigated their interaction with cells (Fig. 4A). Both mechanical and dynamic properties are crucially important in living tissue. Here, we probe both effects simultaneously in our synthetic life-like materials by studying three-dimensional (3D) fibroblast growth. We hypothesize that in matrices with incompatible (nonlinear) mechanical properties or incompatible dynamic timescales, cells will stay spherical, while cells seeded in a suitable environment will have activated mechanotransduction pathways, leading to cell sprouting.

For these experiments, the cell-adhesion motif cRGD was introduced into the UPy supramolecular polymer, as UPy-cRGD monomer, and into and the BTA supramolecular polymer, as BTA-cRGD monomer, resulting in hydrogels in with the cRGD on either the UPy or BTA fibers, respectively (Fig. 4, B and C). Cells adhered and formed long elongated morphologies in the PIC + UPy(+cRGD) gel, in large contrast to the round cells found in the more dynamic and softer PIC + BTA(+cRGD) gel (Fig. 4, D to F, and figs. S42 and S43). 3D traction force microscopy (TFM) experiments showed that the spread cells in PIC + UPy(+cRGD) were able to adhere, stretch, and deform the network (average displacement, 3.9 μm), while the round cells in PIC + BTA(+cRGD) displaced the network much less (2 μm; Fig. 4, G to I). Together, these results indicate that cell spreading requires the incorporation of cell adhesion moieties in a robust fiber to generate traction, revealing the essential role of timescales in life-like materials as they control cell fate. This was also illustrated by the absence of cell spreading in the even more dynamic PIC + OBO(+cRGD) networks (fig. S41).

Putting these results into perspective and comparing BTA dynamics (especially the molecular dynamics, being ~seconds) (*12*) with that of clutch binding (~1 s) and subsequent focal adhesion (FA) lifetimes, which varies between ~10 to 100 s but increases toward minutes upon robust binding (*38*), it becomes clear that cell spreading is impaired in the BTA networks as their dynamic processes happen faster than clutch binding and FA lifetime. Therefore, material dynamics should match integrin dynamics to achieve biological function.

The large morphological differences of the cells suggest that the underlying mechanotransduction pathways were affected differently in the PIC + UPy(+cRGD) and PIC + BTA(+cRGD) networks. The mechanotransduction marker Yes-associated protein (YAP) was stained, and its nuclear/cytoplasmic translocation was quantified (Fig. 4, J and K) (*39*). A higher nuclear YAP translocation of ~1.5 was observed in the robust PIC + UPy(+cRGD) networks and higher levels of cytoplasmic YAP, with a nuclear/cytoplasmic ratio of ~1, was observed in the PIC + BTA(+cRGD) network (Fig. 4L). The YAP results imply that the difference in traction forces that cells can exert on the networks directly affect the downstream mechanotransduction pathways and the subsequent morphological differences.

We emphasize the importance of multicomponent networks with cell-matching time and force scales as cells cultured in single-component networks containing only UPy [i.e., UPy(+cRGD)] or only PIC (i.e., PIC-cRGD] with a similar G′ = 250 Pa remained round (Fig. 4, M and O, figs. S39 and S44). This directly suggests that, although the one-component versus two-component networks contain a similar bulk stiffness of 250 Pa, the stress stiffening of the PIC + UPy network might result in gels with a higher effective stiffness (i.e., true stiffness of the network upon exerted traction forces by cells) reaching toward ~1000 Pa, further supporting cell spreading. The crucial role of robust UPy fibers to reinforce the PIC (and subsequent increased stiffness and cell spreading) was further highlighted by two two-component control networks: one where cRGD was incorporated on the UPy fibers and one with cRGD covalently conjugated to the PIC polymer, PIC + UPy(+cRGD) or PIC-cRGD + UPy respectively. Both two-component networks displayed a similar G_0_ of ~250 Pa as the regular PIC + UPy network and induced cellular spreading (Fig. 4, N and O, and fig. S44). Together, this shows that a truly hierarchical synergistic network is formed when PIC and UPy are mixed, in which bioactive signals can be transduced from the supramolecular to the PIC network (adhesion signals can be on either of the two polymers). This additionally emphasizes that cell adhesion motifs should be presented in their right timescale in the bulk network to cells.

**Fig. 5.**
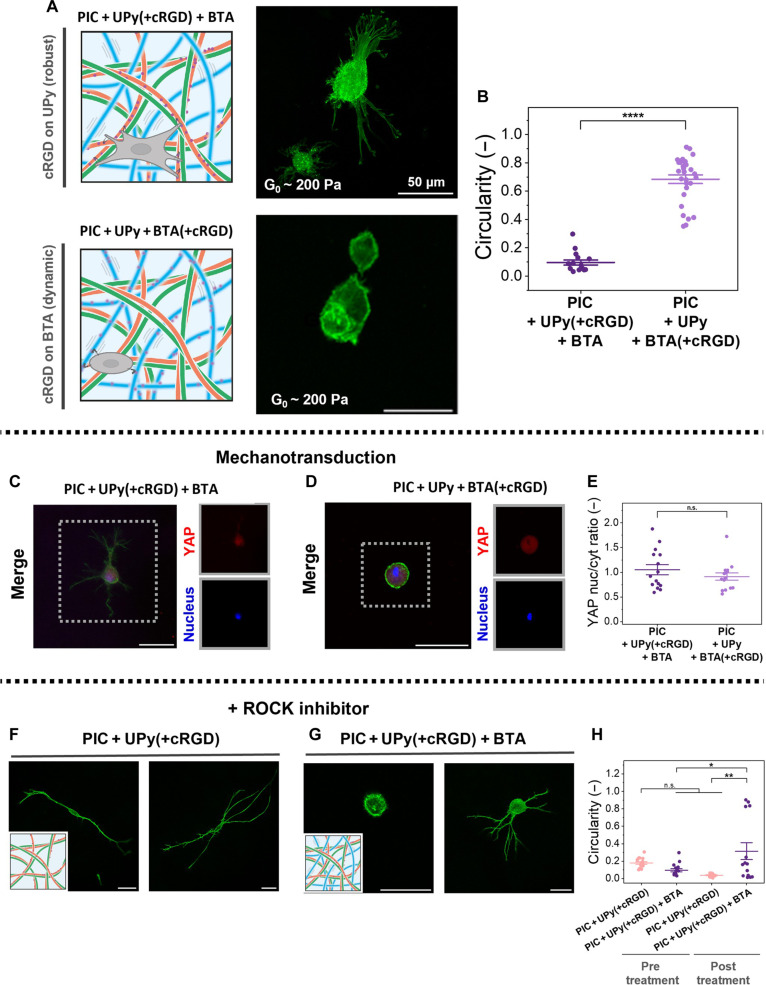
Cellular response in three-component networks—molecular dynamics dictate cellular spreading, independent of bulk stiffness, stress stiffening, stress relaxation, and ligand concentration. (**A**) Confocal fluorescence images (green = phalloidin = F-actin) of hNDFs cultured in 3D in the three-component networks containing similar bulk stiffness, stress stiffening, and stress relaxation. 0.5 mM cRGD is either incorporated within the (top) robust UPy fibers (UPy-cRGD) or in the (bottom) more dynamic BTA fibers (BTA-cRGD). Scale bar, 50 μm. (**B**) Quantification of cell circularity. Data points represent individual cells, plotted with means and SEM. ****P < 0.0001 by Mann-Whitney test. (**C** to **E**) Investigation of mechanotransduction through YAP stainings. Scale bar, 50 μm. (C) YAP confocal fluorescence images of PIC + UPy(+cRGD) + BTA and (D) PIC + UPy + BTA(+cRGD) and (E) the quantified YAP nuclear/cytoplasmic ratio. Data points represent individual cells, plotted with means and SEM. ****P < 0.0001, by unpaired *t* test. (**F** to **H**) Investigation of underlying mechanotransduction chain involved in cellular spreading in the two (elongated cells) versus three-component network (round cell body with many protrusions) through ROCK inhibitors. Scale bar, 50 μm. (F) ROCK added to the two-component PIC + UPy(+cRGD) and (G) to the three-component network PIC + UPy(+cRGD) + BTA. (H) Quantification of cell circularity of the two and three networks pre and post ROCK treatment. Data points represent individual cells, plotted with means and SEM. *P < 0.05; **P < 0.01, by one-way ANOVA followed by Tukey post hoc.

To conclusively prove the importance of the molecular dynamics, the cell-adhesion motif cRGD was included in the three-component network in two distinct ways: in the robust UPy fibers [PIC + UPy(+cRGD) + BTA or in the more dynamic BTA fibers [PIC + UPy + BTA(+cRGD)] (Fig. 5A). 3D cell experiments revealed that cells only spread in the gel with the cRGD included on the robust UPy fibers and not in gel with cRGD included on BTA (Fig. 5, A and B, and fig. S50). Thus, molecular dynamics dictate cellular spreading as the network density (and with that pore size; Fig. 1F), (non)linear stiffness (Fig. 2J), and stress relaxation (Fig. 2K) are similar in both networks. The cRGD moiety needs to be robustly incorporated in the supramolecular fibers to withstand cellular traction forces—which is only possible in the robust UPy fibers. Including cRGD in the BTA fibers reveals that the dynamic BTA fibers cannot withstand cellular traction forces. Further quantification of YAP translocation showed no significantly higher nuclear/cytoplasmic ratio in the network with UPy-cRGD than in the BTA-cRGD–based gel (Fig. 5, C to E), which implies that the mechanotransduction pathway to nucleus is not fully completed in the flexible three-component network as compared to the more rigid two-component PIC + UPy (Fig. 4, J and L).

Moreover, it did not escape our attention that the cells spread differently in the three-component network PIC + UPy(+cRGD) + BTA as compared to the double PIC + UPy(+cRGD) networks: The cells are more round with many protrusions in the three-component network (Fig. 5A), whereas cells could convey contractile forces to the network to build up tension and elongate in the two-component network (Fig. 4, D and G). Additional quantifications of several cell morphological features, including the area of the main cell body (1200 μm^2^ for two-component versus 600 μm^2^ for three-component network), the circularity of the main cell body (0.2 for two-component versus 0.7 for three-component network), and length of the longest axis of the main cell body (30-μm two-component versus 10-μm three-component network) clearly highlighted these differences (fig. S51). The origin for these morphological cell differences might be the increased stress relaxation of the three-component network as compared to the two-component system (70% recovery versus 30% recovery, τ_1/2_ 100 s versus >1000 s, respectively) (Fig. 2, G and K)—offering less resistance from material to cell in the three-component network, reducing the timescale in which cells can effectively stress their microenvironment. Another explanation includes the higher stress sensitivity of the three-component network compared to the two-component system, where stress stiffening started at lower stresses (10 instead of 17 Pa), which could cause cells to probe a different stiffening response (fig. S49). Alternatively, the higher network density (i.e., total fraction of solid material) in the three-component network as compared to the two-component network might hamper cell spreading. Building on this, we are currently exploring the composition space in the three-component system, tuning network stiffness and relaxation dynamics by altering UPy and BTA concentrations to favor cell spreading (*40*).

To further investigate the differences in the mechanotransduction pathway for the elongated cells (two-component) versus the star-shaped cells with many protrusions (three-component network with UPy-cRGD), Rho-kinase (ROCK) inhibitors were added to the cell cultures immediately at the start of the culture period. ROCK inhibitors are known to decrease intracellular contractile forces through decreasing phosphorylation of the myosin light chain (*39*, *41*). The ROCK inhibitor did not suppress cell spreading in the elongated cells in the two-component network (more YAP nuclear translocation) but did prevent spreading, to some extent, for the star-shaped cells in the three-component network (Fig. 5, F and H, and fig. S52). Apparently, the ROCK inhibitor could not prevent cell spreading when the YAP pathway was activated, whereas it could when the YAP pathway was not active and not involved cellular spreading. These data imply that YAP and ROCK are related; however, this relation reveals itself to be rather complex as many other cellular elements next to actin stress fibers (e.g., the linker of nucleoskeleton and cytoskeleton complex and force on nucleus) influence mechanotransduction (*39*, *42*, *43*). In addition, the relation between physical properties resulting in mechanotransduction is not linear, complex, and yet poorly understood, especially for viscoelastic materials (*44*).

To conclude, we engineered various biologically relevant dynamic processes into fully synthetic materials to recreate nature’s complexity, while other synthetic systems can only capture one dynamic process. Crucial here is the molecular dynamics of the supramolecular polymers: They dictate the interaction strength with the covalent polymer and with that the subsequent (nonlinear) mechanical, structural, and dynamic properties of the hybrid hydrogel networks. Cell growth experiments demonstrated the biological relevance of the various timescales that were engineered in our life-like materials: Cell spreading was controlled by the molecular dynamics, which dictates both bulk and nonlinear stiffness and the dynamic ligand presentation. Future work may address the targeted delivery of bioactive cues to cells with timescales that are optimized toward specific cellular responses.

## MATERIALS AND METHODS

### Materials

All solvents were of Analytic Research (AR) quality and purchased from Biosolve. Deuterated compounds were obtained from Cambridge Isotope Laboratories and stored over 4-Å molecular sieves. Dry solvents were obtained using MBraun solvent purification system (MB SPS-800). Water for aqueous samples was purified on an EMD Millipore Milli-Q Integral Water Purification System. Glassware was dried in an oven at 135°C overnight before reactions under dry conditions.

### Methods

#### Thin-layer chromatography

Reactions were followed by thin-layer chromatography using 60-F254 silica gel plates from Merck and visualized by ultraviolet (UV) light at 254 nm and/or staining (ninhydrin, potassium permanganate).

#### Flash column chromatography

Flash column chromatography was performed on a Grace Reveleris X2 chromatography system using Reveleris Silica Flash cartridges.

#### Nuclear magnetic resonance

All ^1^H nuclear magnetic resonance (NMR) and ^13^C-NMR spectra were recorded on Bruker Ultrashield spectrometers (400 MHz for ^1^H NMR and 100 MHz for ^13^C NMR). Proton chemical shifts are reported in parts per million (ppm) (δ) downfield from trimethyl silane (TMS) using the resonance frequency of the deuterated solvent (CDCl_3_; 7.26 ppm) as the internal standard. Peak multiplicities are abbreviated as s: singlet; d: doublet; dt; doublet of triplet; m: multiplet. Carbon chemical shifts are reported in parts per million (δ) downfield from TMS using the resonance frequency of the deuterated solvents (CDCl_3_; 77.16 ± 0.06 ppm) as the internal standard.

#### Fourier transform infrared

Infrared spectra were recorded using a PerkinElmer Spectrum Two Fourier transform infrared spectrometer equipped with a PerkinElmer Universal ATR Two Accessory. All solid-state spectra were measured at room temperature from 500 to 4000 cm^−1^ and were averaged more than 16 scans.

#### Liquid chromatography–mass spectroscopy

Liquid chromatography–mass spectroscopy spectra were acquired using a device consisting of multiple components: Shimadzu SCL-10 A VP system controller with Shimadzu LC-10 AD VP liquid chromatography pumps [with an Alltima C18 3 u (50 mm by 2.1 mm) reversed-phase column and gradients of water], a Shimadzu DGU 20A3 prominence degasser, a Thermo Finnigan surveyor auto sampler, a Thermo Finnigan surveyor photodiode array (PDA) detector, and a Thermo Fisher Scientific LCW Fleet. All samples were dissolved in 1:1 H_2_O:acetonitrile (ACN, v/v) in ~0.1 mg/ml concentration.

#### Sample preparation of hydrogels

(a) Plain hydrogels: The solid UPy, BTA, OBO powders were weighed separately into 1.5-ml clean, separate glass vials. Then, BTA and OBO were dissolved in adequate amount of Milli-Q (MQ) water. UPy was dissolved in adequate amount of basic MQ (80 mM NaOH). Parafilm was applied around the lid to prevent evaporation. The samples were heated for ~30 min (depending on the polymer’s concentration) at 70° or 80°C in a water bath. During and after heating, the samples were vortexed several times for 10 s. For the BTA samples, a stirring bar was included and the solution was stirred during the heating. After heating, the UPy solution was neutralized using 1 M HCl. For the PIC solution, solid PIC was dissolved in MQ at 4°C overnight while occasionally shaken and afterward kept on ice. Then, UPy, BTA, OBO solution were mixed immediately from the 80°C water bath (i.e., from “nearly” monomeric state) with the PIC solution that was still on ice (i.e., in liquid state).

(b) Hydrogels containing dyes for visualization: Separate hydrogels were prepared similar to the procedure stated above. Then, UPy-FITC (fluorescein isothiocyanate), UPy-Cy5 (Cyanine), UPy-Cy3, BTA-Cy5, or OBO-Cy5 was added from a stock of organic solvent [i.e., ACN, MeOH, or dimethyl sulfoxide (DMSO)] at 500 μM or 1 mM stock concentrations into the hydrogels to obtain the target concentration. The dye-containing sample was then heated for an additional 5 to 10 min at 40°C to ensure a homogeneous dye distribution throughout the hydrogel.

PIC-labeled gels were prepared by mixing azide functionalized PIC (PIC-N_3_) with DBCO-PEG_4_-TAMRA or DBCO-PEG_4_-FL-BDP (1 mM in MQ or DMSO respectively) at 4°C for 1 hour. The resulting dye-labeled hydrogels were then left to equilibrate overnight at 37°C in the oven, and measurements were executed the next day.

(c) Hydrogels containing cRGD-functionalized monomers for cell culture: Separate hydrogels were prepared similarly to the procedure stated above. The cRGD-functionalized monomers were included in the UPy, BTA, or OBO fibers. To realize this, the solid UPy-cRGD powder was weighed separately into a 1.5-ml clean, separate glass vial. UPy-cRGD was then dissolved in adequate amount of basic MQ water (80 mM NaOH). Parafilm was applied around the lid to prevent evaporation. The sample was heated for ~15 min at 70°C in a water bath. During and after heating, the samples were vortexed several times for 10 s. The UPy-cRGD was then pipetted into the nonfunctionalized UPy assemblies immediately from the hot solution and heated for an additional 5 min at 70°C to ensure homogeneous distribution. The UPy mixture was then neutralized with 1 M HCl. The BTA-cRGD and OBO-cRGD were weighed together as solids with the nonfunctionalized BTA or OBO, respectively. The solutions were heated for ~30 min at 80°C in a water bath while stirring. During and after heating, the samples were vortexed several times for 10 s. The cRGD-functionalized assemblies were then pipetted in the hot, “nearly molecular dissolved state” into the PIC solution that was held an ice. The resulting solution was pipetted up and down several times without generating bubbles to ensure homogeneous mixing. The final cRGD-containing solutions were kept on ice until they were mixed with cells (see Cell culture section).

(d) Three-component system: For the nonfunctionalized hydrogels, the hot UPy solution was pipetted into thePIC solution on ice, after which the hot BTA solution was pipetted into it. A similar order was followed for the dye- and cRGD-containing components.

(e) Diluted state: UPy, BTA, and OBO solid powders were weighed into clean, 1.5-ml glass vials. BTA and OBO were dissolved in adequate amount of MQ water to obtain the target concentration. UPy was dissolved in adequate amount of basic MQ (80 mM NaOH). The solutions were heated for ~30 min at 70° or 80°C in a water bath while stirring. During and after heating, the samples were vortexed several times for 10 s. If required, dye-containing monomers were added from a stock of organic solvent (i.e., ACN, MeOH, or DMSO) at 500 μM or 1 mM stock concentrations into the solutions to obtain the desired concentration. The dye-containing sample was then heated for an additional 5 to 10 min at 40°C to ensure a homogeneous dye distribution throughout the hydrogel. After heating, the UPy solution was neutralized using 1 M HCl. PIC or PIC-N_3_ was dissolved in MQ at 4°C overnight while occasionally shaken and afterward kept on ice. If required, DBCO-PEG_4_-TAMRA (1 mM in MQ) was mixed with PIC-N_3_ for 1 hour at 4°C. The UPy, BTA, or OBO solutions were then mixed from the “hot” state with PIC that was still on ice, after which the samples were equilibrated overnight at 37°C. The next day, the experiments were performed [i.e., UV, CD, STORM, total internal reflection microscopy (TIRF), and cryo–transmission electron microscopy (cryo-TEM)].

#### Rheology

Rheological measurements were carried out on an Anton Paar (Physica MCR 501), or a discovery hybrid rheometer (DHR-3 or HR-30, TA Instruments). Hydrogels were prepared according to the sample preparation method and scooped onto the rheometer equipped with cone plate geometry (diameter, 25 mm) at a gap height of 49 μm. All measurements were performed at least in duplo and measured at 37°C. A solvent trap was used to minimize sample drying during the measurements. Strain sweep experiments were measured at strains ranging from 1 to 100%, at a constant frequency of 1 rad/s. Frequency sweeps were performed at frequencies ranging from 100 to 0.1 rad/s, at a constant strain of 1%. Stress relaxation tests were performed under 7.5% strain with a strain rise time of 0.01 s. The released stress was quantified after 1000 s.

Hydrogel formation was followed on the DHR-3 or HR-30 rheometer using a 20-mm aluminum cone-plate (2.007°) geometry with a truncation gap of 56 μm or a 20-mm steel cone-plate (0.994167°) geometry with a truncation gap of 27 μm, respectively. After loading, the samples were immediately heated to 37°C and the complex modulus G* was measured by applying an oscillating deformation of amplitude γ = 1% at a frequency of 1 rad/s for 24 hours until a stable network was formed.

For nonlinear measurements, the prestress protocol described by Broedersz *et al.* (*45*) was used, i.e., the network was subjected to a constant prestress σ_0_ = 0.5 to 300 Pa, with a small oscillatory stress δσ superposed at a frequency of ω = 10 to 0.1 Hz. The differential modulus was calculated by *K*′ = δσ/δγ. The plateau shear modulus was estimated from the linear region of the stress-stiffening curve to describe the mechanical parameters of the material at low stress, i.e., before stress stiffening occurs. Practically, this means that the plateau modulus is the average of the first three data points from the stress-stiffening curve. This plateau modulus matches the values that are obtained in the linear regions of the frequency sweeps (0.1 to 10 rad/s) and strain sweeps (1 to 10%). As for the pristine BTA network, the stress-stiffening curve starts to increase immediately, and the first value of the stress-stiffening curve is used here as the plateau modulus as this value matches the linear region in the frequency sweep.

#### Fluorescence recovery after photobleaching

FRAP measurements were carried out using a Leica TCS SP5 or SP8 inverted confocal microscope (Leica Microsystems) equipped with a 20× objective (HCX PL APO CS 20 × 0.7 DRY UV). The samples, each containing 15 μM of dye (i.e., UPy-Cy5, UPy-FITC, BTA-Cy5, OBO-Cy5, and PIC-TAMRA) were prepared according to the preparation procedure for samples containing dyes and pipetted into an Ibidi micro-angiogenesis glass-bottom well plate. To minimize sample drying during the measurements, the empty wells were filled with water. Exchange dynamics experiments were carried out via sample illumination using white laser at λ = 488, 555, or 633 nm for FITC, TAMRA, or Cy5 excitation, respectively. Emission was collected at λ= 495 to 550, 565 to 625, and 645 to 735 nm, respectively, using a hybrid detector. A circular area with a diameter of 50 μm was photo-bleached at 100% laser power until a bleached spot was reached [ranging from 100 to 1000 frames (0.653 frame/s)], and the post-bleaching time-lapse imaging was performed for 6 hours when possible. To correct for drift of the hydrogel, the region of interest was adjusted manually if necessary. Image analysis was performed in Leica AF software and in ImageJ. Data processing was performed in Excel and Origin. Data were normalized by dividing the fluorescence intensity by the maximum observed fluorescence intensity in a non-bleached circular area of the same size for each measurement point. The % recovery of the bleached spot after 6 hours was averaged for each condition over at least two independent experiments. Confocal images of 1024 pixels by 1024 pixels were created before (100-Hz scan speed) during and after photobleaching (100-Hz scan speed).

#### Confocal fluorescence images

Hydrogels at with 15 μM dye were prepared according to the hydrogel formulation method containing dyes (method b in the section “Sample preparation of hydrogels”). Mixed hydrogel solutions were pipetted into an Ibidi micro-angiogenesis glass-bottom well plate. To minimize sample drying during the measurements, the empty wells were filled with water. Images were acquired using a Leica TCS SP8 inverted confocal microscope (Leica Microsystems) equipped with a 63× objective (HC PL APO CS2 63 × 1.20 water). Samples were illuminated using λ = 488, 555, or 633 nm for BDP-FL/FITC, TAMRA, or Cy5 excitation, respectively. Emission was collected at λ = 495 to 550, 565 to 625, and 645 to 735 nm, respectively, using a hybrid detector or a photomultiplier tube. Confocal images were taken using 4096 by 4096 pixels using a scan speed of 1 Hz or 1096 pixels by 1096 pixels using a scan speed of 10 Hz. Each hydrogel was prepared in duplo and at least seven random positions were chosen to make images. All measurements were performed at room temperature. ImageJ was used to calculate the Pearson correlation coefficient between channels. To calculate pore sizes, a previous developed Matlab script was used (*46*). In short, images were segmented using an adaptive threshold to minimize differences in illumination or local background. After segmentation, a binary image was generated where each pixel is assigned to either a pore or fiber. The detected pores are divided into sections, which were used to calculate various parameters as average, medium, and maximum pore size. Pearson correlation coefficient was calculated using ImageJ.

#### Cell culture

Human normal dermal fibroblasts (hNDFs) were cultured in Advanced Dulbecco’s modified Eagle’s medium (Advanced DMEM; Gibco) supplemented with 10 v/v % fetal bovine serum, 1 v/v % (penicillin-streptomycin), and 1 v/v % Gluta MAX at 37°C and 5% CO_2_. The medium was changed every 5 days, and cells were passaged using trypsin/EDTA at 90% confluency. The cells were cultured from the 12th passage until passage number 19. For 2D cell culture, UV-sterilized hydrogels containing 1 mM cRGD-functionalized monomers were prepared in Ibidi micro-angiogenesis slides 1 day before cell seeding and allowed to equilibrate overnight at 37°C. The next day, 1 × 10^3^ hNDFs were seeded in a U-shaped 96-well plate on top of the hydrogels and cultured for 1 day at 37°C with 5% CO_2_.

For 3D cell seeding, 1 × 10^3^ hNDFs were seeded per hydrogel. Experiments with higher cell densities were performed with 5 × 10^4^ hNDFs seeded per hydrogel. For the three-component system with the cRGD moiety on the robust UPy fibers, the components were mixed in the following v/v % ratio: 17/25/33/25 = PIC/UPy-cRGD/BTA/cells. For the three-component system with the cRGD moiety present on the more dynamic BTA fibers, the components were mixed in the following v/v % ratio: 25/25/25/25 = PIC/UPy/BTA-cRGD/cells. For the three-component cell culture, 1 × 10^3^ hNDFs per hydrogel were seeded. Gels with cells encapsulated inside were left for gelation for 30 min to 1 hour at 37°C before the medium was added in a very careful, dropwise manner.

#### Immunofluorescence staining and confocal microscopy

After 1 day culture at 37°C with 5% CO_2_, the samples were washed with phosphate-buffered saline (PBS), followed by fixation for 10 min with 3.7 v/v % formaldehyde in PBS and washing with PBS twice afterward. Cell samples were then permeabilized and blocked using PBS containing 10 v/v % donkey serum and 0.2 v/v % Triton X-100 at room temperature for 3 hours. Samples were then washed twice with PBS. Cell samples were then incubated with YAP primary antibody (clone EP1674Y form, Abcam, 1:50) in PBS containing 0.2 v/v % Triton X-100 in the fridge overnight. Samples were washed with 0.2 v/v % Triton X-100 in PBS three times. Alexa Fluor 647 donkey anti-rabbit secondary antibody was then added in 0.2 v/v % Triton X-100 in PBS to the cells (1:200) for 2 hours. Samples were washed with 0.2 v/v % Triton X-100 in PBS three times. Afterward, the samples were stained with phalloidin to visualize F-actin for 1 hour (1:400) or with 4′,6-diamidino-2-phenylindole (DAPI) to visualize cell nuclei for 5 min (1:200) in PBS with 0.2 v/v % Triton X-100 in PBS at room temperature in the dark. Last, the samples were washed with PBS containing 0.2 v/v % Triton X-100. Immediately thereafter, the samples were imaged (and only mounted if necessary) on a Leica TCS SP5 inverted confocal microscope using 10× (HCX PL APO CS 10.0 × 0.4 DRY UV), 20× (HCX PL APO CS 20.0 × 0.7 DRY UV), and 40× (HCX PL APO CS 40.0 × 1.1 water UV) objectives. Image analysis and quantification of cell area and circularity (*n* is at least 10 cells per condition) was performed using LAS X and Fiji (ImageJ) software. Additional experimental details for YAP nuclear translocation can be found in section 1 of the Supplementary Materials.

#### 3D displacement microscopy

For cell traction–induced 3D matrix displacement measurements (3D displacement microscopy), hNDFs were stained with CellTracker Green (Life Technologies) for 40 min before encapsulation for image visualization and analysis. Fluorescent beads that serve as fiducial markers [FluoSpheres, 0.2 μm, red fluorescent (excitation/emission = 660/680 nm), Thermo Fisher Scientific, F8807], were embedded in the cell-gel constructs (1:667 dilution). The beads were thoroughly vortexed before addition and were mixed such that they were distributed randomly and homogeneously throughout the hydrogels, while the individual beads could also be distinguished from each other. The displacement field was quantified by tracking the positions of the embedded beads before and after the cell tractions were relaxed with cytochalasin D (cytoD, 5 μM). Ten microliters of the cell-gel-beads mixture was pipetted in a μ-Slide Angiogenesis dish (Ibidi) on ice and then warmed up in an incubator to create cell-gel constructs.

##### Image acquisition

Fluorescence images were acquired using a Leica SP8 confocal microscope with a 25× water-immersion objective [numerical aperture (NA), 0.95] and a hybrid photomultiplier tube as detector (HYD-SMD, Leica). Cells labeled with CellTracker Green were excited at 492 nm, and fluorescent beads were excited at 646 nm with a nonsequential and bidirectional mode. The image stack was recorded typically with 175 *z* slices and 512 pixels × 512 pixels in the *x*,*y* plane. Voxel dimensions were 0.57 μm horizontally in the *x* and *y* directions and also 0.57 μm vertically in the *z* direction. A series of time-lapse image stacks were recorded after adding cytoD, with an interval time of 20 min between each *z* stack. A stage incubator was used to keep the cells at 37°C and 5% CO_2_ during image acquisition.

##### Displacement calculation

A Matlab-based open source software package, TFMLAB, was used to process the images and calculate matrix displacement field, developed by Van Oosterwyck group (*47*, *48*). Briefly, TFMLAB integrates several computational steps to analyze the data, including image processing, cell segmentation, image alignment, and matrix displacement measurement. This software is freely available at https://gitlab.kuleuven.be/MAtrix/Jorge/tfmlab_public.

#### Immunofluorescence staining and confocal microscopy

After 1 day of cell culture, the samples were washed with PBS, followed by fixation for 10 min with 3.7 v/v % formaldehyde in PBS and washing with PBS twice afterward. Cell samples were then permeabilized and blocked using PBS containing 10 v/v % donkey serum and 0.2 v/v % Triton X-100 at room temperature for 3 hours. The samples were then washed twice with PBS. Cell samples were then incubated with YAP primary antibody (clone EP1674Y form, Abcam, 1:50) in PBS containing 0.2 v/v % Triton X-100 in the fridge overnight. The samples were washed with 0.2 v/v % Triton X-100 in PBS three times. Alexa Fluor 647 donkey anti-rabbit secondary antibody was then added in 0.2 v/v % Triton X-100 in PBS to the cells (1:200) for 2 hours. The samples were washed with 0.2 v/v % Triton X-100 in PBS three times. Afterward, the samples were stained with phalloidin to visualize F-actin for 1 hour (1:400) or with DAPI to visualize cell nuclei for 5 min (1:200) in PBS with 0.2 v/v % Triton X-100 in PBS at room temperature in the dark. Last, the samples were washed with PBS containing 0.2 v/v % Triton X-100. For imaging, the samples were scooped out of the U-shaped 96-well plate with a small spoon and placed on top of a glass, microscope plate. A round-shaped coverslip was placed on top (10-mm diameter), and the sample was squeezed a bit between the two slides to allow for sharper images. Immediately thereafter, the samples were imaged (and only mounted if necessary to prevent drying out) on a Leica TCS SP5 inverted confocal microscope using 10× (HCX PL APO CS 10.0 × 0.4 DRY UV), 20× (HCX PL APO CS 20.0 × 0.7 DRY UV), and 40× (HCX PL APO CS 40.0 × 1.1 water UV) objectives.

#### Pharmacological treatment with ROCK inhibitors to investigate mechanotransduction

ROCK inhibitor Y-27632 (gifted by W. de Lau, Hubrecht Institute) was added to the initial cell suspension mixed with the hydrogel at 0 hours (1:1000 dilution) as well as to the supplemented growth medium after 1 hour of gelation (1:1000).

#### UV-visible absorbance spectra

UV-visible spectra were recorded on a JASCO J-815 CD Spectrometer equipped with a Peltier temperature controller. A scanning rate of 50 nm/min, a bandwidth for monitoring of 2.0 nm, a response time of 1 s, a data pitch of 1 nm, two-component accumulation, and a scanning range of 190 to 450 nm were used. Samples were prepared in 1-mm Quartz cuvettes with a concentration of four times lower than used for plain hydrogels (sample preparation method a), i.e., 0.125 mg/ml for PIC, 0.6 mg/ml for UPy, and 0.75 mg/ml for BTA, allowed to form a gel and equilibrate overnight at 37°C and measured at 37°C. A baseline of the corresponding solvent (MQ water with adjusted pH) was measured and automatically baseline corrected from all spectra.

#### Circular dichroism

CD and linear dichroism spectra were measured on a JASCO J-815 CD Spectrometer equipped with a Peltier temperature controller. A scanning rate of 50 nm/min, a bandwidth for monitoring of 2.0 nm, a response time of 1 s, a data pitch of 1 nm, two-component accumulation, and a scanning range of 190 to 450 nm were used. Samples were prepared in 1-mm Quartz cuvettes with a concentration of 0.5 and 0.125 mg/ml for PIC, 2.4 and 0.6 mg/ml for UPy, and 3 and 0.75 mg/ml for BTA; allowed to form a gel and equilibrate overnight at 37°C; and measured at 37°C. A baseline of the corresponding solvent (MQ water with adjusted pH) was measured and automatically baseline corrected from all spectra.

#### Cryo–transmission electron microscopy

Cryo-TEM images were created by R.B. using samples of five times–lower concentration than used for plain hydrogels, with final concentration 480 μM. Vitrified films were prepared in a “Vitrobot” instrument (PC controlled vitrification robot, patent applied 2002, patent licensed to FEI) at 22°C and at a relative humidity of 100%. In the preparation chamber of the Vitrobot, a 3 μl of the sample was applied on a Quantifoil grid (R 2/2, Quantifoil Micro Tools GmbH), which was surface plasma treated just before use (Cressington 208 carbon coater operating at 5 mA for 40 s). Excess sample was removed by blotting using filter paper for 3 s at −3 mm, and the thin film thus formed was plunged (acceleration about 3 g) into liquid ethane just above its freezing point. Vitrified films were transferred into the vacuum of a CryoTITAN equipped with a field emission gun that was operated at 300 kV, a post-column Gatan energy filter, and a 2048 by 2048 Gatan charge-coupled device (CCD) camera. Virtrified films were observed in the CryoTITAN microscope at temperatures below −170°C. Micrographs were taken at low-dose conditions, starting at a magnification of 6500 with a defocus setting of 40 μm, and at a magnification of 24.000 with a defocus setting of 10 μm.

#### TIRF and STORM images

Dye-containing samples were prepared according to the sample preparation procedure in diluted state. STORM and TIRF images were acquired with a Nikon N-STORM system. Dyes were excited using a 561- and 647-nm laser, respectively. Fluorescence was collected by a Nikon 100×, 1.4-NA oil immersion objective and passed through a quad-band pass dichroic filter (97335, Nikon). Images were recorded with an electron multiplying CCD camera (ixon3, Andor, pixel size 0.17 μm). The movies were subsequently analyzed with the STORM module of the NIS element Nikon software. After equilibration overnight, the samples were diluted to enable the visualization of individual dyes. The sample was flown in a chamber between a clean, Pyranha-etched glass microscope coverslip (Menzel-Gläser, no. 1, 21 mm by 26 mm) and a glass slide, which were separated by two-component–sided tape. After annealing for several minutes, the chamber was washed twice with imaging buffer containing 50 mM tris-HCl (pH 7), an oxygen scavenging system [glucose oxidase (0.5 mg/ml) and catalase (40 μg/ml)], 10 w/v % glucose, and 10 mM 2-aminoethanethiol. For TIRF imaging, assemblies were prepared as stated above, and after equilibration for at least 1 day, the samples were diluted and thereafter flown in a chamber between a glass microscope coverslip and a glass slide, without additional washing with buffer.

#### CG simulations

All the simulations were performed using a version of LAMMPS (*49*) (Apr. 2014) that was modified by us to include the energy function used in the gel model.

##### Energy function

The energy function for the gel model and the protocol used to generate the network have been described in numerous publications (*50*, *51*). For details, we refer to the literature; however, here, we recapitulate the meaning of relevant parameters and procedures to highlight the features of the implementation adopted for this study. The model described in previous publications will be referred to as “M0.”

All parameters discussed are either dimensionless or are given in microscopic units such as *d*, and ϵ for length and energy. *d* corresponds to the linear size of an elementary building block of the gels, in our CG description, and ϵ corresponds to the typical interaction strength that drives their self-assembly and keep the gels together. The building blocks of the gels are considered to be embedded in a solvent that exerts on them a drag ζ (as a result of the solvent viscosity and the building block shape). In all rheological data, we use as microscopic time unit the timescale over which the gel building blocks, experiencing a drag ζ and being attached to a gel with a force ϵ/*d*, move of a distance comparable to their linear size *d*. In the same spirit, the shear stress has units of ϵ/*d*^3^. Using *d* ≈ 1 μm and ϵ ≈100 *k*_B_*T*, where *T* ≈ 300 K, leads to ϵ/*d*^3^ ≈ 0.4 Pa and τ ≈ 2 · 10^−2^ s.

The gel is composed of colloidal-size units (we refer to them as particles) that interact via a combination of two-body (E_2_) and three-body (E_3_) potentials

$$E\left({\vec{r}}_{1},\ldots ,{\vec{r}}_{N}\right)=\sum_{i=1}^{N-1}\sum_{j=i+1}^{N}E_{2}\left(r_{\mathrm{ij}}\right)+\sum_{i=1}^{N-1}\sum_{j\neq i}\sum_{k>j\&k\neq i}E_{3}\left({\vec{r}}_{\mathrm{ij}},{\vec{r}}_{\mathrm{ik}}\right)$$ (1)

where ${\vec{r}}_{\text{ij}}$ = ${\vec{r}}_{j}$ − ${\vec{r}}_{i}$, *r_ij_* = ∣${\vec{r}}_{\text{ij}}$∣ is the magnitude of the vector distance between particles *i* and *j*. The first set of summations goes over all *i*-*j* pairs, with *j* > *i* so that they are only selected ones; the second set of summations includes any “central” particle i and two different “surrounding” particles *j* and *k* with *k* > *j* so that no triplet is considered twice. The two-body and three-body potentials are

$$E_{2}\left(r_{\mathrm{ij}}\right)=A\epsilon \left[{\left(\frac{d_{2}}{r_{\mathrm{ij}}}\right)}^{18}-{\left(\frac{d_{2}}{r_{\mathrm{ij}}}\right)}^{16}\right]H\left(r_{\mathrm{cut}}-r\right)$$ (2)

$$\begin{array}{c} E_{3}\left({\vec{r}}_{\mathrm{ij}},{\vec{r}}_{\mathrm{ik}}\right)=B\epsilon \Lambda \left(r_{\mathrm{ij}}\right)\Lambda \left(r_{\mathrm{ik}}\right)e^{{-[\frac{\text{cos}\left(\theta_{\mathrm{ijk}}\right)-\text{cos}\left(\bar{\theta }\right)}{\omega }]}^{2}}\\ \Lambda \left(r\right)={\left(\frac{d_{3}}{r_{\mathrm{ij}}}\right)}^{10}{\left[1-{\left(\frac{r}{r_{\mathrm{cut}}}\right)}^{10}\right]}^{2}H\left(r_{\mathrm{cut}}-r\right) \end{array}$$ (3)

The two-body term (Eq. 2) features a strong repulsive core for *r* < *r*_min_ = (9/8)^1/2^*d*_2_ ≈ 1.06*d*_2_, and a short attraction tail that is set to zero for *r* > *r*_cut_ as shown with the step function *H*(*r*_cut_ − *r*) (= 1 if *r* ≤ *r*_cut_ and = 0 otherwise). The location of its minimum represents the diameter of the colloidal beads. In the M0 version of the model, *d*_2_ = 0.922*d*, so that *r*_min_ ≈ 0.98*d*, and thus the colloidal particles have diameter ≈ *d*. At *r* = *r*_min_, *U*_2_(*r*_min_) ≈ −0.043*A*ϵ; thus, *A* regulates the depth of the potential in internal energy units. In the M0 version of the model, *A* = 23, leading to *E*_2_(*r*_min_) ≈ −ϵ. The cutoff of interactions is set at *r*_cut_ = 2*d*, where the potential, in the absence of the cutoff, would be equal to ≈ −3*A*ϵ10^−6^, which is small for all the values of *A*.

The three-body term (Eq. 3) is exclusively repulsive and, combined with the two-body one, determines the favorable bond angles in a triplet of colloidal particles of which the ith is assumed to be central, and the jth and kth are connected to the ith through the vectors ${\vec{r}}_{\text{ij}}$ and ${\vec{r}}_{\text{ij}}$, respectively. The angle θ*_ijk_* is obtained from the scalar product between ${\vec{r}}_{\text{ij}}$ and ${\vec{r}}_{\text{ij}}$, cos(θ*_ijk_*) = ${\vec{r}}_{\text{ij}}$ ·${\vec{r}}_{\text{ij}}$/(*r_ij_r_ik_*). The repulsive force arising from this term is strong if the cosine of this angle is within w from cos(θ) and decays exponentially outside of this range. In the M0 model, θ ≈ 65°, or cos(θ) = 0.4226, and *w* = 0.3. The distance range over which the potential operate is tuned by Λ(*r*), which is = 0 for *r* ≥ *r*_cut_, and the parameter *d*_3_ = 1.1*d*. The strength of the repulsion is regulated by *B*, which is assigned the value of 10 in the M0 model. The energy profile for three particles interacting with each other when two are at their energy minimum and the third is used as a probe to compute the energy field surrounding the other two (fig. S23), where panel (A) and (B) differ because in (B), the three-body term is set to zero.

##### Preparation of the gels

Gels are prepared via a procedure consisting of three steps. First, the position of colloidal particles are placed on a lattice and randomized by running high-temperature (*T*05/*k_B_*) simulations via a Nosé-Hoover (NH) thermostat implemented in LAMMPS. Next, we quench and then relax the system at a lower temperature, *T*005/*k_B_* using, again, NH thermostat to mimic the coupling of the system to a thermal bath. Last, we drain the kinetic energy from the system by running simulations at zero temperature and in the presence of viscous damping. At the end of this process, the temperature has dropped to values below 10^−10^/*k_B_*, and the structure reveals a local minimum of the energy landscape or inherent structure of the gel. This model of an athermal gel corresponds to physical systems in which scale of interaction energies bonding the colloidal units of the network is ϵ ≫ *k_B_T_r_*, where *T_r_* = 300 K is room temperature.

##### Frequency Spectrum and linear response

The tailoring of model parameters was predominantly made to recover the experimentally observed trends in the elastic moduli for different gels. We determined the elastic (G′) and loss (G″) moduli using the optimally windowed chirp protocol (*52*), which was recently shown to yield accurate and fast results for the viscoelastic spectra over five orders of magnitude in frequency (*53*). The details of the method are discussed by Bouzid *et al.* (*53*), but, briefly, we conducted zero-temperature simulations of a gel under Lees-Edwards periodic boundary conditions (*54*) and subject to oscillatory deformations. The frequency of oscillation is increased exponentially in time, and the time-dependent amplitude γ(*t*) features a maximum of γ_0_ = 0.01, within the linear regime for these gels (*50*) and is enclosed in a tapered and symmetric window between *t* = 0 and *t* = *T*, the total simulation time (*53*). We monitor the stress of the system as a function of time defined by the virial equation $\sigma_{\alpha \beta }\left(t\right)=-V^{-1}\sum_{i=1}^{N}r_{i,a}\left(t\right)F_{i,\beta }\left(t\right)$, where *V* is the system volume and the Greek letters refer to Cartesian coordinates (this is a simplified description that does not account for periodic boundary conditions; for details on the calculation of the stress tensor, we refer to the LAMMPS manual and the work by Thompson *et al.* (*55*). Stress and strain are then Fourier transformed, and the elastic and loss moduli are respectively equal to the real and imaginary parts of the ratio of σ˜(ω)/γ˜(ω), where the symbol *x˜*(ω) refers to the Fourier transform of *x*(*t*). We show the plots up to the frequency ω*_c_* at which G′(ω*_c_*) = G″(ω*_c_*). As already discussed in previous work (*53*), at higher frequencies, there is a resonance due to inertia of the colloidal units of the gel. These frequencies are not of our interest here as they are not accessed in experiments.

##### Non-linear response

Start-up shear: Experimental measurements reported the stress dependence of the differential modulus, *K* = (∂σ/∂γ), and illustrated elastic regime, stiffening of the gel, and final fracture at high stress. To compare with experiments, we performed start-up shear simulations. The detailed protocol is published elsewhere (*50*) but, briefly, we subject the system to a strain step ∆γ by deforming the box while using Lees-Edwards periodic boundary conditions, and then we allow the gel to relax for a time ∆*t* according to a zero-temperature dissipative dynamics. The effective strain rate ∆γ/∆*t* is chosen to be smaller than γ_0_ω*_c_*. We selected ∆γ = 0.01, and thus ∆γ/∆*t* < γ_0_ω*_c_* implies that ∆*t* > ∆γ/(γ_0_ω*_c_*) = 1/ω*_c_*. The smallest ω*_c_* for PIC, UPy, PIC + BTA, and PIC + UPy was ω*_c_* ≈ 1/(134τ); therefore, we chose ∆*t* = 10^3^τ and used the same for all systems. During step and subsequent relaxation, we monitored the shear stress and we computed the differential modulus using the following numerical derivative. Just before the (*n* + 1)th strain step, the total strain is *n*∆γ and the total simulation time is (*n* + 1)∆*t*, as the first n steps are performed at zero strain, and the post-relaxation stress is σ*_n_* = σ[(*n* + 1)∆*t*, *n*∆γ]. We approximated the corresponding differential modulus as *K*(σ*_n_*) ≈ {σ[(*n* + 2)∆*t*, (*n* + 1)∆γ] − σ[*n*∆*t*, (*n* − 1)∆γ]}/(2∆γ) = (σ_*n*+1_ − σ_*n*−1_)/(2∆γ), that is we take the difference of the stress at the end of the next step and of the previous one and we divided them by twice the strain-step size. The curves showing the differential modulus as a function of stress are in fig. S25.

##### Porosity

We used two methods to compute the porosity of the network. In the first approach, we adopted an algorithm outlined in the Introduction of the work by Bhattacharya and Gubbins (*56*) without the fast refinement proposed by the authors in the rest of the manuscript. Here, we describe the steps in detail. We take a conformation of the gel in a periodic box of size *L* and volume *V* = *L*^3^, and we assign to each colloidal particle a radius *r*_colloid_ = 0.5*d*. Next, we construct a grid of *N*^3^ points ${\vec{r}}_{\text{grid}}$ (*i*, *j*, *k*) = *L*/*N_g_*(*i*, *j*, *k*), where *i*, *j*, *k* ∈ [0, *N_g_* − 1], and we assign to each vertex of this lattice the value *R*_grid_(*i*, *j*, *k*) corresponding to the smallest distance between that point and all of the colloidal particles, that is, *R*_grid_(*i*, *j*, *k*) = max ({min_*l*=1,⋯*N*_ [∣${\vec{r}}_{l}$ − ${\vec{r}}_{\text{grid}}$(*i*, *j*, *k*)∣ − *r*_colloid_],0)}, where index *l* runs over the colloidal particles. Here, we have also subtracted from each distance the radius of the colloidal particle, and if the distance is less than *r*_colloid_, we count it as zero. In plain words, the quantity *R*_grid_(*i*, *j*, *k*) represents the largest sphere that we can fit at that grid point without clashing with the gel.

Next, we place a probe particle in a random location, ${\vec{r}}_{\text{probe}}$, within the simulation box. We run over the list of lattice points, and we compute *R*_probe_ = max_*i*,*j*,*k*_({*R*_grid_(*i*, *j*, *k*) where ∣${\vec{r}}_{\text{probe}}$ − ${\vec{r}}_{\text{grid}}$(*i*, *j*, *k*)∣ < *R*_grid_(*i*, *j*, *k*)}), that is, we consider all of the lattice points such that the probe particle is positioned within *R*_grid_(*i*, *j*, *k*) from a (*i*, *j*, *k*) lattice site, and among those we keep the largest *R*_grid_(*i*, *j*, *k*). We reject the sampled point either if it overlaps with a colloidal particle or if it cannot be assigned to any grid point, which can happen given the finite spacing between grid points. If the sampled point is rejected, we ignore it and try again with a new randomly placed probe.

Last, we repeated this calculation *N_r_* times and construct the probability *P*(*r* > *R*), that is the probability that if a new probe is randomly placed in the interstitial space between the gel branches, it will be found in a spherical pore of size *r* > *R*. Analogously, we can write $P\left(r>R\right)=\int_{R}^{\infty }p\left(r\right)\mathrm{dr}$, where *p*(*R*)*dR* = −*dP*(*r* > *R*)/*dRdR* is the probability distribution of finding the newly inserted probe in a pore of size between *R* and *R* + *dR*. It follows that we can define an average pore size as $\left\langle R\right\rangle =\int_{0}^{\infty }\mathrm{Rp}\left(R\right)\mathrm{dR}=-\int_{0}^{\infty }\mathrm{RdP}\left(r>R\right)/\text{dRdR}=\int_{0}^{\infty }P\left(r>R\right)\mathrm{dR}$, which is the area under the probability *P* (*r* > *R*).

In most cases we have used *N_r_* = 5 · 10^4^ and *N_g_* = 25. For a few cases we repeated the calculation 3 times to test whether the results are reproducible, and we found that the deviation between different samples are small. In one case, we repeated the calculation using changing *N_g_* and *N_r_*. The discrepancies are small in all cases, but it looks like *N_g_* ≥ 15 is sufficient to get consistent results, and that *N_r_* = 10^5^ does not affect the outcome.

We adopted also a second method to compute the pore size. The procedure is the same as the one just outlined until the construction of the grid. After that, there are two differences from the previous algorithm: (i) Instead of sampling random points, we select grid nodes; (ii) we do not count the number of times a pore is identified but only whether it is identified and whether it is larger than a cutoff *r_c_* = 0.25*d* to avoid very small niches that might not be experimentally detectable and could be a consequence due to the spherical shape of the colloidal particles. The difference between the two approaches is that the former gives a weight to each pore proportional to the amount of volume assigned to that pore, whereas the second one averages over all the pores found and is in closer agreement with the measurements in the experiments.

##### Model parameters for PIC, BTA, UPy, and two-component networks

We tuned the parameters of the M0 gel model to reproduce, at least at a qualitative level, (i) the relative plateau of the elastic moduli for all of the networks and (ii) the morphology of the networks. We decided to adjust only three parameters: (i) the strength of the two-body potential, *A*; (ii) the strength of the three-body potential, *B*; and (iii) the angle cos(θ). After some testing, we decided to keep the volume fraction of each network to ϕ = 0.05. Extracting this quantity from the weight percentage used in experiments is not trivial, given that our colloidal particles and their volume exclusion represent aggregates of many individual units of each chemical species, and it is not clear how many units of a given species constitute one of our beads because they could have different packing and, hence, various densities.

In the following, we describe in detail how we have chosen the parameters used in this study (see Table 1). We remark here that other set of parameters could have produced similar agreement with experiments.

**Table 1.** Model parameters. Rows 2 to 4 refer to parameters for the intraspecies interactions, whereas interspecies interaction parameters are in the fifth and sixth rows. The last column refers to the order with which the gels were prepared.

Species	A	B	cos(θ)	Order
PIC	30	10	0.4226	N/A
UPy	55	10	0.34071	N/A
BTA	1.833	10	0.4226	N/A
PIC-UPy	1	0	N/A	Simultaneous
PIC-BTA	1.833	10	0.4226	Sequential

PIC: We use the M0 gel model for PIC with only a small modification to the parameters: The strength of the two-body interaction, A, was raised from 23 to 30 with the goal of increasing the frequency, ω*_c_*, at which G′ and G″ cross. This choice facilitated the start-up strain simulations by reducing the time needed to relax the network between two consecutive discrete strain steps.

UPy: Because UPy forms stiffer fibers than PIC, we decided to parametrize UPy by first reducing the value of cos(θ) for interspecies interactions. This creates fibers that have higher persistence length and that are less branched than those of the M0 model, as it was shown in previous studies that had created fibrous gels (*51*, *57*). After a range of tests to identify the conditions that best match the experimental systems, we have chosen cos(θ) = 0.34071 and performed a series of simulations in which we progressively refined only the strength of the UPy-UPy interaction (A) to reproduce the experimental observation that the G′ plateaus of PIC and UPy nearly overlap. This whole process was conducted in parallel with the refinement of the strength of the interaction between PIC and UPy.

BTA: The parameters for BTA were inspired by a previous study in which variants of the M0 model were adopted to provide microscopic insights to the structure and rheology of a mixture of two components: BTA and a bifunctional BTA-PEG-BTA (*58*). As pure components at 5 w/v %, BTA formed a self-assembled network, whereas BTA-PEG-BTA formed micelles (*58*). The current study features BTA at 0.3 w/v %, resulting in a plateau modulus around G′(ω = 0.1 rad/s) ≈ 1 Pa, which is about the same as BTA-PEG-BTA and nearly 10 times smaller than BTA in the previous study. For these reasons, in this study, we used the BTA-PEG-BTA parameters of (*58*) to model BTA.

PIC + UPy: To create the two-component PIC + UPy network, we started with PIC particles on a lattice, randomized them at high temperature, and then inserted UPy beads in random locations and proceed by slowly quenching the two gels together. Confocal microscopy images showed that PIC and UPy colocalize. We used this information to set the interspecies strength of the three-body interaction, *B* = 0 for triplets of interacting colloidal units involving both PIC and UPy. Previous simulations had shown that such a choice of interspecies three-body interaction strength could yield interpenetrating polymer network (IPN) morphology displaying colocalized strands of different components wrapped around each other. This can be understood by comparing the energy experienced by a probe particle placed in the vicinity of two other colloidal beads in contact with each other. Figure S23 (A and B) displays the cases for *B* = 10 and *B* = 0, respectively. The disappearance of the repulsive lobes around *y *= 0 favors conformations in which different network branches are side-to-side or twisted around each other. What is left to parametrize is the strength of the two-body PIC-UPy interaction. We speculate that the interaction strength should be weaker than the intraspecies ones and yet large enough to lead to the colocalization observed in experiments. We tried to optimize this together with the UPy-UPy interaction energy, obtaining a ratio between the PIC-UPy and the PIC plateaus of the elastic moduli to be around 30, whereas experimentally the enhancement is of about 12.5. We deem this an acceptable agreement.

PIC + BTA: Confocal microscopy images showed that PIC and BTA demix. Following previous experience with modifying the parameters in the M0 model (*51*), we set *B* = 10 and varied *A*. However, we found that even setting a small value of the strength of the two-body interaction between PIC and BTA, the network displayed a much larger plateau than pure PIC. This is likely a consequence of the fact that when the two networks form simultaneously, BTA confines PIC and this volume exclusion effect alters the morphology of the growing PIC network. This affected the linear response of the PIC-BTA network and thus would not recover the experimental observation that the plateau moduli of PIC and PIC + BTA are nearly the same. To solve this issue, we look at the kinetic of network formation study in experiments by monitoring the growth rate of the elastic moduli of PIC and BTA networks. PIC forms much more rapidly, and we rationalize that if our choice of ϕ = 0.05 for all species overestimates the volume fraction actually occupied by BTA, we could solve the issue by imposing that PIC networks form before BTA, in agreement with insights from experiments. We did this by creating a PIC network using the standard procedure and then anchoring each colloidal particle to its position within the gel using a harmonic tethering potential of stiffness 1.0ϵ/*d*^2^. Next, we inserted the BTA particles and we quenched them within the restrained PIC network. Last, we remove the tethering potential and we drained the kinetic energy from the two-component gel. The resulting structure was used for analysis.

#### Analysis of the simulation results

##### Frequency spectra

With the exception of BTA, which in our model is essentially fluid, changes in parameters have larger effects on G′ than on G″. In particular, the plateau and the crossing point between G′ and G″ extend to larger frequencies as the strength of the two-body interaction is increased.

##### Stiffening

Start-up shear simulations show that all systems with the exception of BTA are essentially linear up to strain γ = 0.1. Most formed gels show strain stiffening to various degrees at γ ≈ 0.3 to 0.4. However, increasing the strength of PIC-UPy interaction suppresses stiffening (fig. S25C), whereas UPy-UPy interaction seems to predominantly shift its onset (fig. S25D). Accordingly, the shape of the differential modulus as a function of stress seems to be nearly insensitive to the strength of UPy-UPy interaction (fig. S25D). In contrast, stiffening is nearly lost when PIC-UPy interaction strength is increased. To gain further insights, we plot the differential modulus and stress rescaled by ϵ*A*/*d*^3^, where *A* is the strength of the two-body interaction modified in simulation—either PIC-UPy or UPy-UPy. As shown in fig. S25 (E and F), in both cases, the data collapse on a master curve describing the differential modulus as a function of stress. However, while regardless of the strength of UPy-UPy interaction, all the curves show nearly the whole profile, when PIC-UPy interaction strength is modified, the domain of the master curve covered by each profile changes as well.

##### Average pore size

The average pore size as a function of the relative strength of interaction between PIC and UPy or UPy and UPy (fig. S26) was calculated from two different methods of computing and are in qualitative agreement. While the average pore size is insensitive to tuning the UPy-UPy interaction, increasing the strength of PIC-UPy contacts results in networks with larger pores. Experiments in which the length of the UPy tail were changed showed that the pore size of the network increased, and we used this observation to rationalize that the change in the length of UPy tails regulates heterotypic more than homotypic interactions. If we consider only PIC fibers to compute network porosity, we find that changes in the energy function have little effect on the average pore size. Tuning the UPy-UPy interaction strength results in a non-monotonic profile with minimum close to the parameter values obtained from the parameter refinement guided by the comparison with experiments. Instead, increasing PIC-UPy interactions opens slightly larger pores. Last, pores between UPy fibers alone are insensitive to changes in PIC-UPy interaction, whereas increasing UPy-UPy interaction strongly increases pore size up to a saturation or even inversion point. We rationalize our observations as follows: Increasing PIC-UPy interaction creates larger pores in the network mostly due to PIC wrapping tightly around UPy, whose fibers are instead only weakly affected by changes to PIC-UPy interaction. Changing UPy-UPy interaction leads to UPy separating from PIC and forming compact clusters with large pores between them. These observations are recapitulated in fig. S26, where we show images of slices of the self-assembled networks as a function of the strength of the PIC-UPy and UPy-UPy interactions.

## Supplementary Materials

### This PDF file includes:

Supplementary Text Figs. S1 to S53 References
