# Supplementary material for: Synthetic, multi-dynamic hydrogels by uniting stress-stiffening and supramolecular polymers
Source: Sci Adv. 2024 Nov 20;10(47):eadr3209. doi: 10.1126/sciadv.adr3209 (PMC13109944; doi:10.1126/sciadv.adr3209)
Supplement: Supplementary file 1 — Supplementary Text Figs. S1 to S53 References [file sciadv.adr3209_sm.pdf]

Science Advances
AAAS

Supplementary Materials for

Synthetic, multi-dynamic hydrogels by uniting stress-stiffening and supramolecular polymers

Laura Rijns *et al.*

Corresponding author: Patricia Y. W. Dankers, p.y.w.dankers@tue.nl

Sci. Adv. **10**, eadr3209 (2024)
DOI: 10.1126/sciadv.adr3209

This PDF file includes:

Supplementary Text
Figs. S1 to S53
References

1. Additional Materials and Methods

Immunofluorescence staining and confocal microscopy: after 1 day culture at 37 °C with 5% CO_2, the samples were washed with PBS, followed by fixation for 10 min with 3.7 v/v% formaldehyde in PBS and washing with PBS twice afterwards. Cell samples were then permeabilized and blocked using PBS containing 10 v/v% donkey serum and 0.2 v/v% Triton X-100 at room temperature for 3h. Samples were then washed twice with PBS. Cell samples were then incubated with YAP primary antibody (clone EP1674Y form Abcam, 1:50) in PBS containing 0.2 v/v% Triton X-100 in the fridge overnight. Samples were washed with 0.2 v/v% Triton X-100 in PBS thrice. Alexa 647 donkey anti-rabbit secondary antibody was then added in 0.2 v/v% Triton X-100 in PBS to the cells (1:200) for 2h. Samples were washed with 0.2 v/v% Triton X-100 in PBS thrice. Afterwards, samples were stained with phalloidin to visualize F-actin for 1h (1:400) or with DAPI to visualize cell nuclei for 5 min (1:200) in PBS with 0.2 v/v% Triton X-100 in PBS at room temperature in the dark. Finally, the samples were washed with PBS containing 0.2 v/v% Triton X-100. Immediately thereafter, the samples were imaged (and only mounted if necessary) on a Leica TCS SP5 inverted confocal microscope using 10× (HCX PL APO CS 10.0 × 0.4 DRY UV), 20× (HCX PL APO CS 20.0 × 0.7 DRY UV) and 40× (HCX PL APO CS 40.0 × 1.1 water UV) objectives. Image analysis and quantification of cell area and circularity (n is at least 10 cells per condition) was performed using LAS X and Fiji (ImageJ) software. For YAP nuclear translocation, z-slices were first merged into 1 image. For the YAP channel only, noise speckles (outliers) were removed (pixel size ~ 30-70 pixels (depending on the size of the noise) and threshold ~ 30, see below for example) (Fig. S1). Then, 3 random circular areas (region of interest (ROI) 1, 2 and 3) with a diameter of 5 μm were selected in both the nucleus and cytoplasm. The average fluorescence intensity in all ROIs was determined. YAP nuclear translocation was calculated by dividing the fluorescence intensity of the ROI in the nucleus by the ROI in the cytoplasm. The average of all ROIs was plotted as the YAP nuclear/cytoplasmic ratio (n is at least 5 cells per condition).

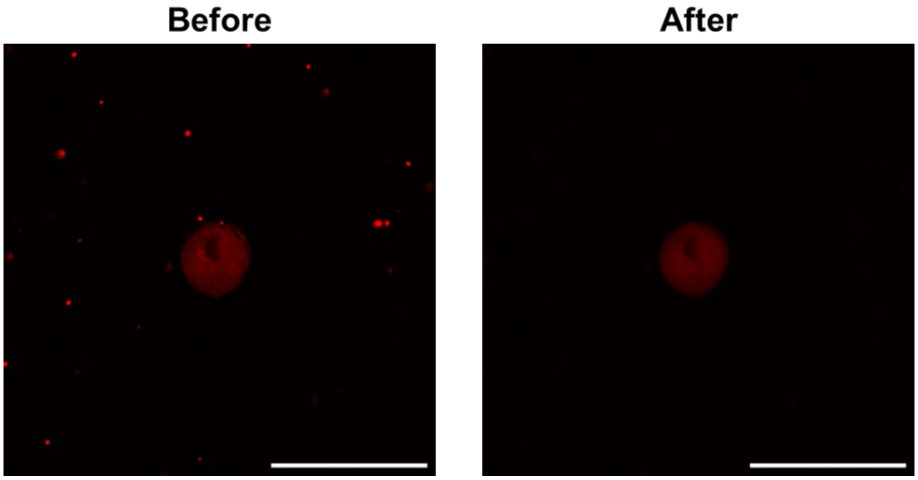

Fig. S1. Quantification of mechanotransduction marker YAP. Outliers were removed as explained above, showing the visualization of YAP staining before (left) and after (right) the processing. YAP in red, scale bar = 50 μm.

2. Synthetic procedures and characterization

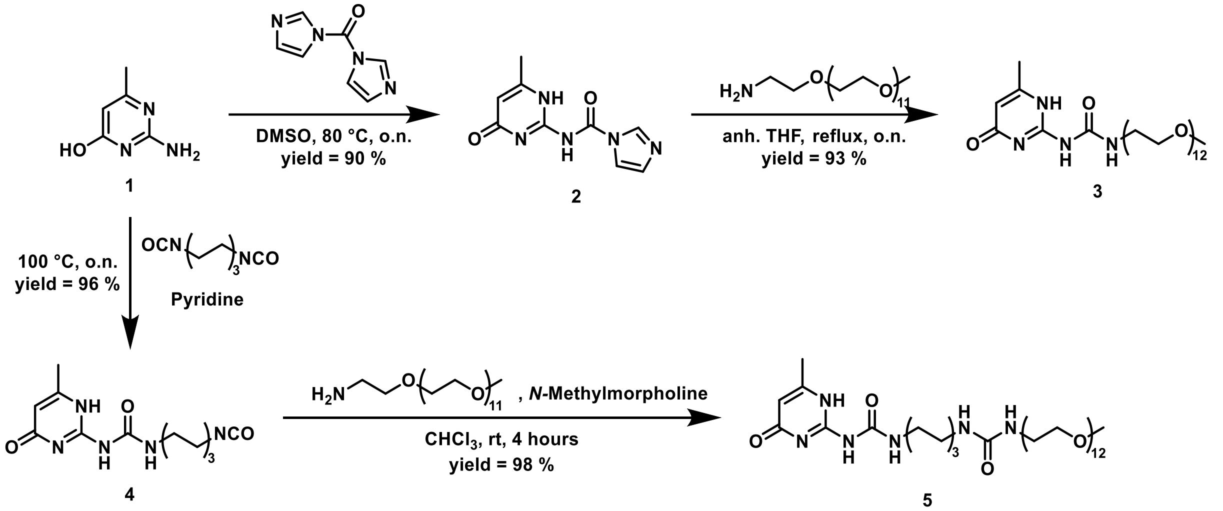

Fig. S2: Synthetic schemes of UPy-OEG12 (UPy*short*-OMe) (3) and UPy-C6-U-OEG12 (UPy*intermediate*-OMe) (5).

_N_-(6-methyl-4-oxo-1,4-dihydropyrimidin-2-yl)-1H-imidazole-1-carboxamide (2)

A round bottom flask was charged with 2-amino-4-hydroxy-4-methylpyrimidine (168 mg; 1.34 mmol), 1, 1'-carbonyldiimidazole (296 mg; 1.83 mmol) and DMSO (2.0 mL). The resulting suspension was stirred overnight at 80 °C under Argon atmosphere. After 20 hours the mixture was cooled down to room temperature and the formed solid was suspended in acetone (5.0 mL), filtered and washed with acetone (3 × 5.0 mL). The final compound was dried under vacuum and 264 mg were received as a white powder in 90% yield. As a consequence of the poor solubility of the received compound in most of the available solvents, it was used directly used in the next step without any characterization.

1-(2,5,8,11,14,17,20,23,26,29,32,35-dodecaoxaheptatriacontan-37-yl)-3-(6-methyl-4-oxo-1,4-dihydropyrimidin-2-yl)urea (3) = UPy-OEG12.

Compound 2 (64 mg; 0.29 mmol) was suspended in anhydrous THF (0.5 mL) and a solution of dodeca(ethylene glycol)amine (119 mg; 0.21 mmol) in anhydrous THF (0.3 mL) was added dropwise while stirring under Argon atmosphere. Additional solvent was added (0.7 mL) and the mixture was heated to reflux overnight while stirring. After 20 hours the mixture was cooled down to room temperature and the excess of 2 was quenched with a drop of aqueous hydrogen chloride (HCl) (2 M). After the solvent was removed under vacuum, water was added (5.0 mL) and the resulting mixture was acidified with aqueous HCl 2 M until pH around 5.0. The aqueous layer was extracted with DCM (3 × 10 mL) and the combined organic layers were dried over $MgSO_4$, filtered and concentrated under vacuum. The crude product was purified through normal-phase flash chromatography (Eluent: DCM/MeOH = 95/5) to afford 139 mg of the desired compound as a wax in 93% yield. ^{1}H-NMR (400 MHz, $CDCl_3$): δ 13.02 (s, 1H), 11.89 (s, 1H), 10.23 (s, 1H), 5.80 (s, 1H), 3.64-3.61 (m, 44H), 3.45 (dt, $J_{HCCH} \sim J_{HCNH}$ = 6.0 Hz, 2H), 3.48-3.43 (m, 2H), 3.37 (s, 3H),

2.22 (s, 3H). ^{13}C-NMR (100 MHz, $CDCl_3$): δ 173.02, 156.92, 154.71, 148.35, 106.86, 72.06, 70.74, 70.70, 70.65, 70.59, 70.43, 69.60, 59.16, 39.68, 19.07. LC-MS: calc.: 710.39 Da, found: $[M+H]^+$ = 711.33, $[M+K]^+$ = 749.17.

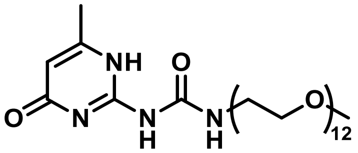

(3) = UPy-OEG$_{12}$ (UPy$_{short}$-OMe)

1-(2-isocyanatoethyl)-3-(6-methyl-4-oxo-1,4-dihydropyrimidin-2-yl)urea (4)

A round bottom flask was charged with 2-amino-4-hydroxy-4-methylpyrimidine (5.0 g; 40 mmol), hexamethylene diisocyanate (100 mL; 680 mmol) and pyridine (1.0 mL; 12 mmol). The resulting mixture was heated to 100 °C overnight under Argon atmosphere while stirring. After 16 hours the mixture was cooled to room temperature, diluted with 57.0 mL of heptane and filtered over buchner filter. During the filtration the buchner filter was covered with a large funnel with a N_2 flow to protect the final product from humidity. The product was washed with heptane (5 × 43 mL) and hexane (2 x 43 ml). The product was finally dried under vacuum to provide 11.3 g of the desired compound as a white powder in 96% yield. ^{1}H-NMR(400 MHz, $CDCl_3$): δ 13.11 (s, 1H), 11.86 (s, 1H), 10.19 (s, 1H), 5.82 (s, 1H), 3.30-3.23 (m, 4H), 2.23 (s, 3H), 1.65-1.58 (m, 6H), 1.48 – 1.33 (m, 2H). ^{13}C-NMR (100 MHz, $CDCl_3$): δ 173.22, 156.75, 154.86, 148.43, 106.85, 43.03, 39.93, 31.34, 29.45, 26.38, 26.32, 19.09.

1-(6-methyl-4-oxo-1,4-dihydropyrimidin-2-yl)-3-(39-oxo-2,5,8,11,14,17,20,23,26,29,32,35-dodecaoxa-38,40-diazahexatetracontan-46-yl)urea (5) = UPy-C$_6$-U-OEG$_{12}$.

Compound 4 (88 mg; 0.3 mmol) was suspended in $CHCl_3$ (4.0 mL) along with *N*-methylmorpholine (60 µL; 0.55 mmol) and a solution of dodeca(ethylene glycol)amine (180 mg; 0.32 mmol) in $CHCl_3$ (2.0 mL) was added dropwise under Argon atmosphere while stirring. The mixture was allowed to stir at room temperature for 5 hours. Product formation was confirmed by the disappearance of the IR isocyanate band at 2270 cm^{-1}. The solvent was removed under vacuum and the crude product was dissolved in $CHCl_3$ (3.0 mL) and precipitated in Et_2O (45 mL). The precipitate was collected through centrifugation (4250 rpm, 10 min). The pellet was finally purified through normal-phase flash-chromatography (Eluent: DCM/MeOH/ethylene glycol dimethyl ether = 8/1/1) to afford 251 mg of the desired compound as a white powder in 98% yield.

^{1}H-NMR (400 MHz, $CDCl_3$): δ 13.12 (s, 1H), 11.84 (s, 1H), 10.11 (s, 1H), 5.82 (s, 1H), 5.23 (s, 1H), 5.10 (s, 1H), 3.64-3.60 (m, 42H), 3.55-3.52 (m, 4H), 3.37-3.33 (m, 5H), 3.23 (dt, $J_{HCCH} \sim J_{HCNH}$ = 6.7 Hz, 2H), 3.14-3.11 (m, 2H), 2.22 (s, 3H), 1.60-1.56 (m, 2H), 1.48-1.45 (m, 2H), 1.36-1.32 (m, 4H).

^{13}C NMR (100 MHz, $CDCl_3$): δ 173.25, 158.81, 156.68, 154.84, 148.46, 106.79, 72.06, 70.81, 70.73, 70.69, 70.65, 70.60, 70.47, 70.41, 70.21, 59.16, 40.35, 40.29, 39.91, 30.33, 29.56, 26.66, 26.59, 19.07.

LC-MS: calc.: 852.51 Da; found: $[M+H]^+$ = 853.42, $[M+Na]^+$ = 875.33, $[M+2H]^{2+}$ = 427.17.

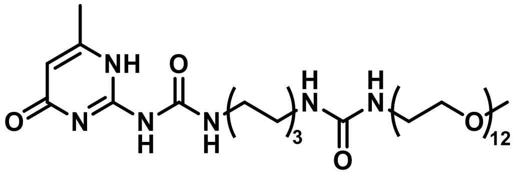

(5) = UPy-C6-U-OEG12 (UPy*intermediate*-OMe)

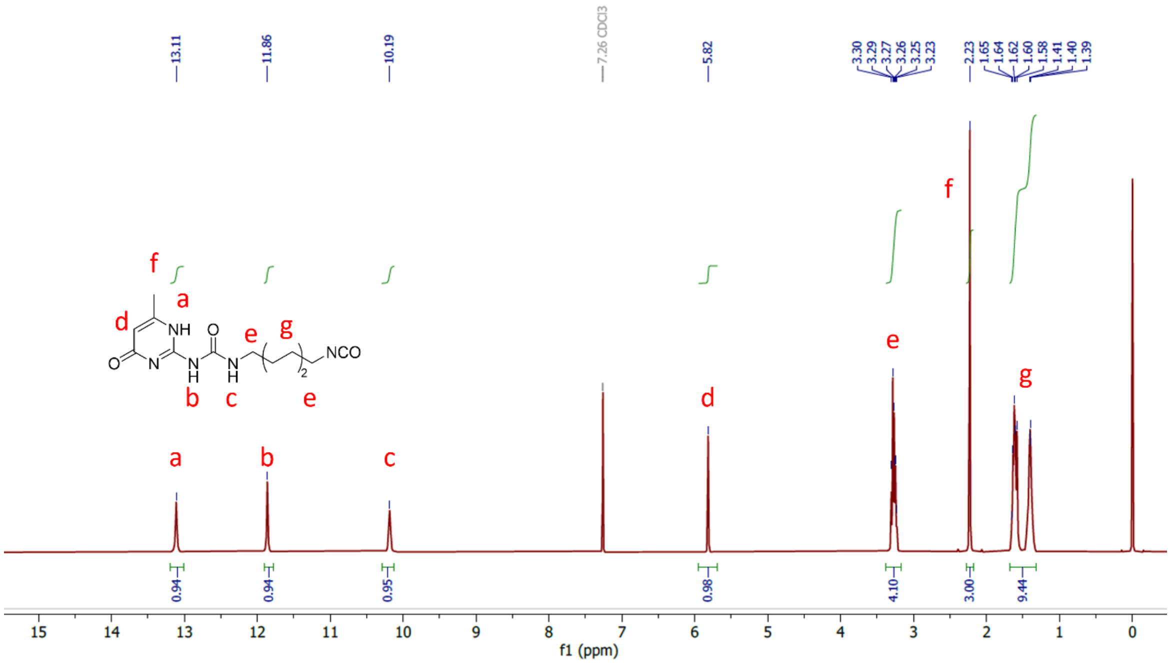

Fig. S3. ^{1}H-NMR (400 MHz) of compound (4) in $CDCl_3$.

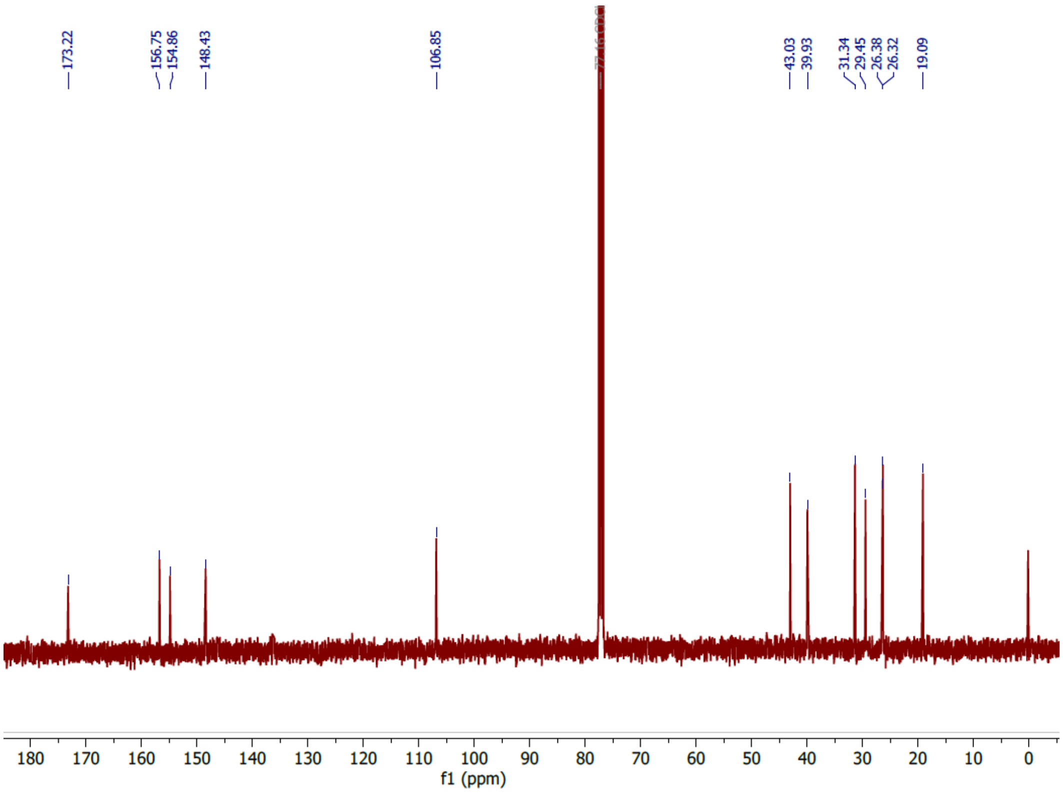

Fig. S4. **^{13}C-NMR (100 MHz) of compound (4) in $CDCl_3$.**

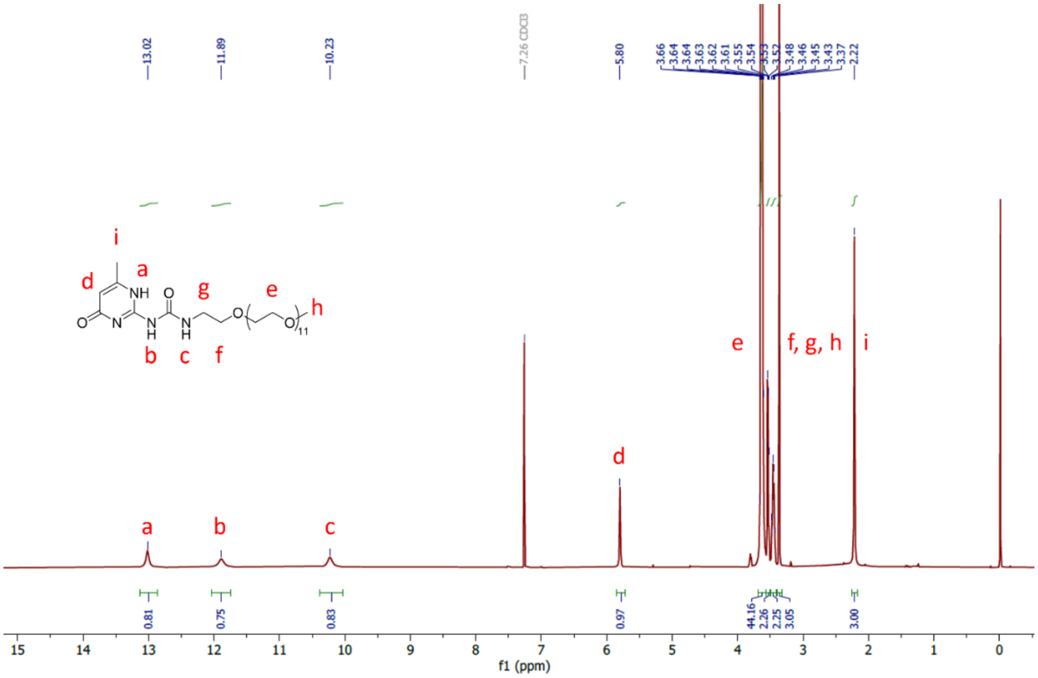

Fig. S5. **^{1}H-NMR (400 MHz) of UPy-OEG$_{12}$ (3) in $CDCl_3$.**

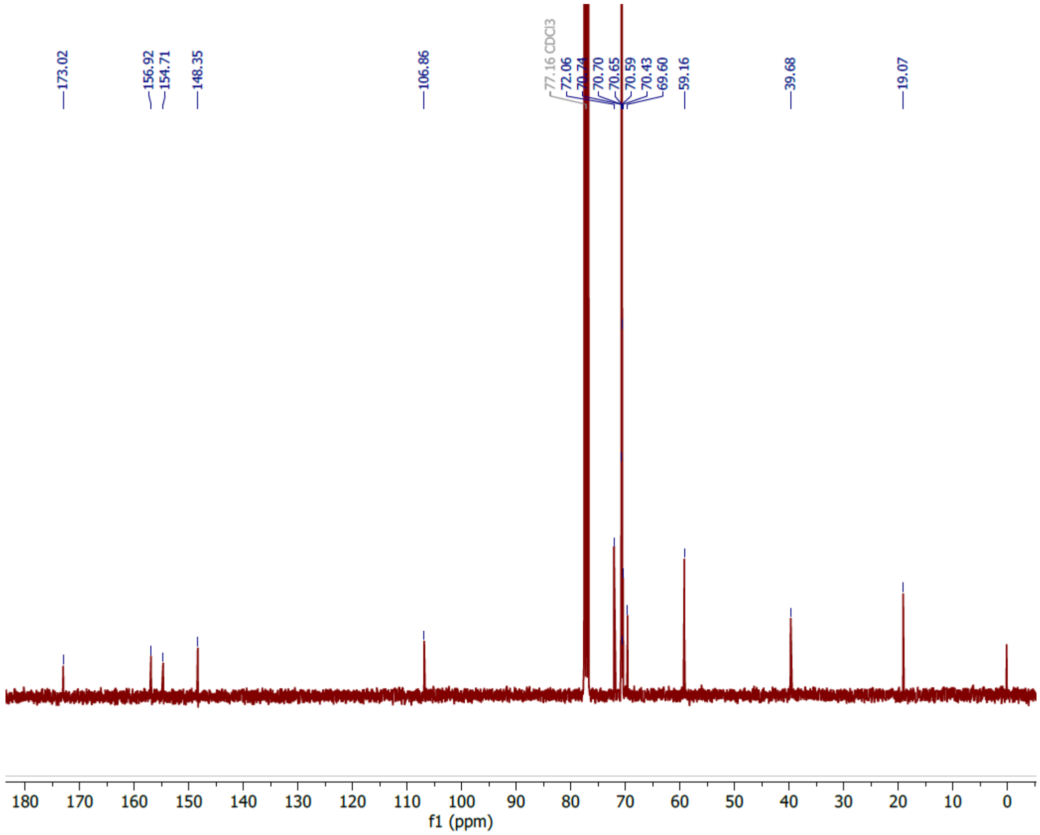

Fig. S6. ^{13}C-NMR (100 MHz) of UPy-OEG$_{12}$ (3) in $CDCl_3$.

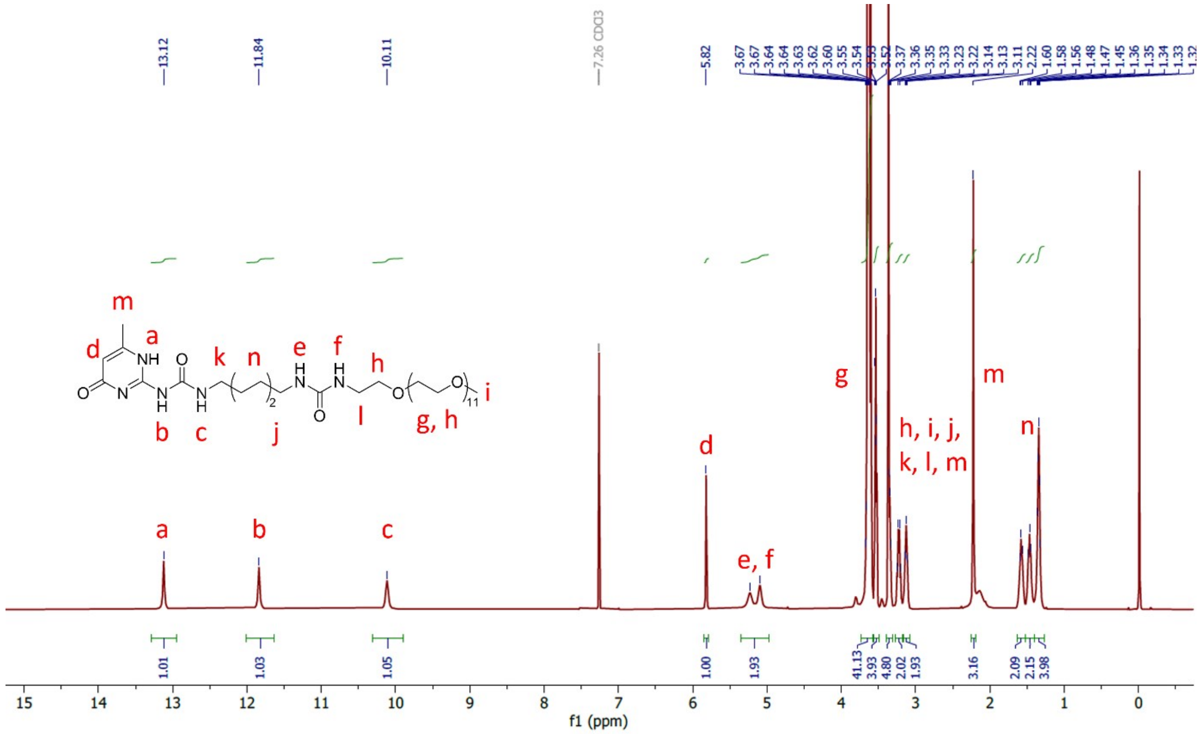

Fig. S7. ^{1}H-NMR (400 MHz) of UPy-C$_6$-U-OEG$_{12}$ (5) in $CDCl_3$.

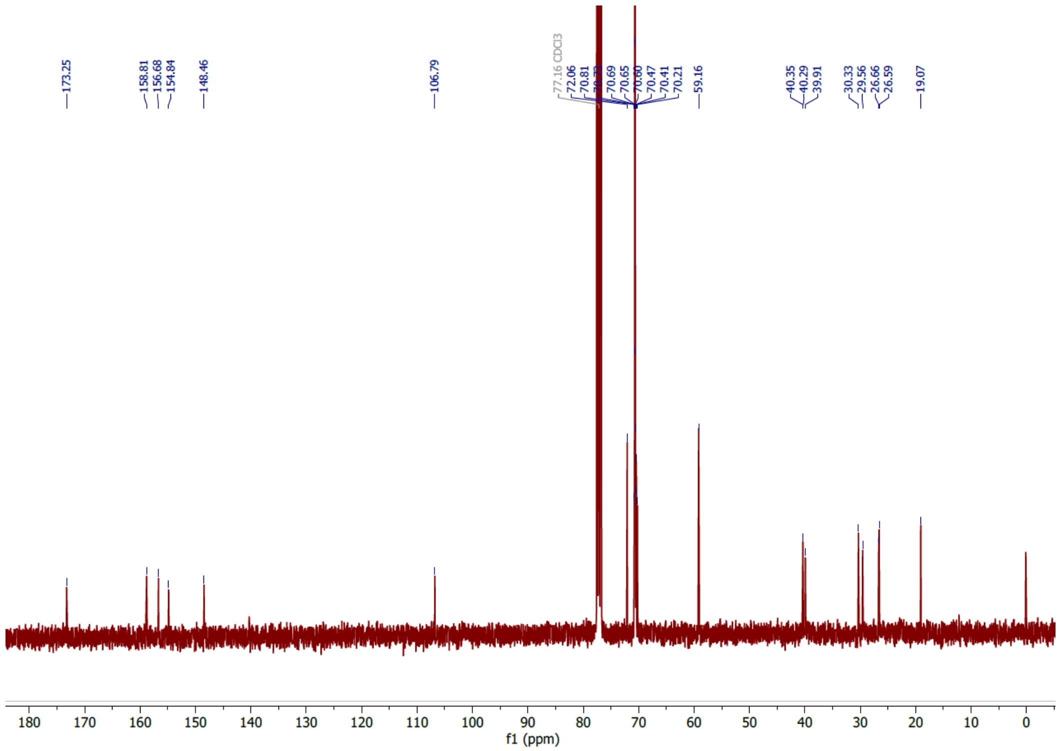

Fig. S8. ^{13}**C-NMR (100 MHz) of UPy-C$_6$-U-OEG$_{12}$ (5) in CDCl$_3$.**

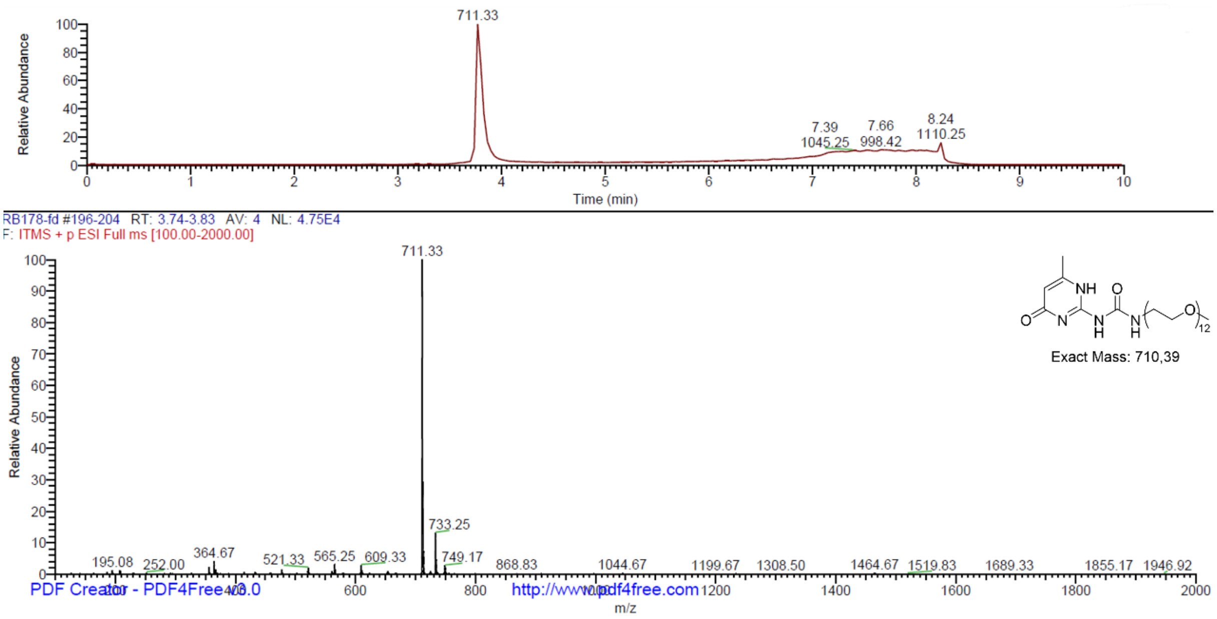

Fig. S9. LC-chromatogram (top) and ESI-MS spectrum(positive; bottom) of UPy-OEG$_{12}$ (3)*. Calc: 710.39 Da*, found: $[M+H]^+$ = *711.33,* $[M+Na]^+$ = *733.25.*

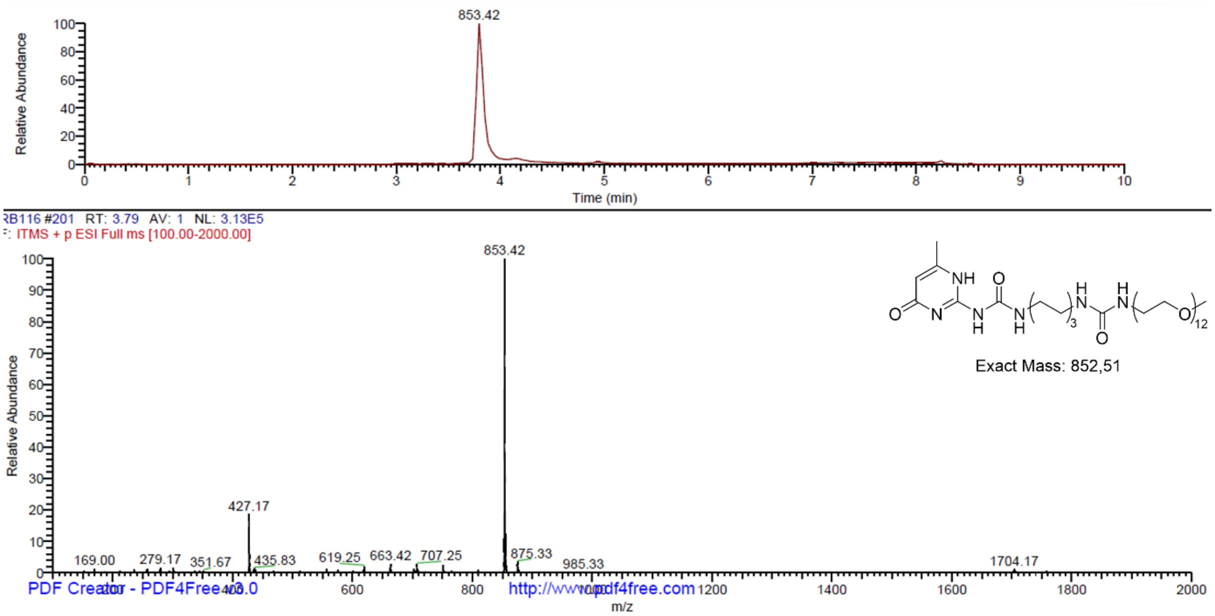

Fig. S10. LC-chromatogram (top) and ESI-MS (positive; bottom) of UPy-C$_6$-U-OEG$_{12}$ (5). Calc: 852.51 Da, found: $[M+H]^+$ = 853.42, $[M+Na]^+$ = 875.33, $[M+2H]^{2+}$ = 427.17.

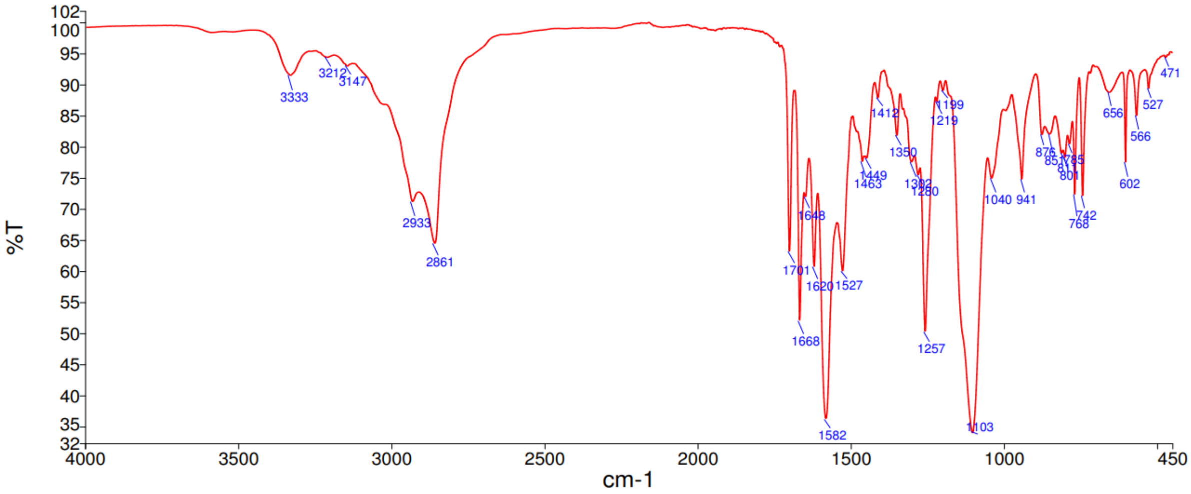

Fig. S11. FT-IR spectrum of UPy-C$_6$-U-OEG$_{12}$ (5).

3. Characterization of single networks in dilute state

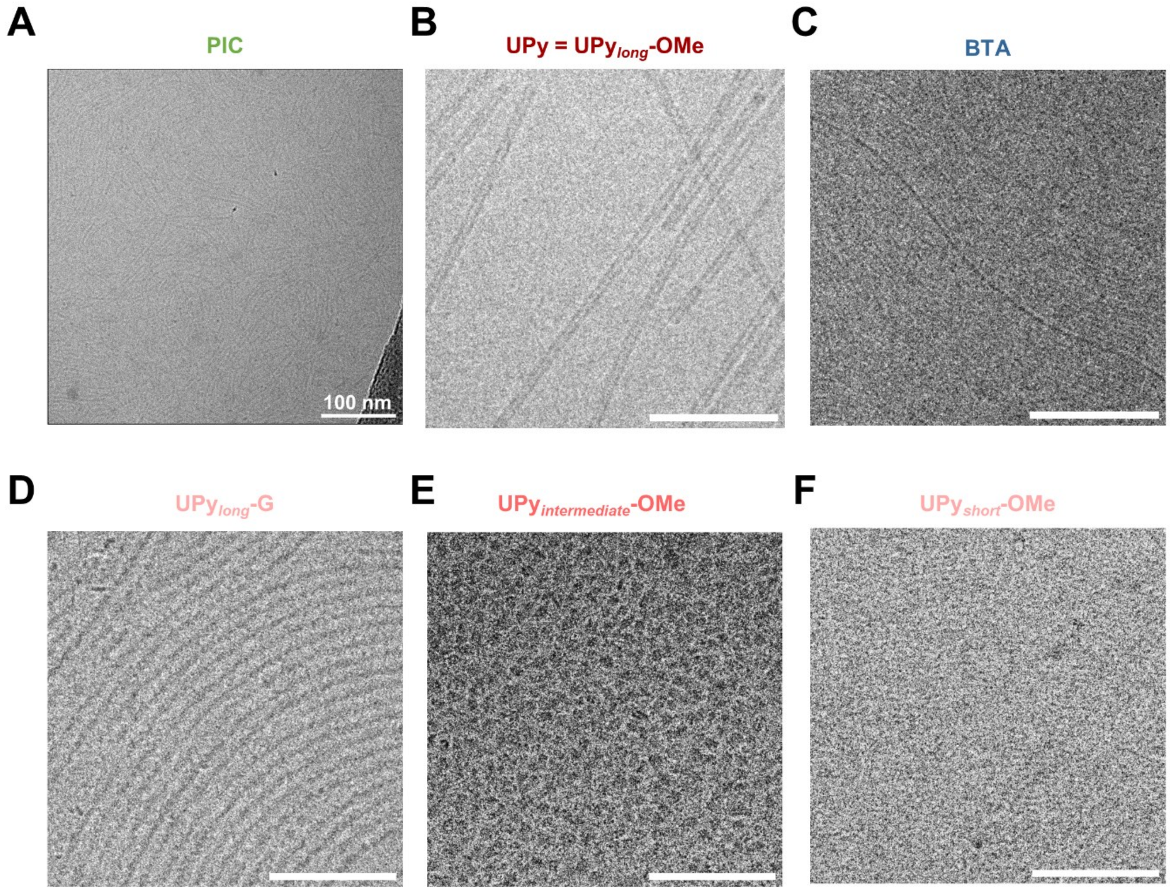

Fig. S12. Cryo-TEM images of the separate single components. 5 times lower concentrations were used than gels. **(A)** PIC (0.01 w/v%) formed short thin fingerprint fibers. **(B)** UPy (0.05 w/v%) formed long, bundled fibers. **(C)** BTA (0.06 w/v%) formed two-component helical fibers. **(E)** UPy$_{long}$-G (0.06 w/v%) formed long fibers. **(F)** UPy$_{intermediate}$-OMe (0.03 w/v%) formed micelles and **(G)** UPy$_{short}$-OMe (0.03 w/v%) formed no large structures.

4. Characterization of single networks in hydrogel state

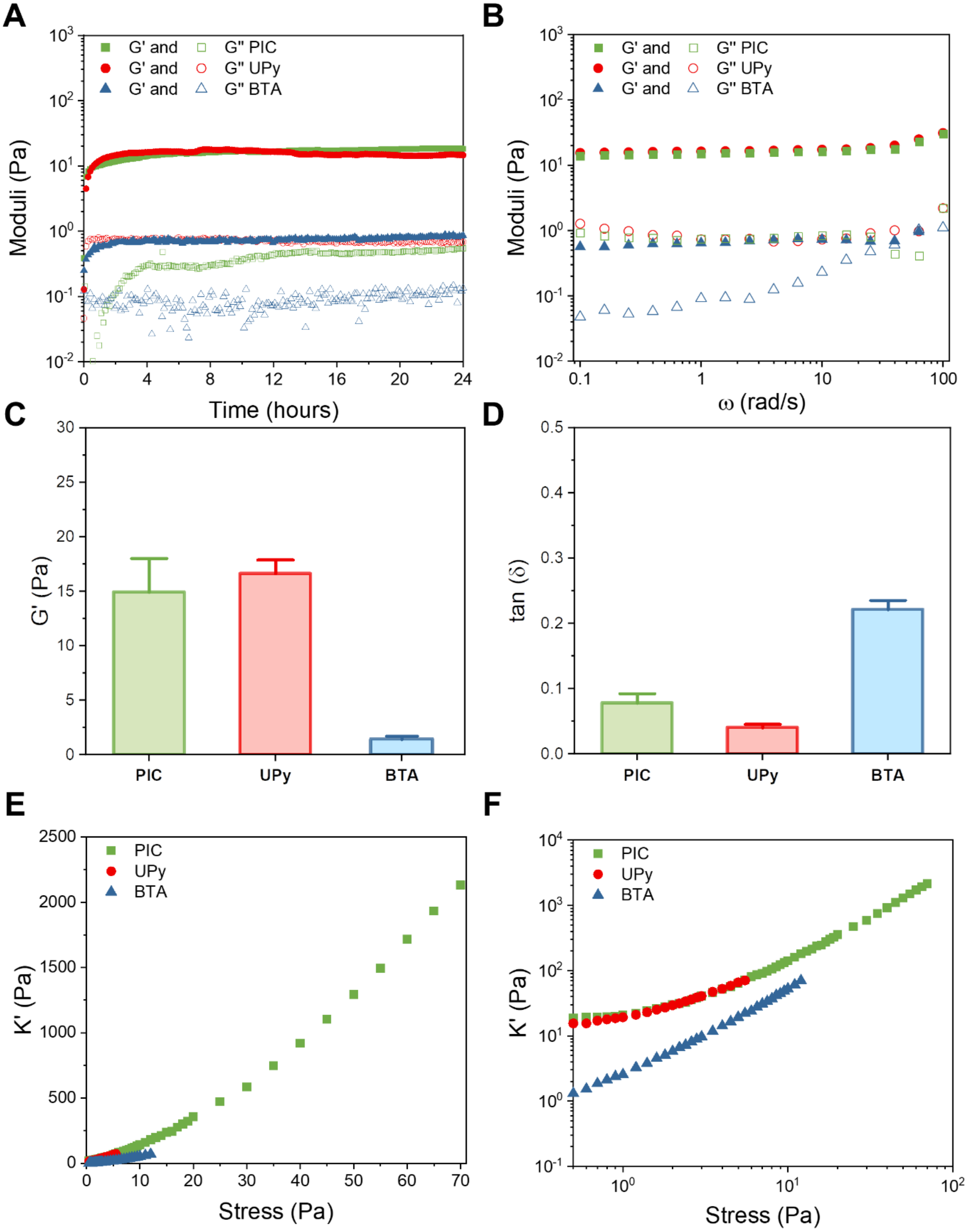

Fig. S13. Mechanical properties of the single networks. (A) Formation of **PIC, UPy** and **BTA** followed over time at 37 °C at ω =1 rad/s and y = 1%. **(B)** Frequency response at y = 1%. **(C)**

Quantification of G’ at 1 rad/s and y = 1%**.** **(D)** and of tan δ. **(E, F)** Stress -stiffening behavior of the single-component networks shown at linear scale **(E)** and log-scale **(F)**.

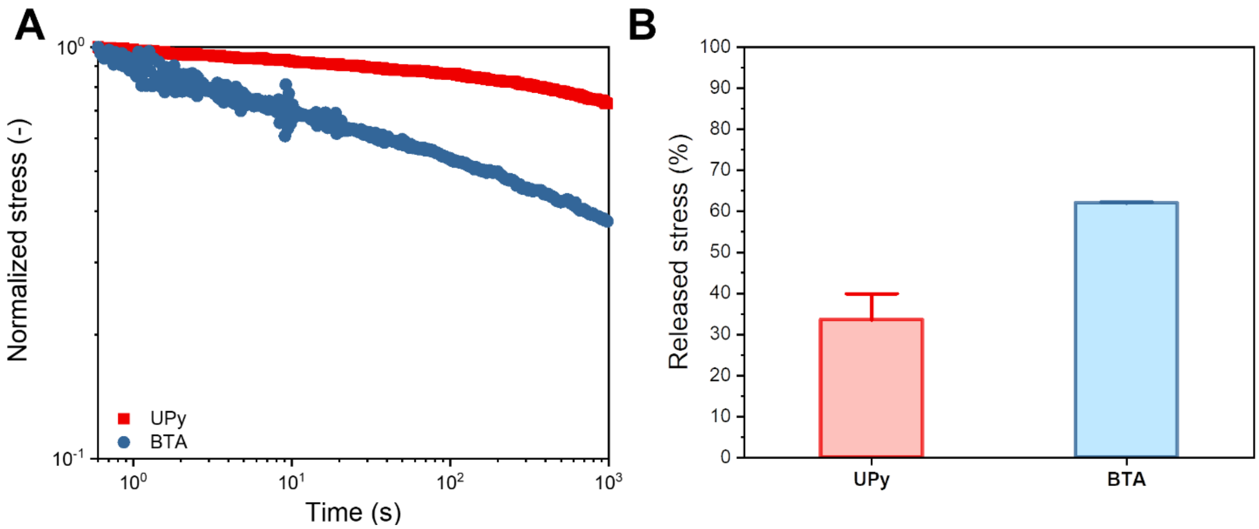

Fig. S14. Dynamic behavior of UPy (0.24 w/v%) and BTA (0.30 w/v%). (A) Stress relaxation measurements with 7.5% strain. **B)** Quantification of released stress after 1000 seconds.

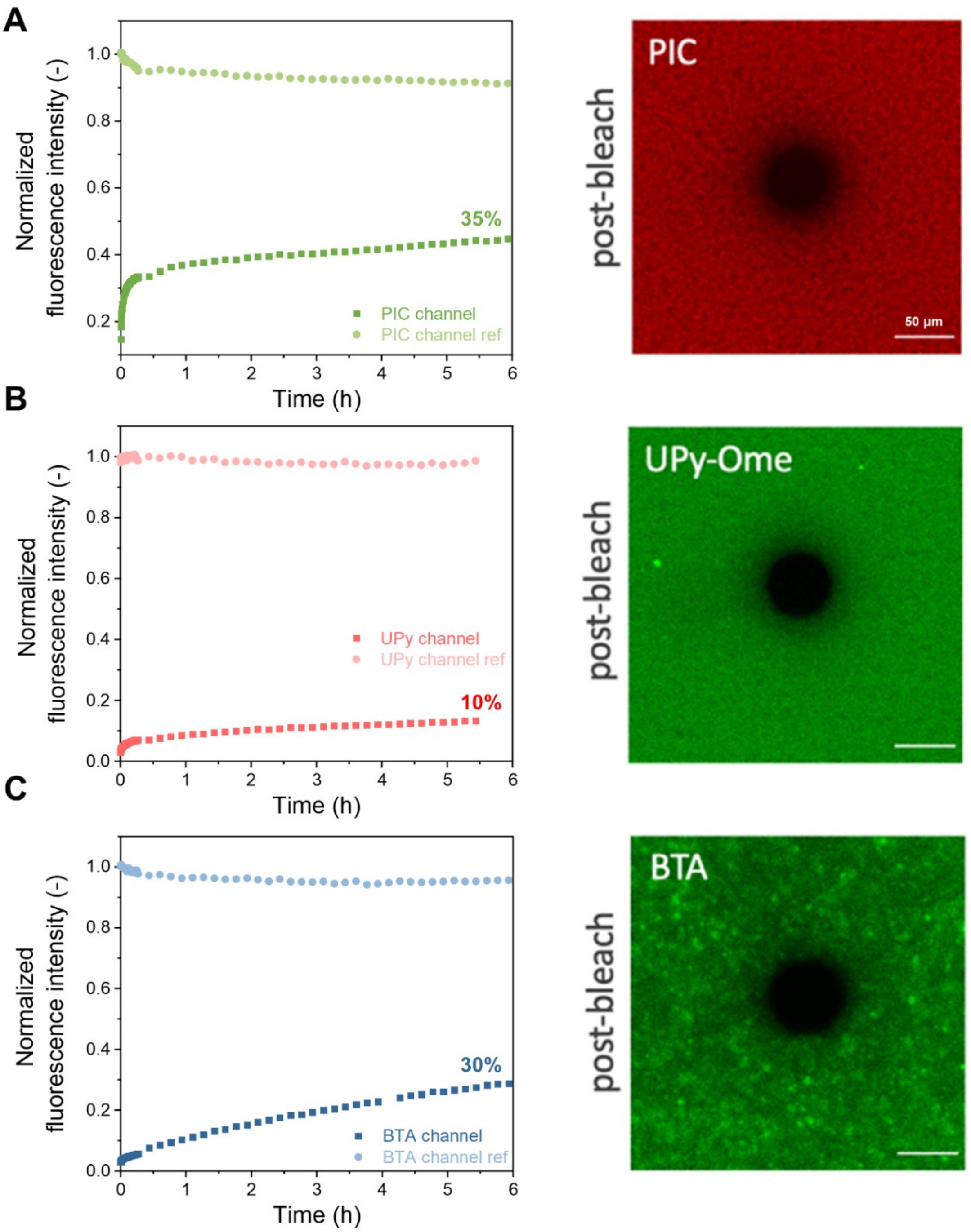

Fig. S15. Local dynamical properties of single networks as measured with FRAP, showing the fluorescence recovery over time and the post-bleach region. PIC (0.05 w/v%) with a tetramethylrhodamine (TAMRA) dye (15 µM) and UPy (0.24 w/v%) with UPy-Cyanine 5 (UPy-Cy5) (15 µM) form rigid networks. This in contrast to BTA (0.30 w/v%) with BTA-Cy5 (15 µM), which is more dynamic. Scale bar = 50 µm.

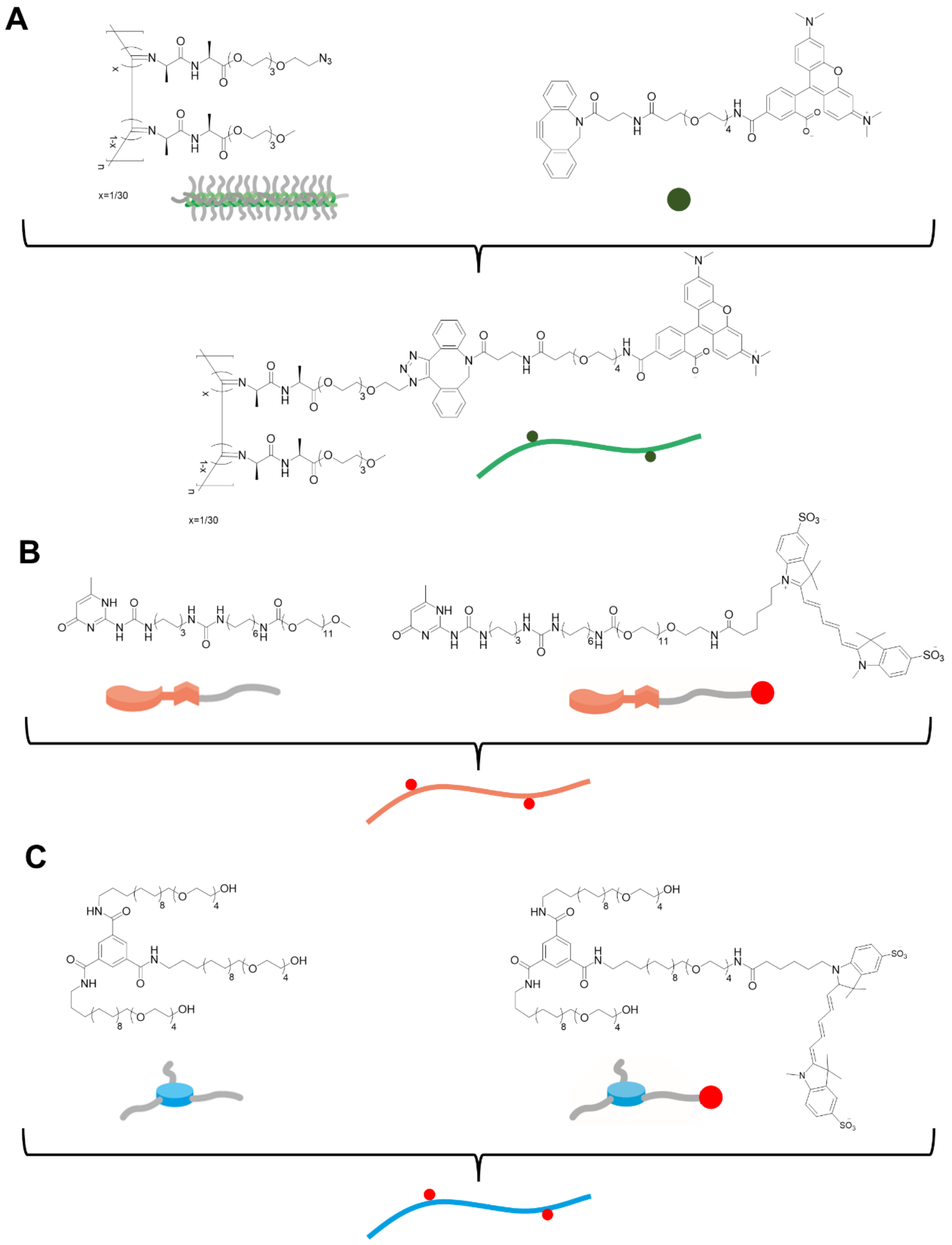

Fig. S16. Molecular structures of incorporated of fluorescent dyes. (A) PIC-N_3 is covalently attached to DBCO-TAMRA dye. **(B)** UPy molecules are mixed with UPy-Cy5, which is incorporated into the fibers via non-covalent interactions. **(C)** BTA molecules are mixed with BTA-Cy5, which is incorporated into the fibers via non-covalent interactions.

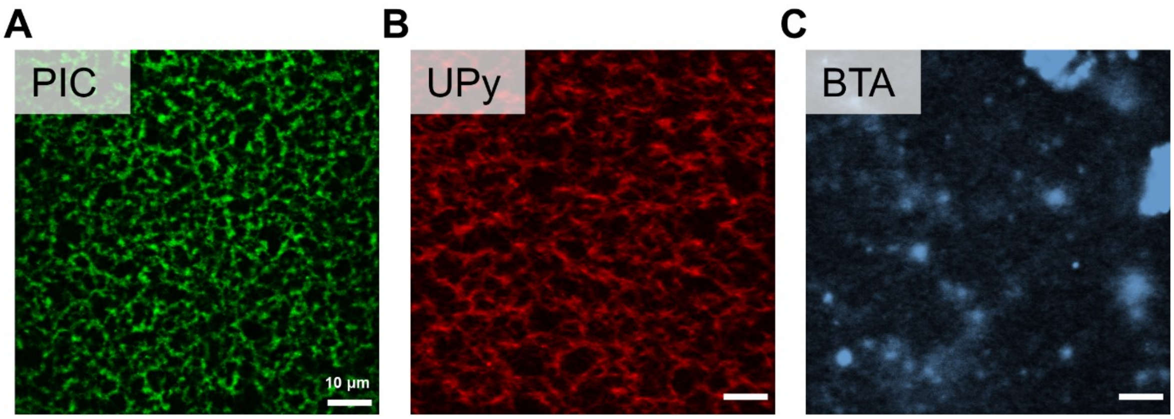

Fig. S17. Confocal image of the single networks. (A) PIC (0.05 w/v%), functionalized with a TAMRA dye (15 µM), showing a mesh like network with small pores. **(B)** UPy (0.24 w/v%), mixed with UPy-Cy5 (15 µM), showing again a mesh like network, although with larger pores than the PIC, looking more liquid-like. **(C)** BTA (0.30 w/v%), mixed with BTA-Cy5 (15 µM), showing a liquid network with clustered regions of dye. Scale bar=10 µm.

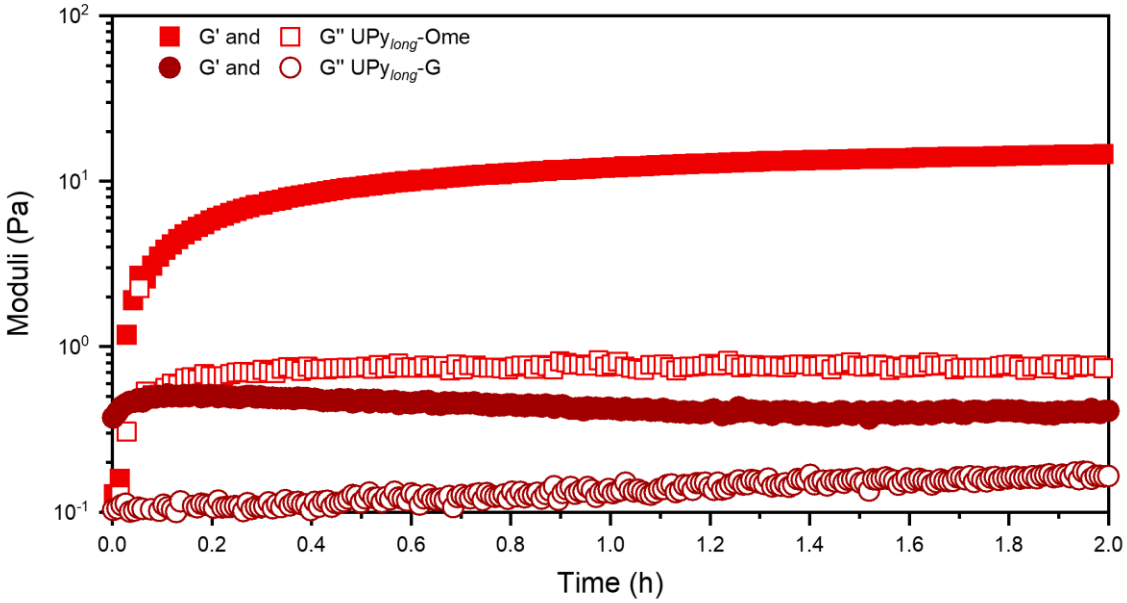

Fig. S18. Formation of single networks with changing UPy-end group. Comparing formation of UPy$_{long}$-OMe (0.24 w/v%) and UPy$_{long}$-G (0.28 w/v%). While UPy$_{long}$-OMe is able to form a soft gel over time, UPy$_{long}$-G remains a liquid (G' < 1 Pa).

5. Characterization of two-component networks in dilute state

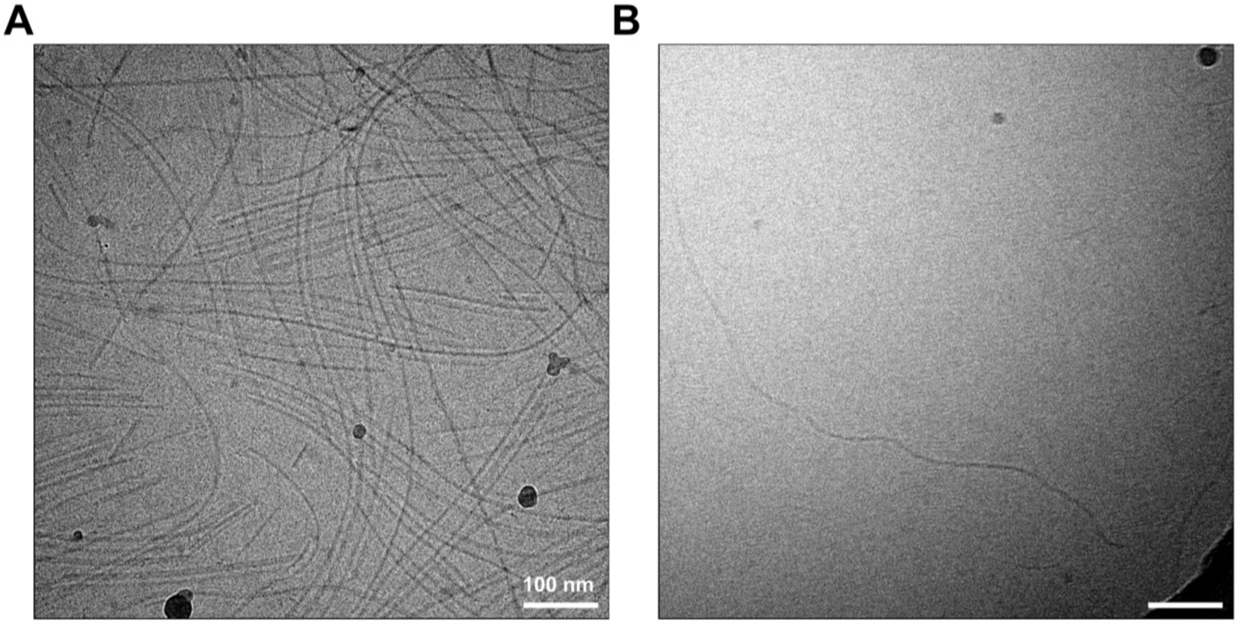

Fig. S19. Cryo-TEM images of hybrid networks. 5 times lower concentrations were used than gels: PIC (0.01 w/v%), UPy (0.05 w/v%) and BTA (0.06 w/v%). **(A)** Hybrid networks showed mostly the morphology of the long supramolecular fibers for the PIC+UPy and **(B)** small fibers for the PIC+BTA network.

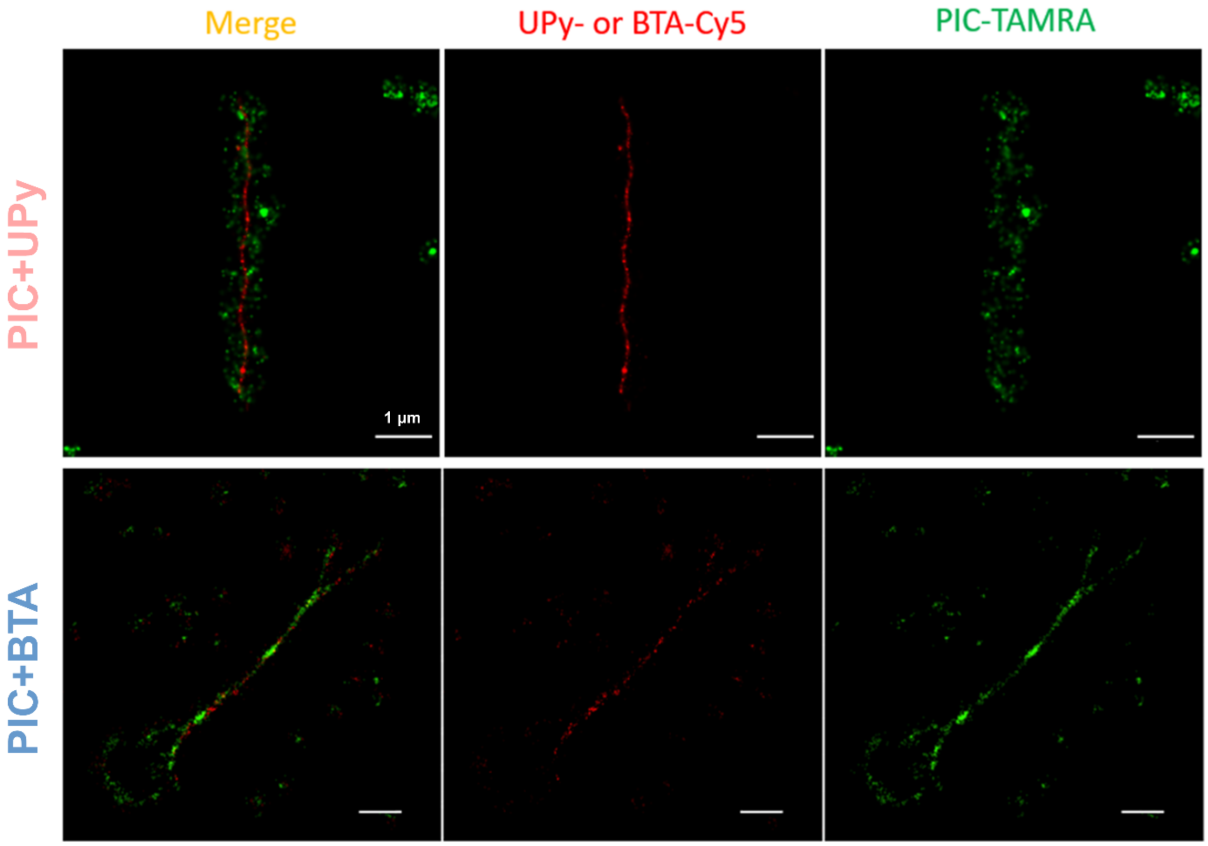

Fig. S20. Super-resolution images in dilute state of PIC+UPy and PIC+BTA using STORM to further elucidate the interaction at nano/micro-meter scale. Concentrations are 550 times diluted compared to the gel state: PIC (2.9 µM), UPy and BTA (4.4 µM). UPy fiber seems to assemble in the PIC helix or in close-proximity. Co-assembled fibers are able to form in the PIC+BTA mix, revealing that the phase-separation observed in gel state is concentration-dependent. Scale bar = 1 µm.

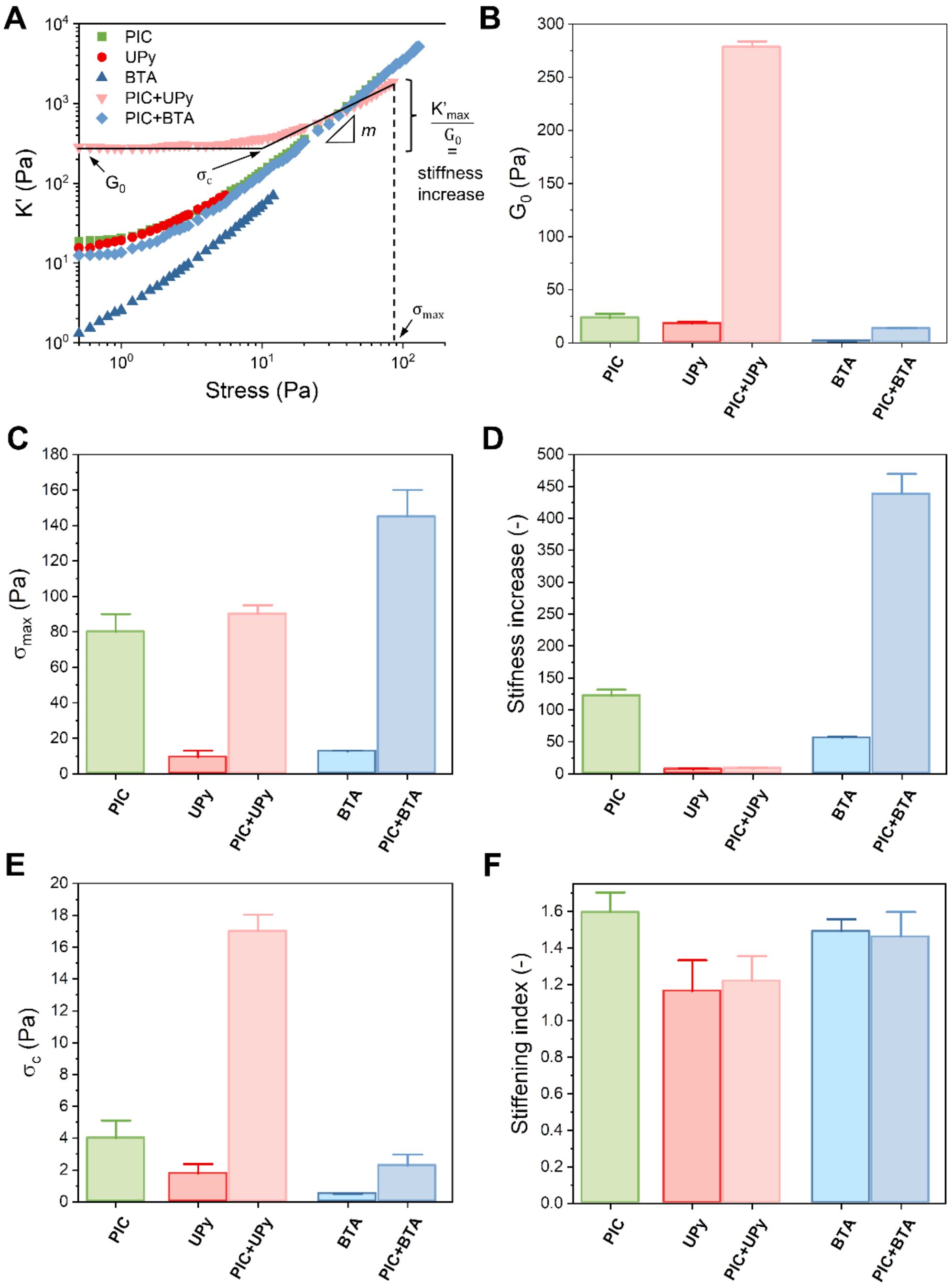

Fig. S21. Stress stiffening quantification of the hybrid and single networks. (A) Explanation of the stress-stiffening parameters and the stress-stiffening response of single and the hybrid networks. **(B)** Plateau modulus extracted from the stress-stiffening response. **(C)** Maximum stress, measure of the stress resistance. **(D)** Stiffness increase, which is quantified by comparing the maximum obtained stiffness before rupture to the plateau modulus, showing a very large increase when BTA is added to PIC. **(E)** Critical stress, a measure for the sensitivity, i.e. the start of the stress-stiffening response. **(F)** Stiffening index, a measure for the responsiveness of the stress stiffening.

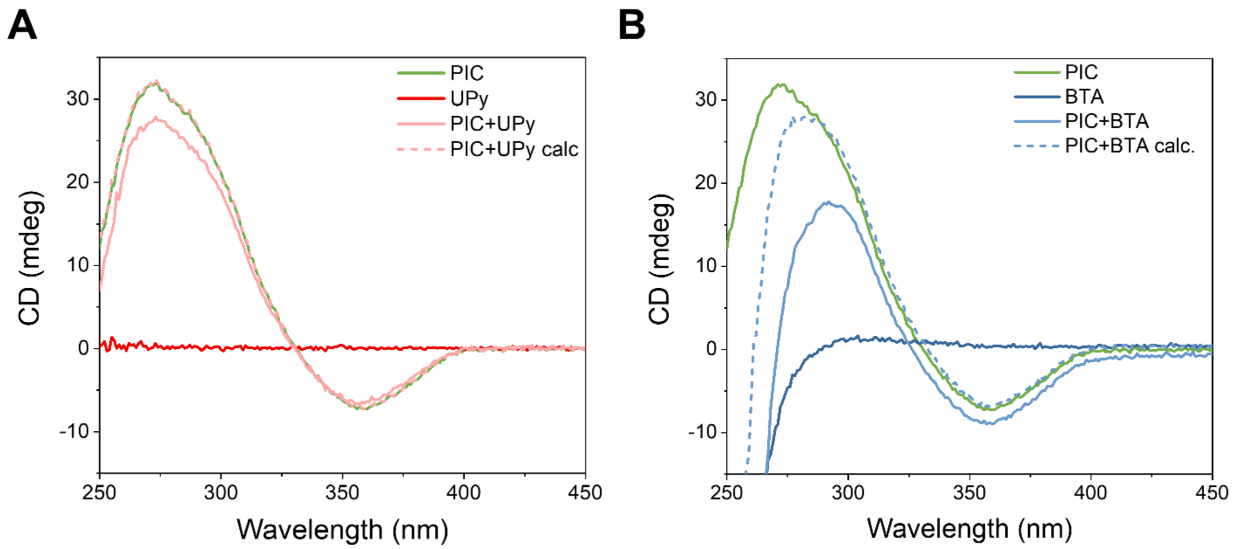

Fig. S22. Investigation of structural changes induced by UPy and BTA on PIC. CD measurements are performed at gel concentration: PIC (0.05 w/v%), UPy (0.24 w/v%) and BTA (0.30 w/v%). (A) CD measurement, showing that addition of UPy decreases helicity of the PIC, indicating UPy interacts with the PIC backbone and strengthens the network. **(B)** Addition of BTA enhances the helicity of the PIC, observed at 360 nm, indicating an additional mechanism which can dissipate energy and enhance the stress-stiffening response of the PIC.

6. Model predicting the structure of two-component networks

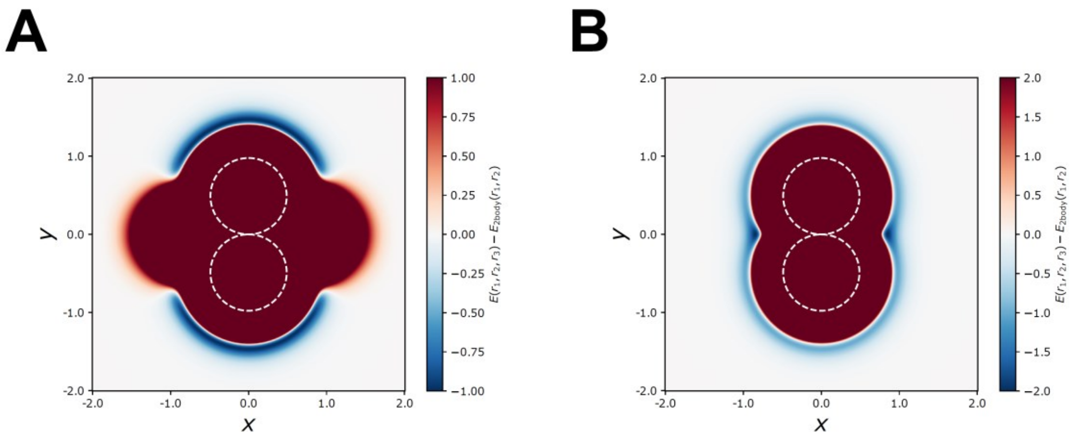

Fig. S23. Representation of the energy function inspired by Bantawa *et al.* (51) (A) Energy profile for the standardpotential. The white circles indicate the location of two particles whose centers are in contact and located at $\vec{r}_1 = (0, -\sqrt{9/32}d_2, 0)$ and $\vec{r_2} = (0, \sqrt{9/32}d_2, 0)$, while the position of the center of the third one is at $\vec{r} = (x, y, 0)$. The color indicates the value of $\Delta E(\vec{r}_1, \ \vec{r}_2, \vec{r}_3) = E(\vec{r}_1, \ \vec{r}_2, \vec{r}_3) - E_{2-body}(\vec{r}_1, \ \vec{r}_2)$, that is the energy of the three particles combined relative to the attractive energy between the two particles shown as white circles. **(B)** Same as **(A)**, but for a potential lacking the three-body interaction. Note that the energy scale is twice as large as the one in panel **(A)**.

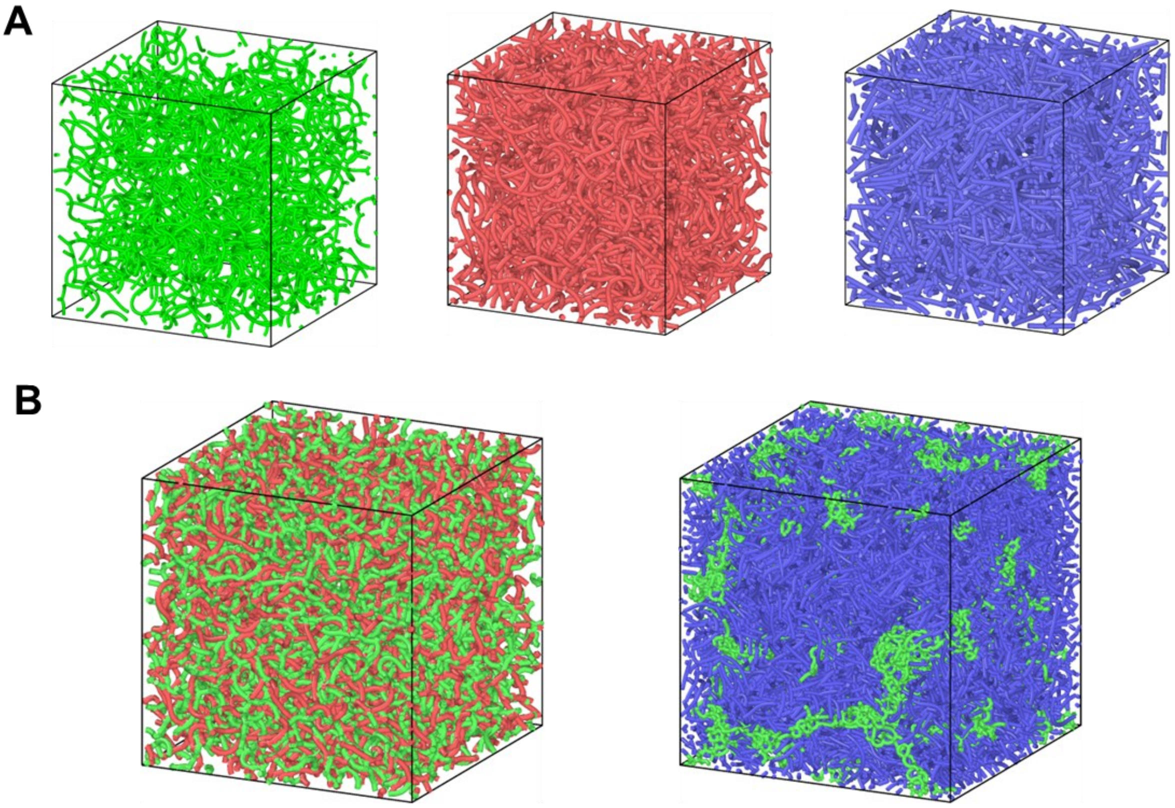

C

		0 Contact	1 Contact	2 Contacts	3 Contacts	4 Contacts	5 Contacts	6 Contacts
PIC+UPy 13500 PIC 54000 UPy	PIC-PIC	0	38	12336	1126	0	0	0
	UPy-UPy	0	108	53796	96	0	0	0
	All-All	0	72	38407	5666	**18901**	**4392**	**62**
PIC+BTA 13500 PIC 54000 BTA	PIC-PIC	0	12	8096	5392	0	0	0
	BTA-BTA	331	11676	41987	6	0	0	0
	All-All	330	11668	50104	5398	0	0	0

Fig. S24. Networks simulated using coarse-grained molecular dynamic simulations. (A) Single networks of PIC (left), a regular network with many branching points, UPy (middle), a fiber like network and BTA (right), an almost liquid-like network. **(B)** Formed two-component networks of PIC+UPy yielding a single network and PIC+BTA yielding a phase separated network. **(C)** Table showing the amount of contact points, where the robust UPy fibers are able to form many more interactions.

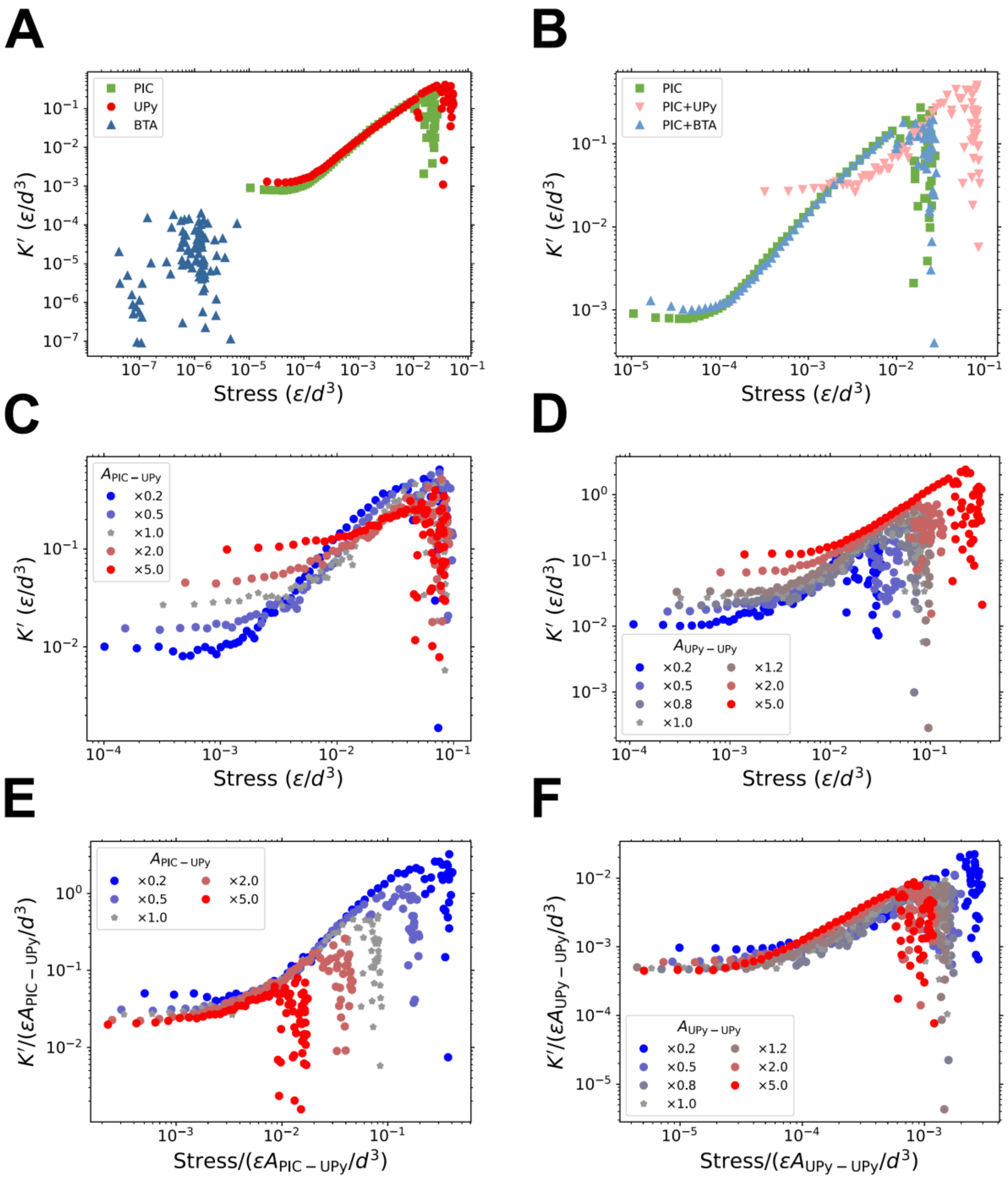

Fig. S25. Differential modulus as a function of stress, with PIC (green squares), UPy (red circles), and BTA (blue triangles). Briefly, panel **(A)** shows the results obtained for single networks, whereas two-component network differential moduli are shown in panel **(B)**. Panels **(C)** and **(D)** show the changes in K' when the PIC-UPy and UPy-UPy interactions are altered by multiplicative factors as shown in the legends. **(E, F)** Differential modulus and stress rescaled by $\epsilon A/d^3$.

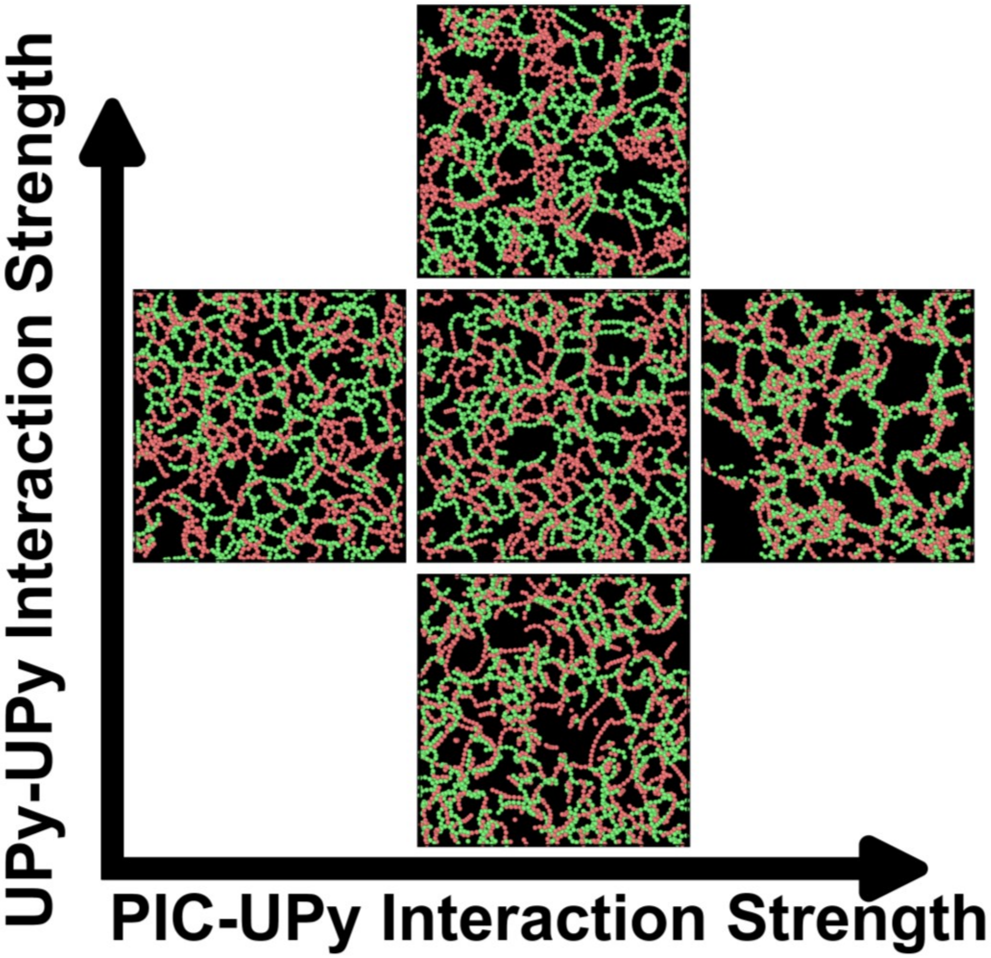

Fig. S26. Slices of the network upon changing UPy-UPy or PIC-UPy interaction strength. In each image, green refers to PIC and red to UPy. The image portrays a slice of the gel of thickness 5d and area equal to the whole periodic box. The images were made using Ovito.*(59)*

7. Characterization of two-component networks in hydrogel state

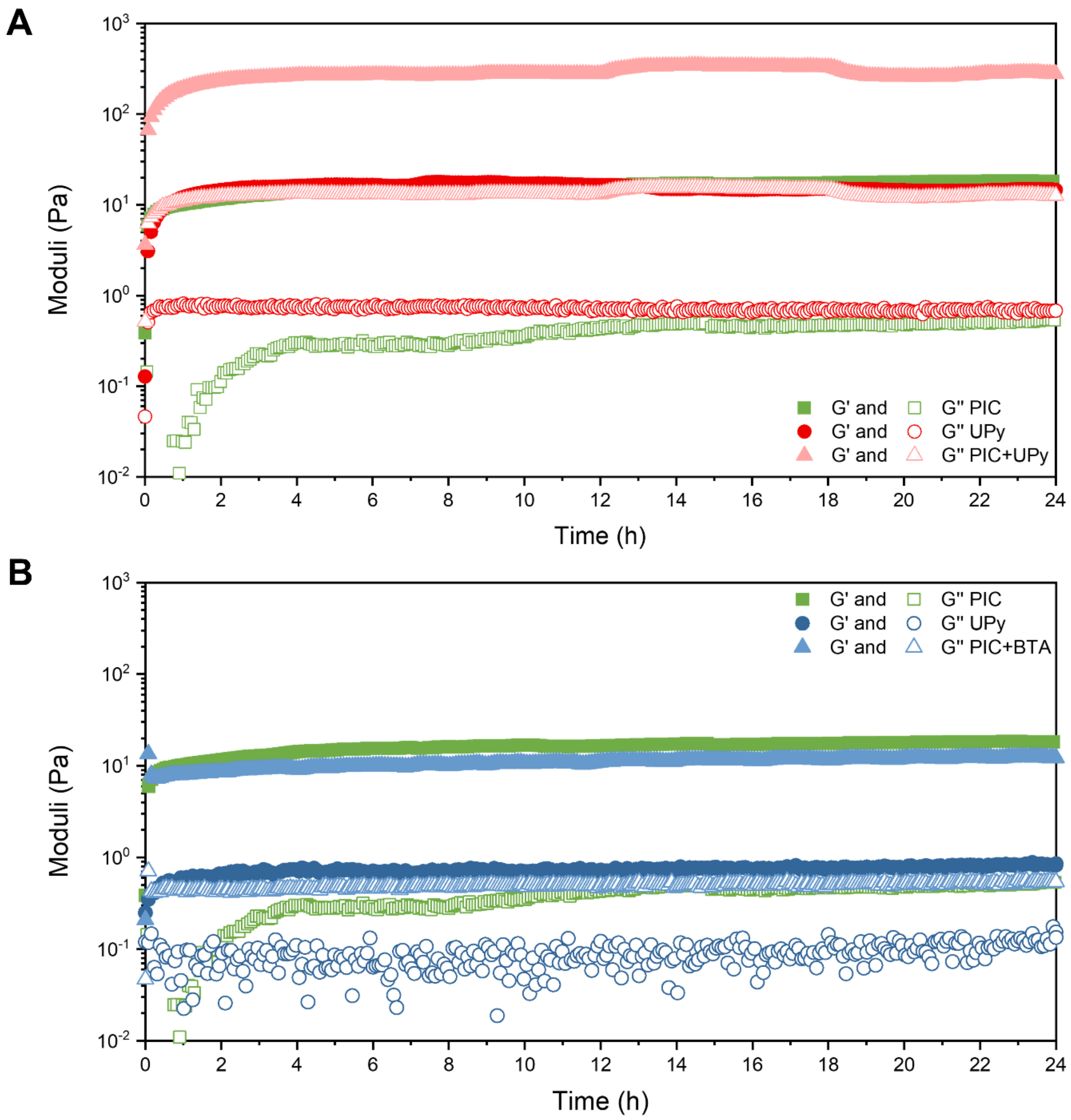

Fig. S27. Formation of the hybrid networks. (A) Time sweep at 37 °C, at 1% strain and 1 rad/s, showing the PIC+UPy (0.05 w/v% + 0.24 w/v%) network is formed within a few hours and becomes much stiffer than the separate components. **(B)** Time sweep following the formation of PIC+BTA (0.05 w/v% + 0.30 w/v%) at 37 °C, at 1% strain and 1 rad/s. Similarly, formation of the hybrid network is completed within a few hours, but no enhanced stiffness is obtained.

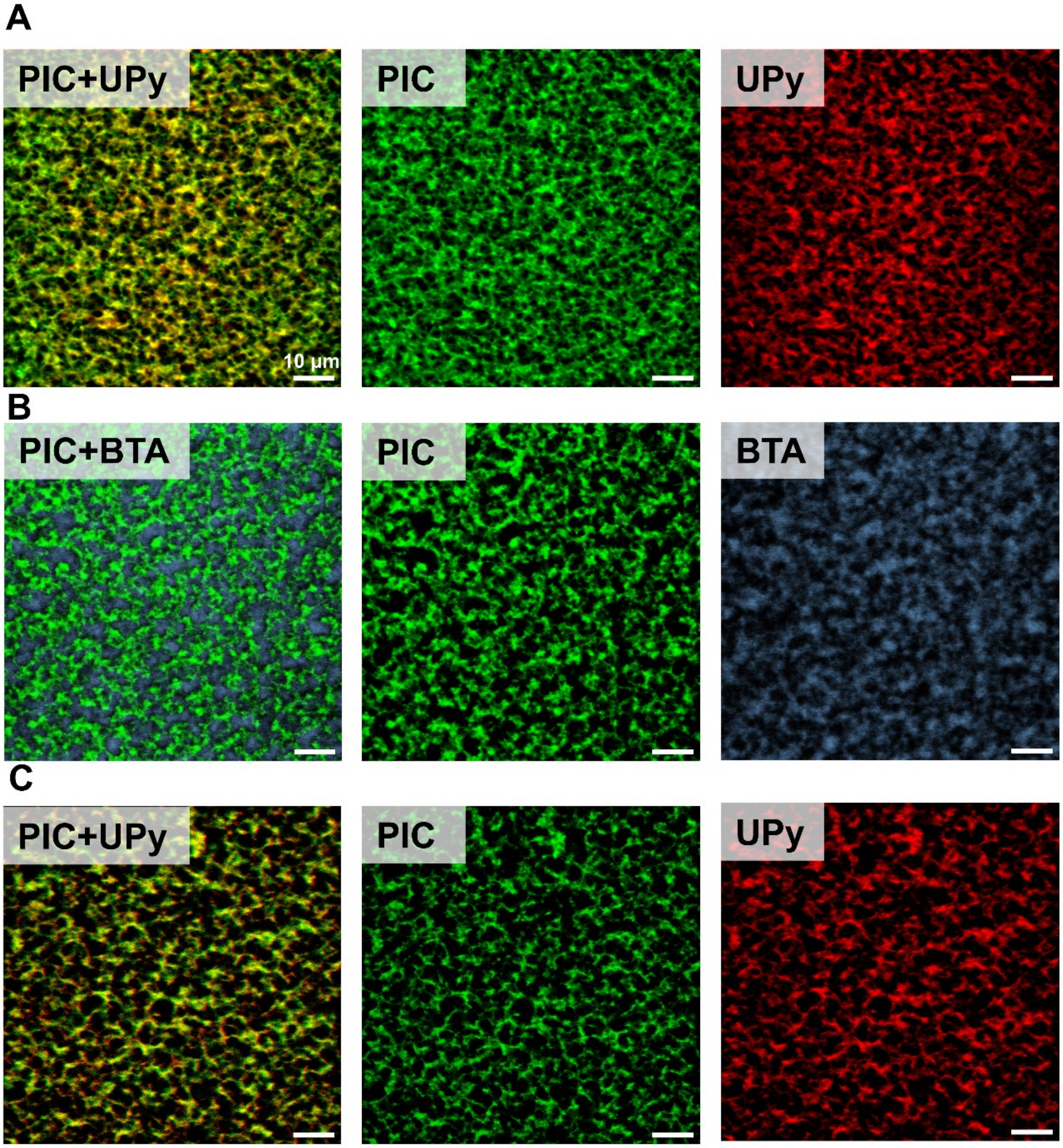

Fig. S28. Structural characterization of hybrid networks, showing the overlay and separate channels. (A) PIC+UPy mixture, composed of PIC (0.05 w/v%) functionalized with a TAMRA dye (15 µM) and UPy (0.24 w/v%) mixed with UPy-Cy5 (15 µM). Mixture shows formation of a single network (left). Both PIC channel (middle) and UPy channel (right) show a similar mesh-like structure with small pores. **(B)** PIC+BTA mixture, composed of PIC (0.05 w/v%) functionalized with a TAMRA dye (15 µM) and BTA (0.30 w/v%) mixed with BTA-Cy5 (15 µM). The mixture forms a two-component network (left). Both channels, PIC (middle) and BTA (right) form a mesh like network, with the BTA network forming in the pores of the PIC. **(C)** Control experiment for PIC+UPy, using UPy-Cy5 dissolved in MeOH, using a similar solvent as BTA-Cy5, confirming that formation of single or two-component networks is not caused by the used solvent. Scale bar = 10 µm.

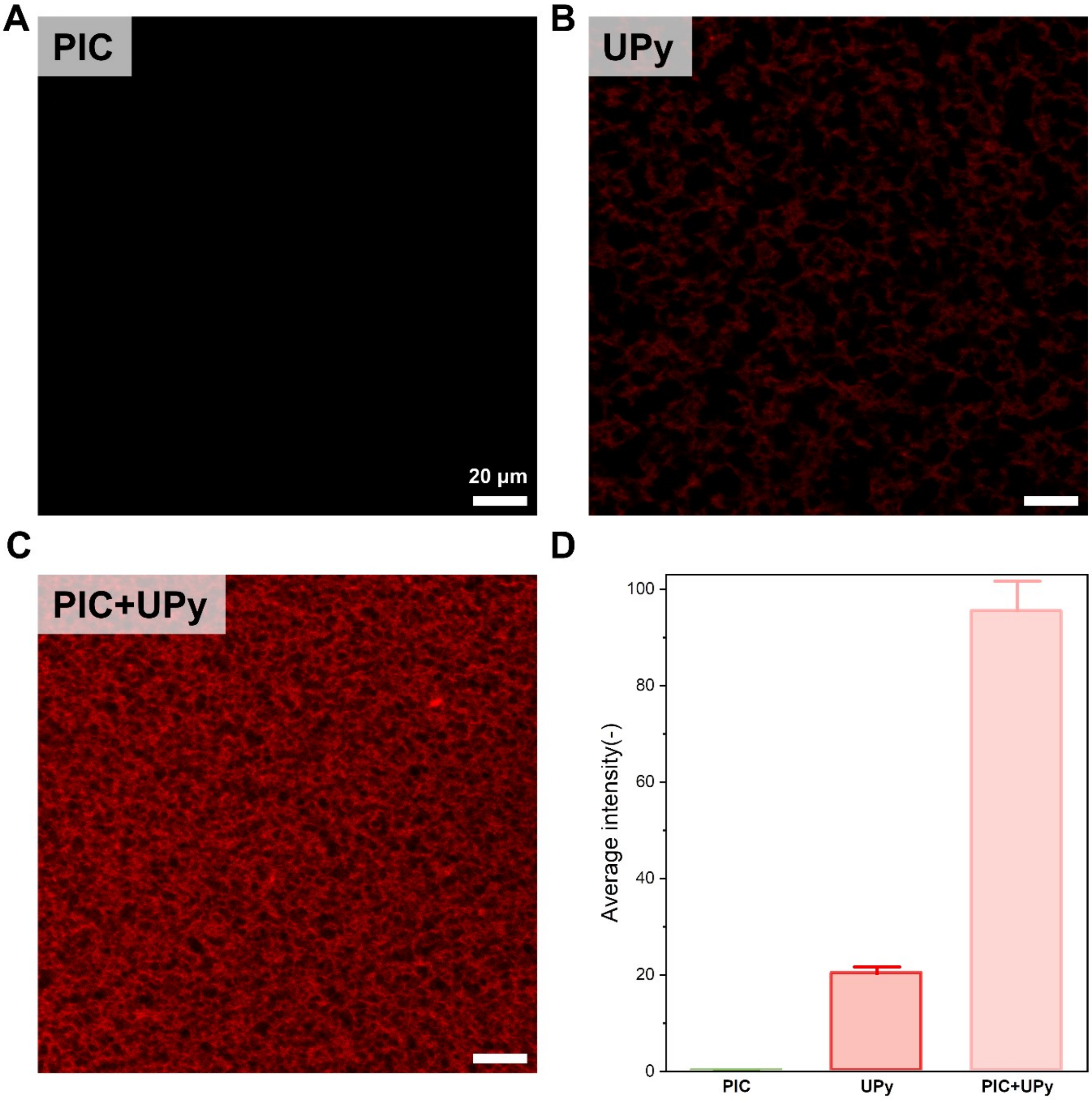

Fig. S29. FRET experiments showing PIC and UPy are in very close proximity. PIC (0.05 w/v%), functionalized with a TAMRA dye (15 µM), UPy (0.24 w/v%) mixed with UPy-Cy5 (15 µM) and the hybrid mixture PIC+UPy. For alle samples, excitation occurred at 552 nm with identical laser power (excitation of TAMRA) and emission was collected at 700 – 800 nm. **(A)** No signal is observed for the PIC hydrogels, as emission of TAMRA occurs before 700 nm. **(B)** Small background signal is observed from the UPy hydrogel. **(C)** Clear mesh-like network is observed for the PIC+UPy mixture, indicating FRET signal from the TAMRA dye to the UPy-Cy5. **(D)** Quantification of the average intensities obtained from the separate and the hybrid mixtures. Scale bar = 20 µm.

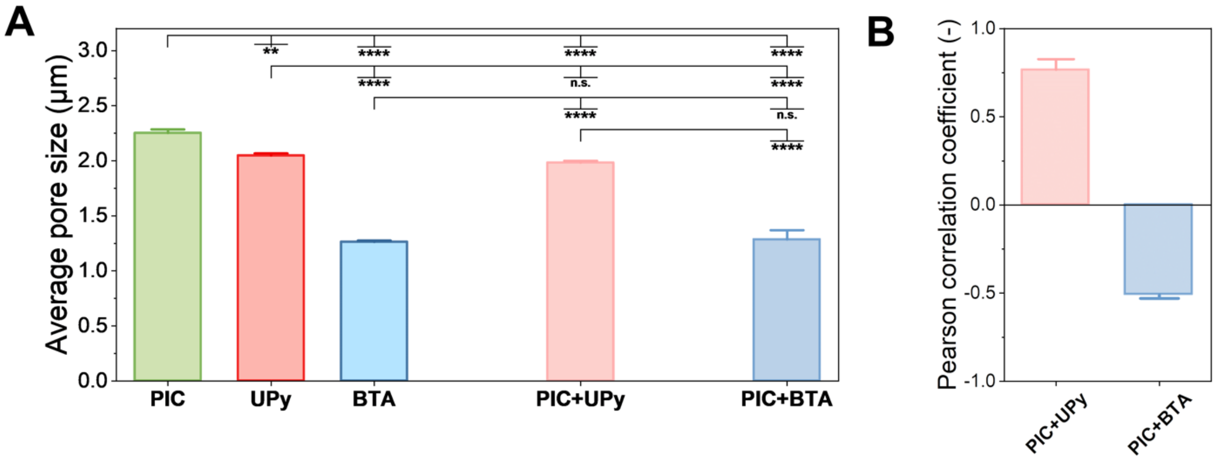

Fig. S30. Quantification of structural properties of the hybrid networks. (A) Pore size quantification of the single and hybrid networks, showed in Fig. S17 and S28. Indicating that upon mixing UPy and BTA with the PIC, the PIC network looks dominant. The pore size of UPy and BTA increases to better match the pores of the PIC. **(B)** Pearson correlation coefficient to quantify the overlap in the hybrid networks, comparing PIC channel to either UPy channel or BTA channel. PIC+UPy forms a single network with high positive overlap. In contrast, PIC+BTA, forms a two-component network with negative overlap, indicating BTA forms in the PIC pores. Bar graph represents mean with SEM. ** $P < 0.05$; **** $P < 0.0001$. Analysis by one-way ANOVA.

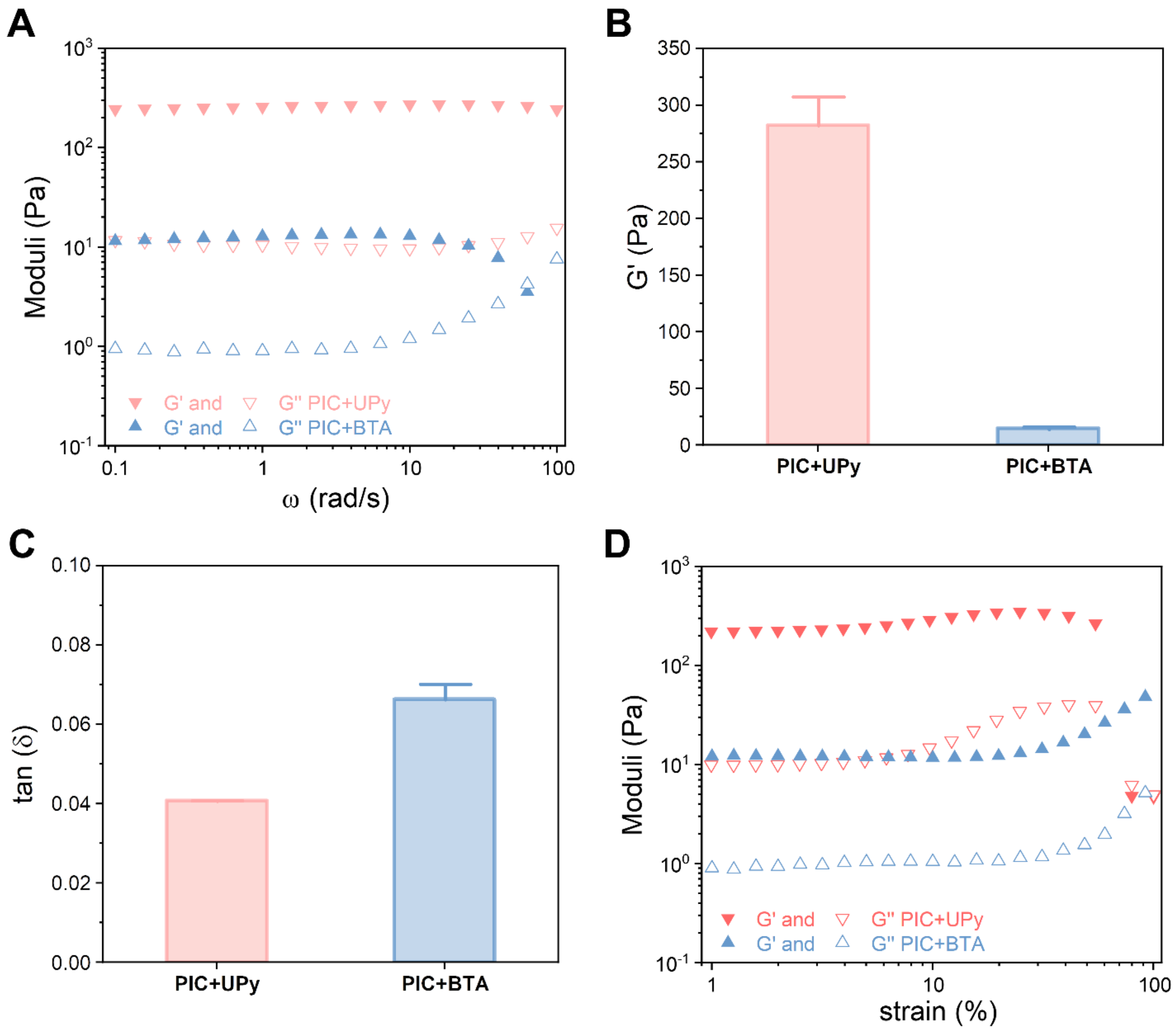

Fig. S31. Mechanical characterization at 37 °C of the hybrid networks. (A) Frequency response at y = 1%, showing elastic-like networks at the measured time scales. **(B)** Quantification of G’ at 1 rad/s and 1% strain, showing the formation of a very stiff PIC+UPy network. **(C)** Quantification of tan δ, showing that PIC+UPy forms a more elastic like network than PIC+BTA. **(D)** Strain sweep at 1 rad/s, similar as the stress-stiffening experiments, a stronger strain stiffening response is observed for PIC+BTA compared to the PIC+UPy.

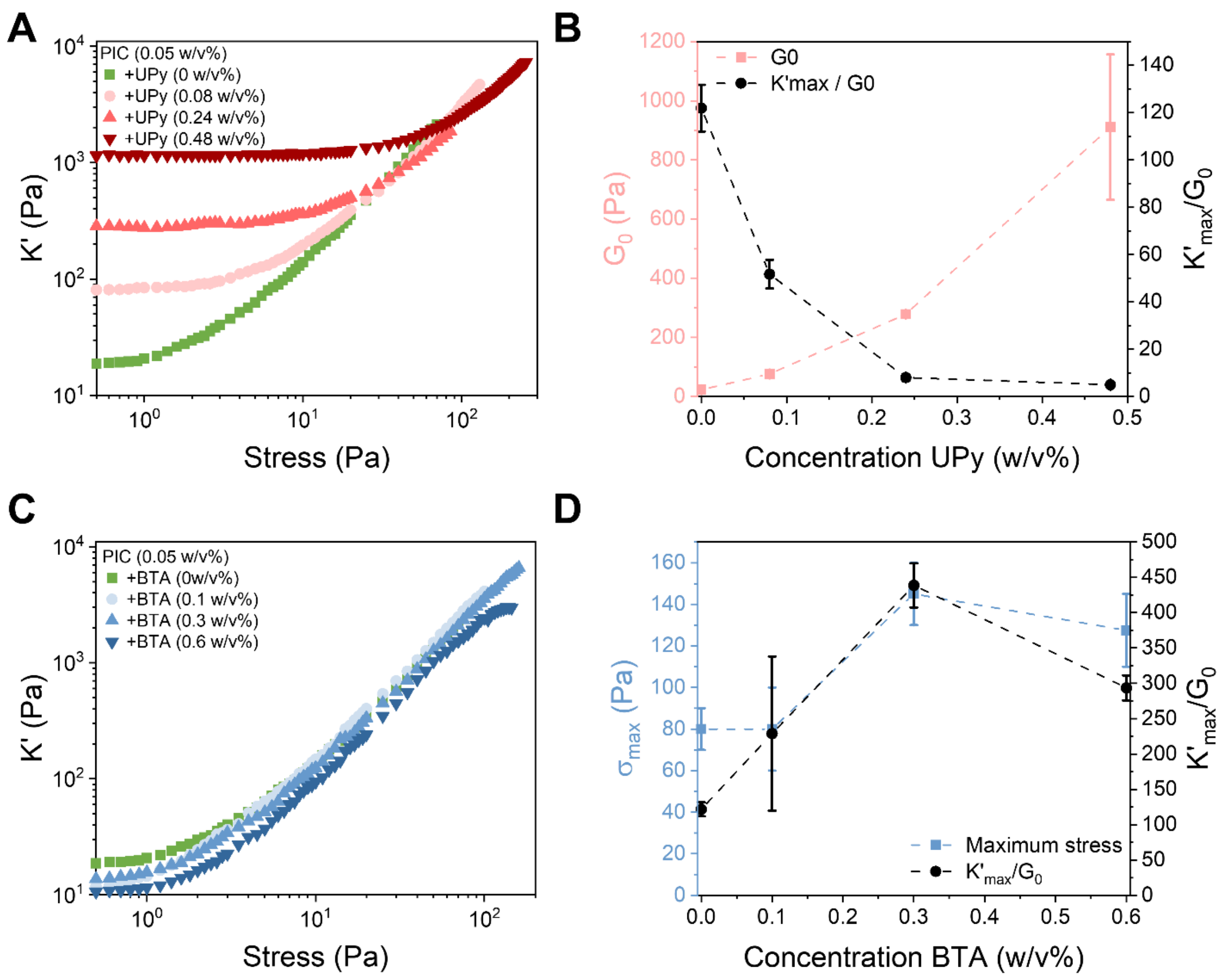

Fig. S32. Tunability of the stress-stiffening behavior through varying the UPy concentration in PIC+UPy networks or the BTA concentration in PIC+BTA networks. (A) The differential modulus (Pa) vs stress with varying UPy concentration. **(B)** Quantification of the plateau modulus, G_0 (Pa), as well as the stiffness increase, K'_{max}/G_0, plotted against the UPy concentration. With increased UPy concentration, the plateau modulus increases but the stress stiffening response weakens. **(C)** The differential modulus (Pa) vs stress with varying BTA concentration. **(D)** Quantification of the maximum stress (Pa), as well as the stiffness increase, K'_{max}/G_0, plotted against the BTA concentration. There is a clear optimum concentration at which BTA enhances the maximum stress and stress stiffening response.

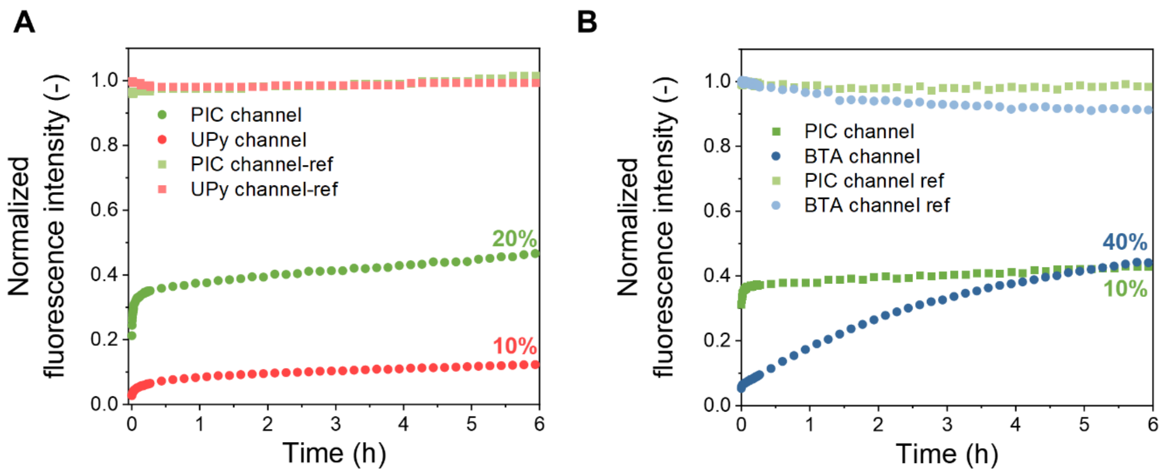

Fig. S33. Dynamic behavior at 37 °C of the hybrid networks. (A) Dynamic behavior followed via FRAP measurements, following the recovery for 6 h of the PIC+UPy networks **(B)** and of the PIC+BTA networks.

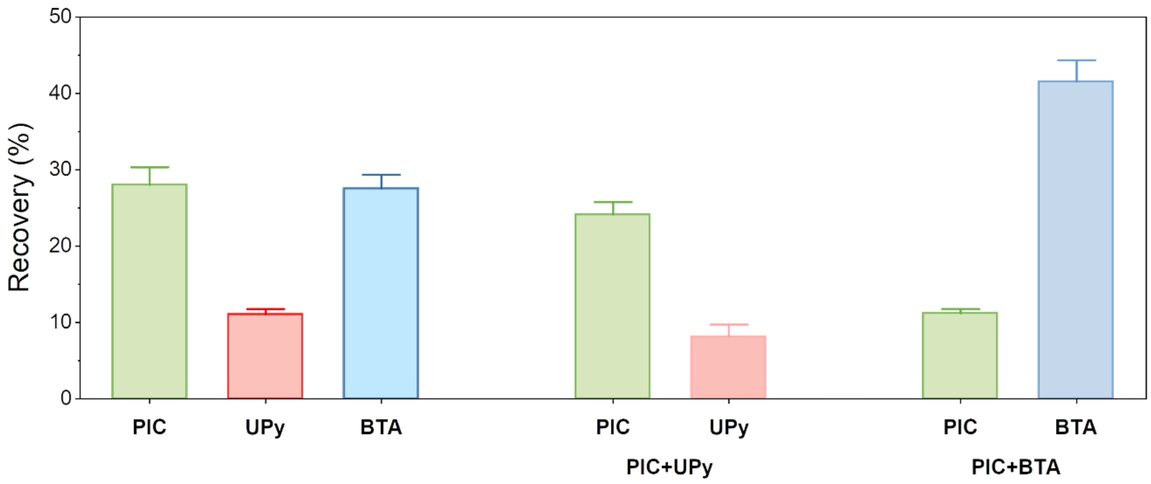

Fig. S34. Quantification of FRAP recovery after 6 h, showing the dynamic properties of the single components and the hybrid networks at the local scale (50 µm). UPy forms a more rigid network than the BTA, which is translated to the hybrid networks.

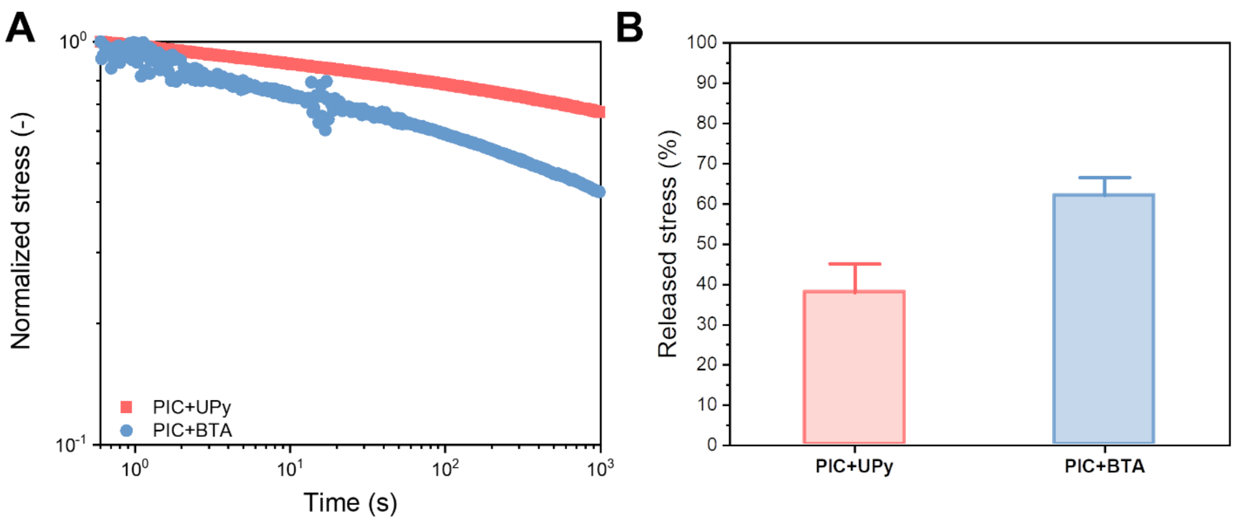

Fig. S35. Dynamic behavior at 37 °C of the hybrid networks: PIC+UPy (0.05 w/v% + 0.24 w/v%) and PIC+BTA (0.05 w/v% + 0.30 w/v%). (A) Stress relaxation measurements with 7.5% strain. Indicating that PIC+BTA forms a more dynamic network compared to the PIC+UPy. **(B)** Quantification of relaxed stress after 1000 seconds.

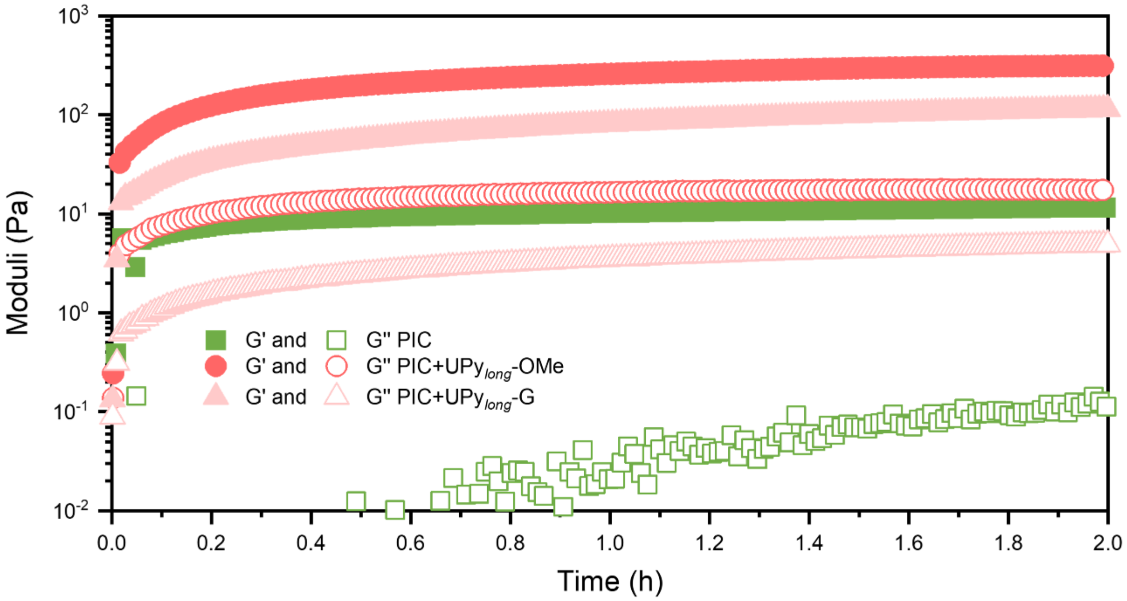

Fig. S36. Formation of two-component networks PIC+UPy with changing UPy-end group. PIC+UPy$_{long}$-OMe (0.05 w/v% + 0.24 w/v%) and PIC+UPy$_{long}$-G (0.05 w/v% + 0.28 w/v%) with both UPy's in identical molar concentrations. UPy$_{long}$-G is a slightly more dynamic fiber compared to UPy-OMe, but still an increased bulk stiffness is observed, although less as compared to the more rigid UPy-OMe fibers. Measured at 1% strain and 1 rad/s at 37 °C.

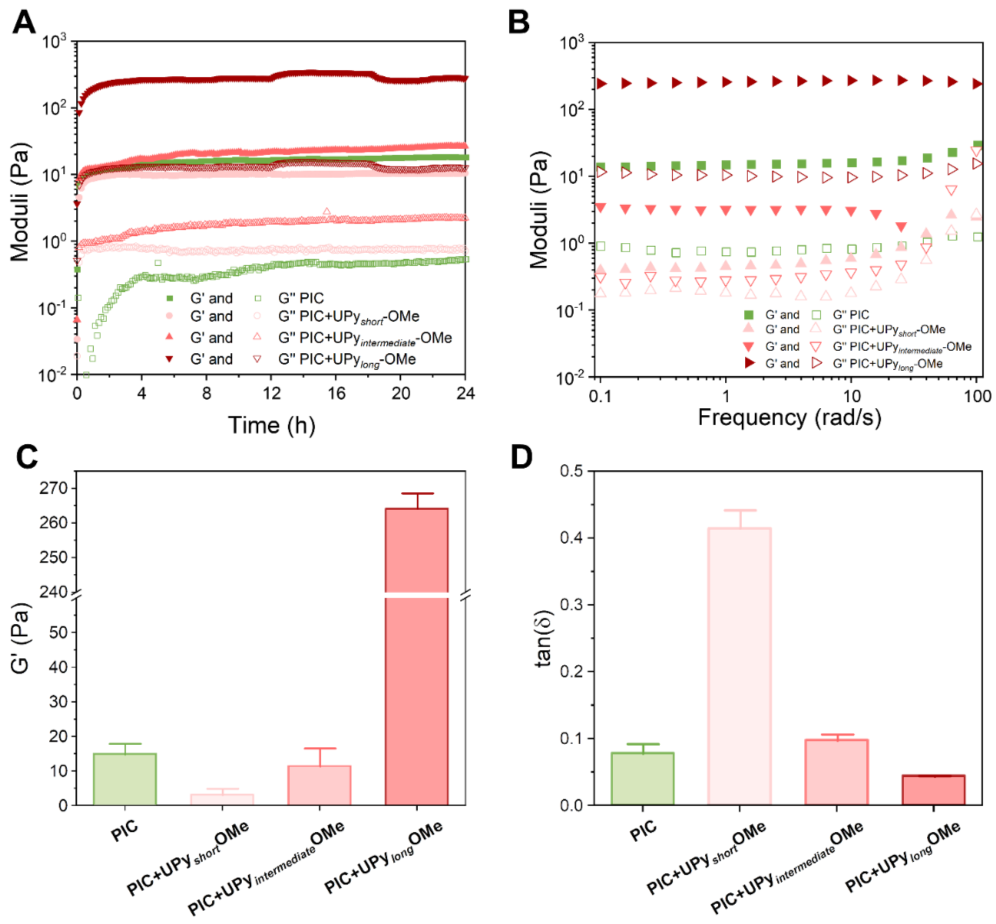

Fig. S37. Mechanical and dynamic characterization of two-component networks of PIC mixed with more dynamic UPys. Molar concentration of UPy is kept constant, resulting in: PIC+UPy$_{long}$-OMe (0.05 w/v% + 0.24 w/v%), PIC+UPy$_{intermediate}$-OMe (0.05 w/v% + 0.20 w/v%), PIC+UPy$_{long}$-OMe (0.05 w/v% + 0.16 w/v%). **(A)** Formation of the various hybrid networks followed over time at 37 °C at ω =1 rad/s and y = 1%. **(B)** Frequency response, showing all stable elastic-like networks with a large variation in the absolute values. **(C)** Quantification of the stiffness at 1% strain and 1 rad/s, showing that only the most stable UPy increases the bulk stiffness. **(D)** Visco-elastic behavior quantified at 1% strain and 1 rad/s, showing that more dynamic UPy molecules increase the viscous behavior.

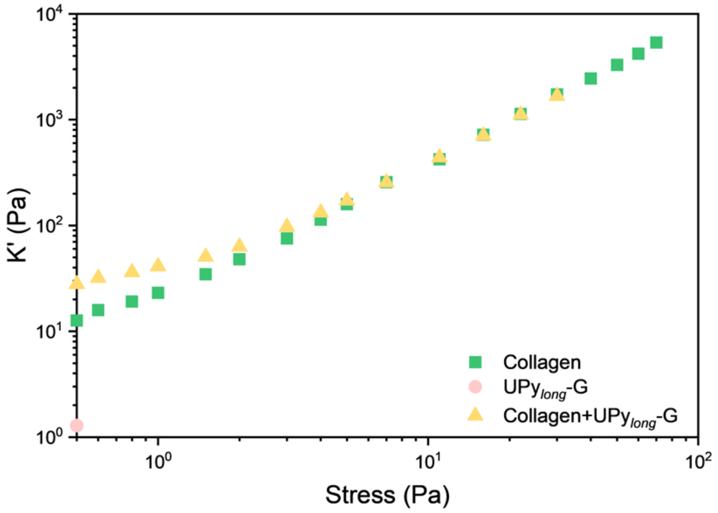

Fig. S38. Stress stiffening response using a natural polymer instead of synthetic PIC, showing no synergistic effects between collagen and UPy. Concentration of collagen (0.25 w/v%) is chosen to match the stiffness of PIC. Concentration of UPy$_{long}$-G (0.28 w/v%) is similar as used in the other PIC hybrid mixtures.

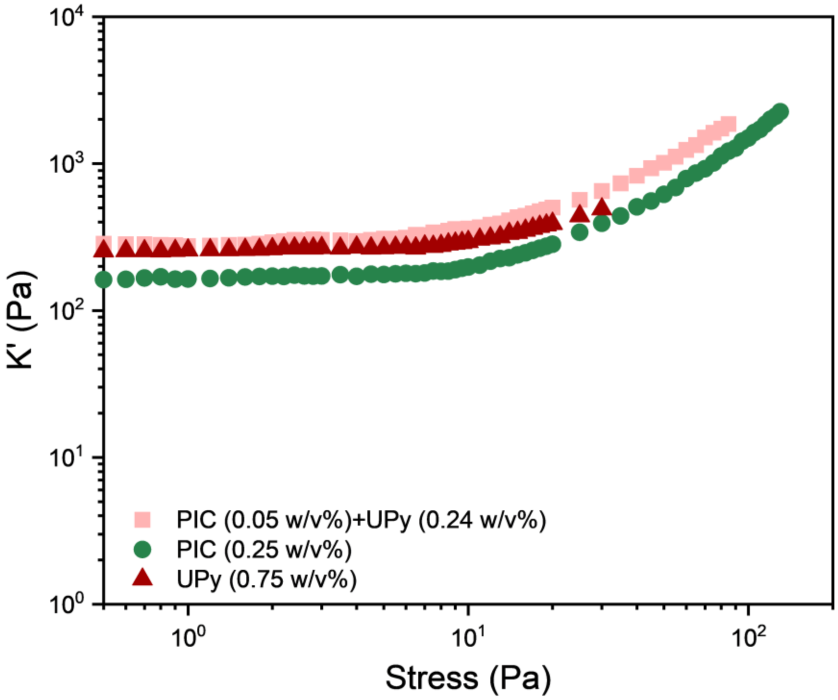

Fig. S39. Stress stiffening response of PIC+UPy compared to the single PIC and UPy networks at higher concentrations, to compare the possibility to use single networks with similar stiffness. Higher concentrations of the single networks are needed to reach a similar plateau modulus as compared to PIC+UPy.

8. Control two-component networks using another dynamic, bis-urea supramolecular monomer (OBO)

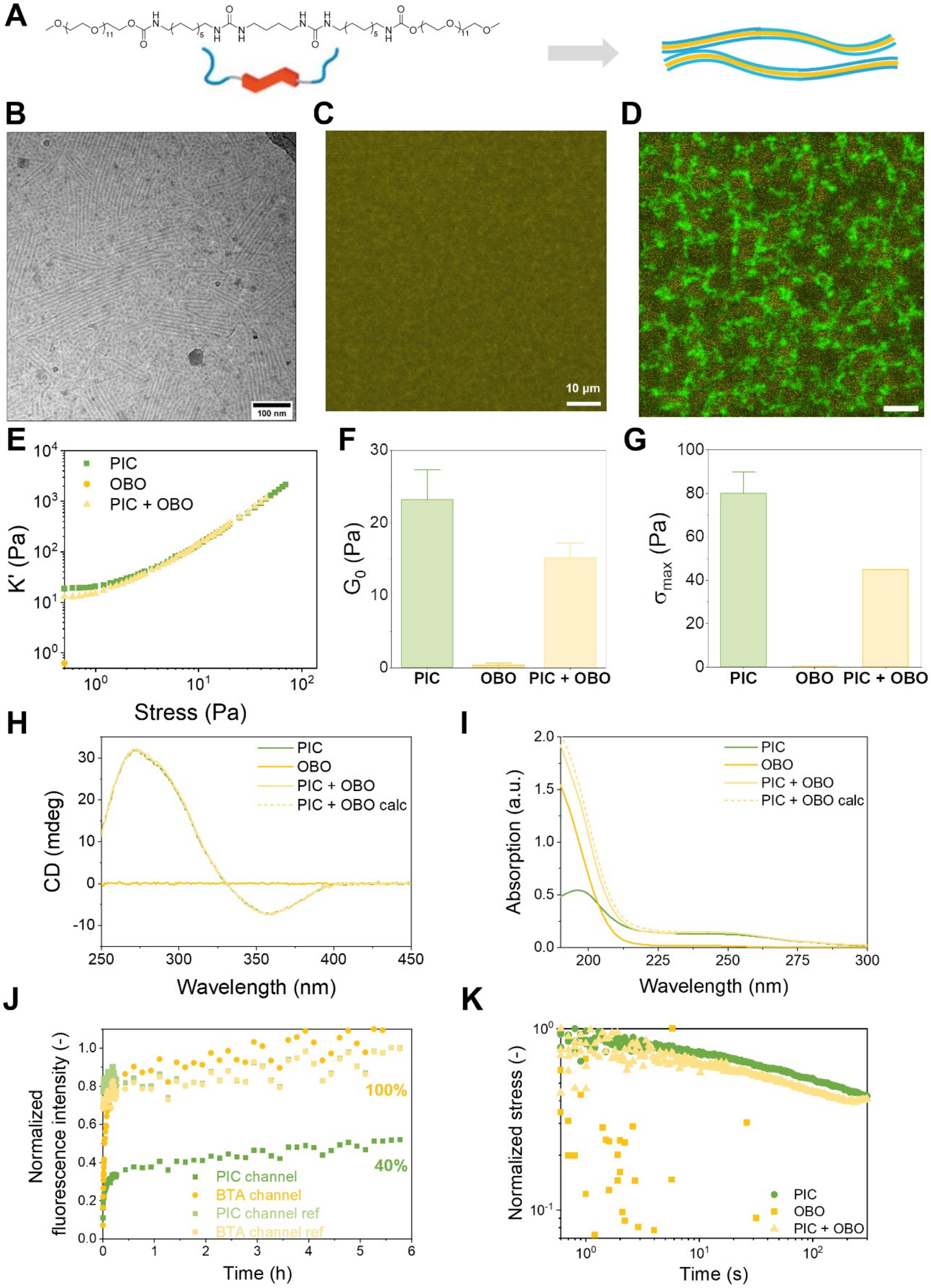

Fig. S40. Usage of bis-urea supramolecular building block as control for combination of PIC with supramolecular fibers: PIC+OBO (0.05 w/v% + 0.40 w/v%) (A) Chemical structure of

OBO and its assembly in bidirectional structures. **(B)** Cryo-TEM image showing a thick block-like architecture, sample is 5 times diluted. **(C)** Confocal image showing complete liquid-like structure. **(D)** Confocal image of the PIC (green) and OBO (yellow), showing no overlap. Scale-bar = 10 μm. **(E)** Stress stiffening of PIC+OBO and the separate components **(F)** showing no enhanced properties in terms of plateau modulus **(G)** and maximum stress. **(H)** CD measurement and **(I)** UV-vis measurement showing that addition of OBO has no effect on the PIC structure or yields synergistic properties. **(J)** Dynamic behavior determined via FRAP **(K)** and stress relaxation. Indicating OBO is too dynamic to induce any synergistic response.

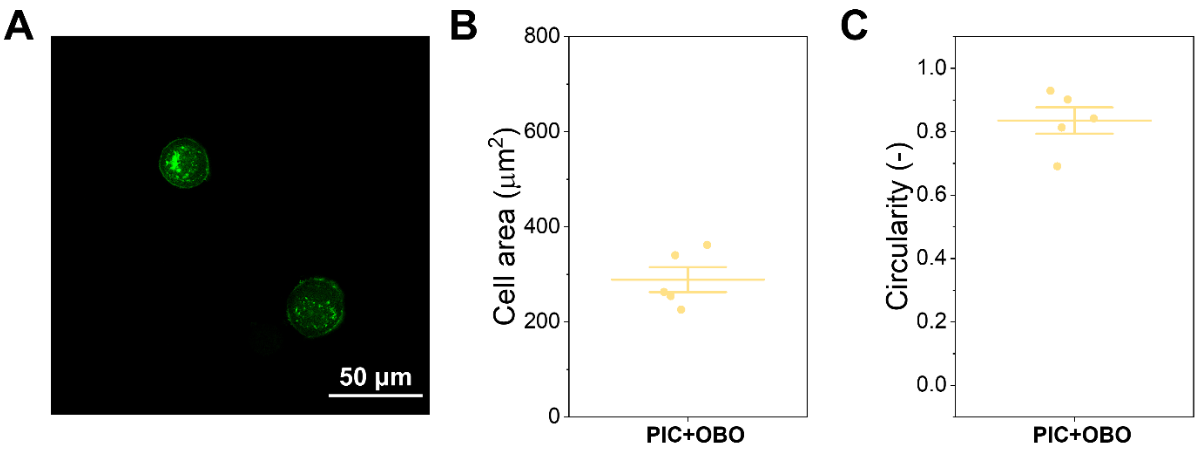

Fig. S41. 2D cell experiments of hybrid networks composed of PIC mixed with bis-urea supramolecular monomer (OBO), combined with 0.5 mM OBO-cRGD. (A) Representative image of cells cultured on PIC with OBO and OBO-cRGD showing no cell spreading. In green is F-actin**.** Scale bar = 50 µm. **(B)** Quantification of cell area in µm^2 and **(C)** circularity. Data points represent individual cells, plotted with mean and SEM.

9. Cellular experiments with the two-component networks

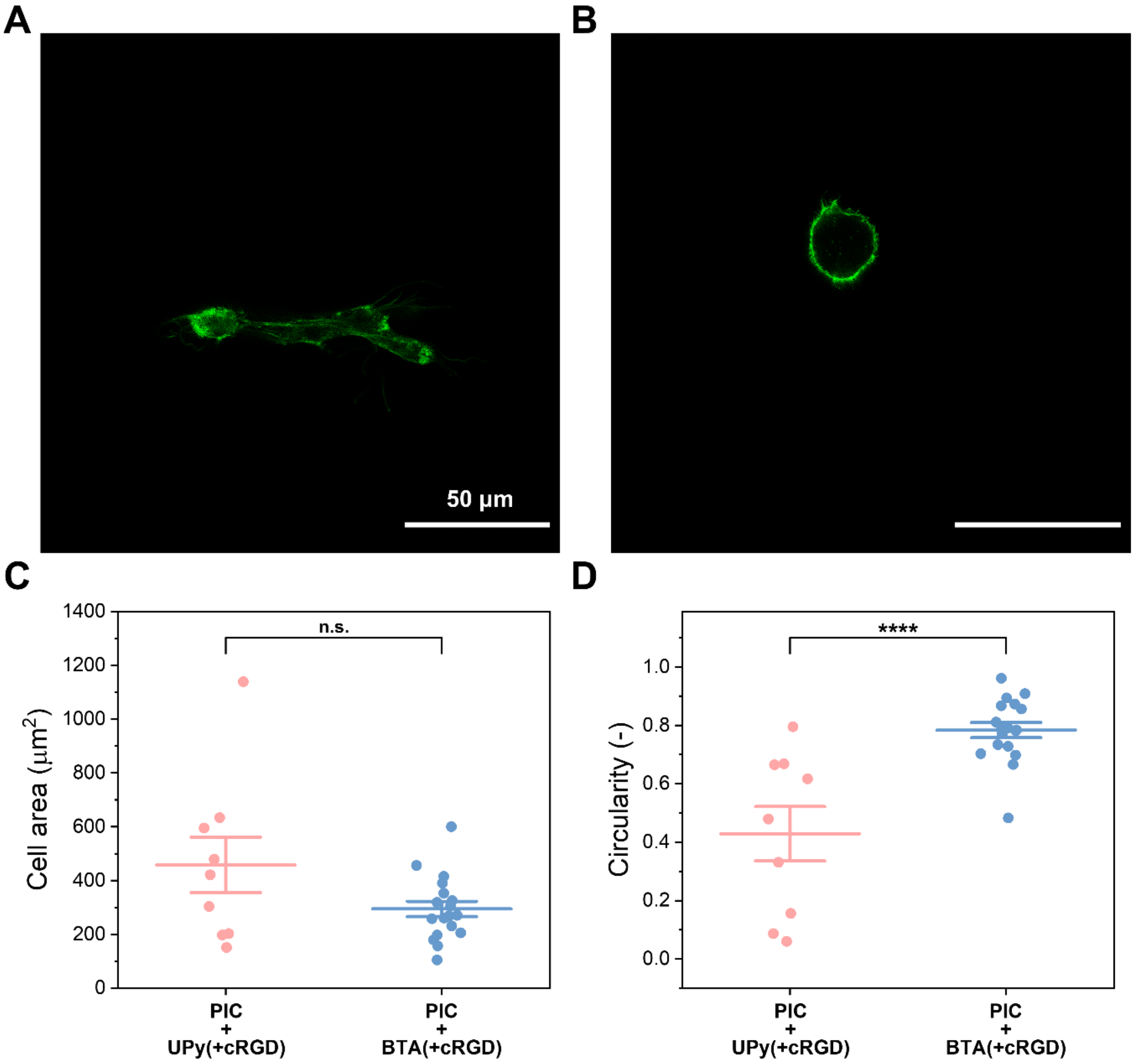

Fig. S42. 2D cell experiment, where cells are cultured on top of the two hybrid networks, showing only cell spreading on the PIC+UPy gels. (A) Representative image of cells on top of **(A)** PIC+UPy and **(B)** PIC+BTA. For cell-adhesion, UPy-cRGD or BTA-cRGD (0.5 mM) was included. Scale bar = 50 µm. **(C)** Quantification of cell area µm^2 and **(D)** circularity, showing only spread cells when cultured on PIC+UPy**.** Data points represent individual cells, plotted with mean and SEM.**** P < 0.0001, by unpaired t-test.

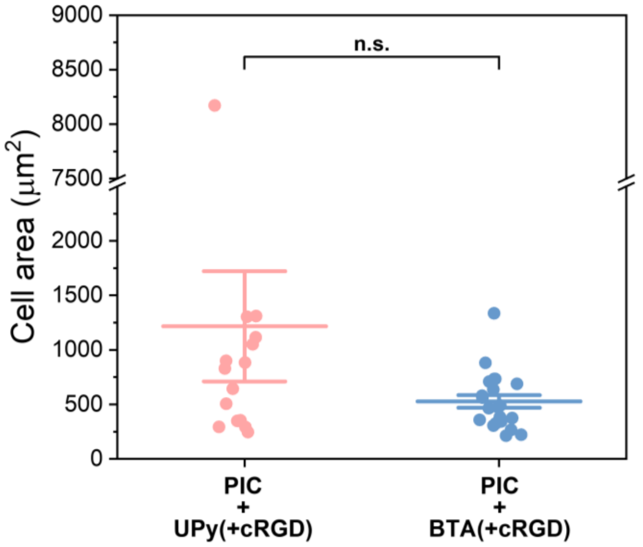

Fig. S43. Quantification of cell area in 3D for the hybrid networks. Data points represent individual cells, plotted with mean and SEM. Analysis by Mann-Whitney test.

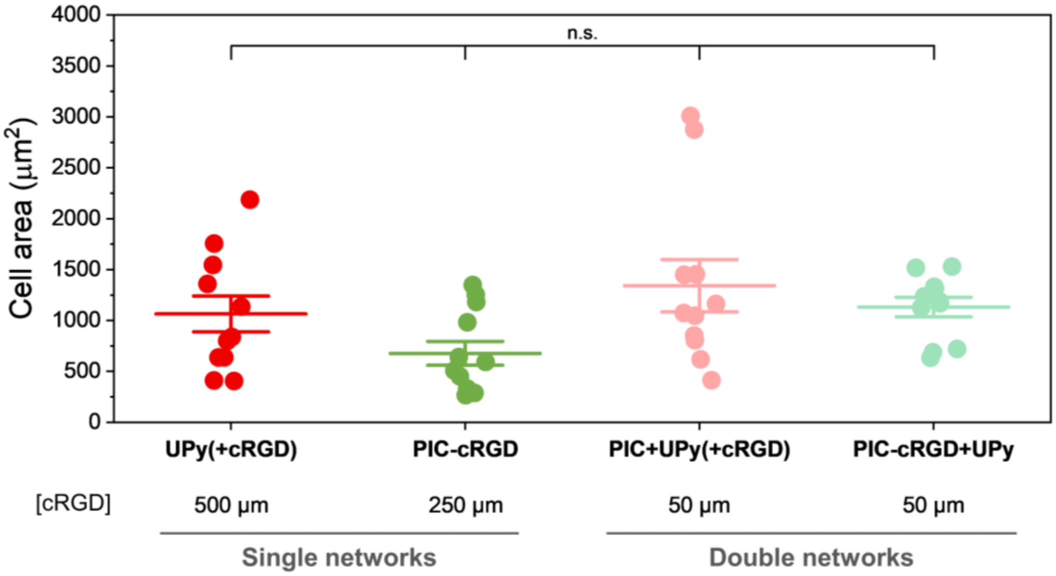

Fig. S44. Quantification of cell area in 3D for the hybrid networks. Single networks contained a higher concentration of PIC or UPy to match the plateau modulus of the PIC+UPy hydrogel. Data points represent individual cells, plotted with mean and SEM. Analysis by one-way ANOVA.

10. Control two-component networks based on UPy and BTA, showing no synergistic effects

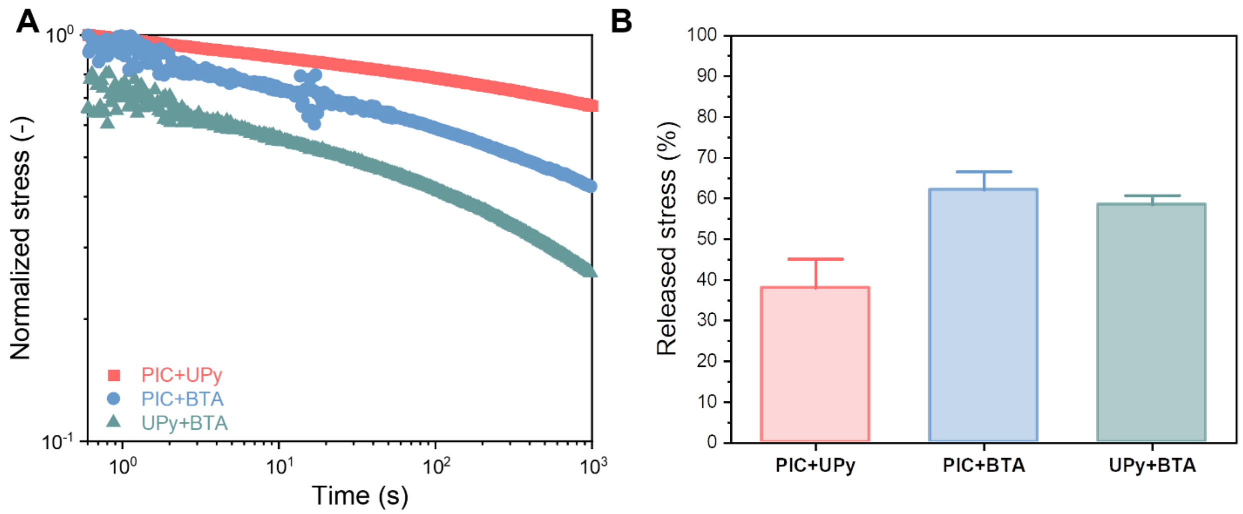

Fig. S45. Dynamic behavior at 37 °C of various two-component networks. Showing that incorporation of BTA is needed to enhance the stress relaxation.

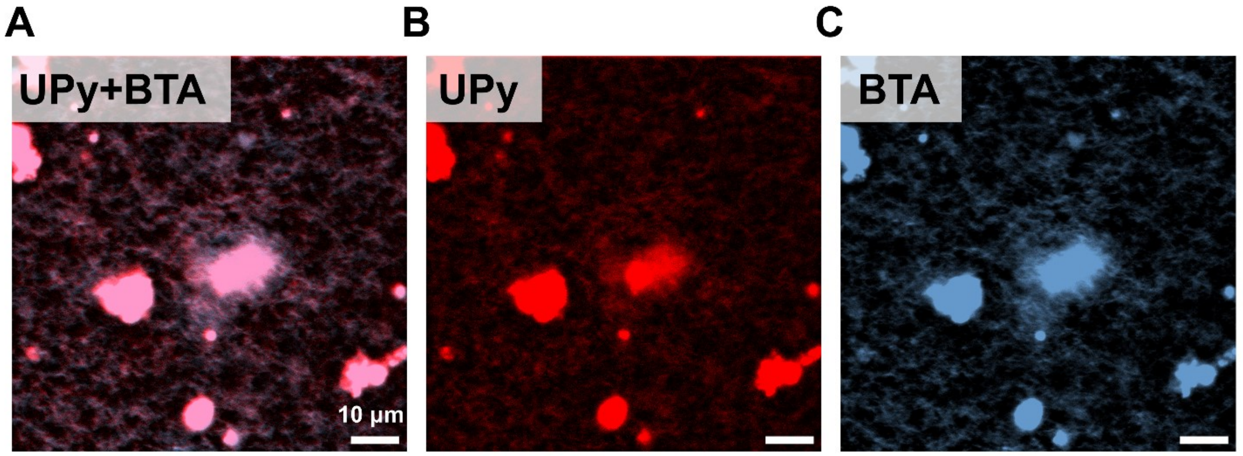

Fig. S46. Investigation of structural overlap in UPy and BTA combined networks. UPy (0.24 w/v%) is mixed with UPy-carboxyfluorescein (UPy-FITC) (15 µM) and combined with BTA (0.3 w/v%) and BTA-Cy5 (15 µM). (A) Merged channel, showing regions of overlap between the two components, although also high amounts of clustering occurred. **(B)** Signal of the UPy channel consisting of UPy mixed with UPy-FITC. **(C)** Signal of the BTA channel, consisting of BTA mixed with BTA-Cy5**.** Scale bar = 10 µm

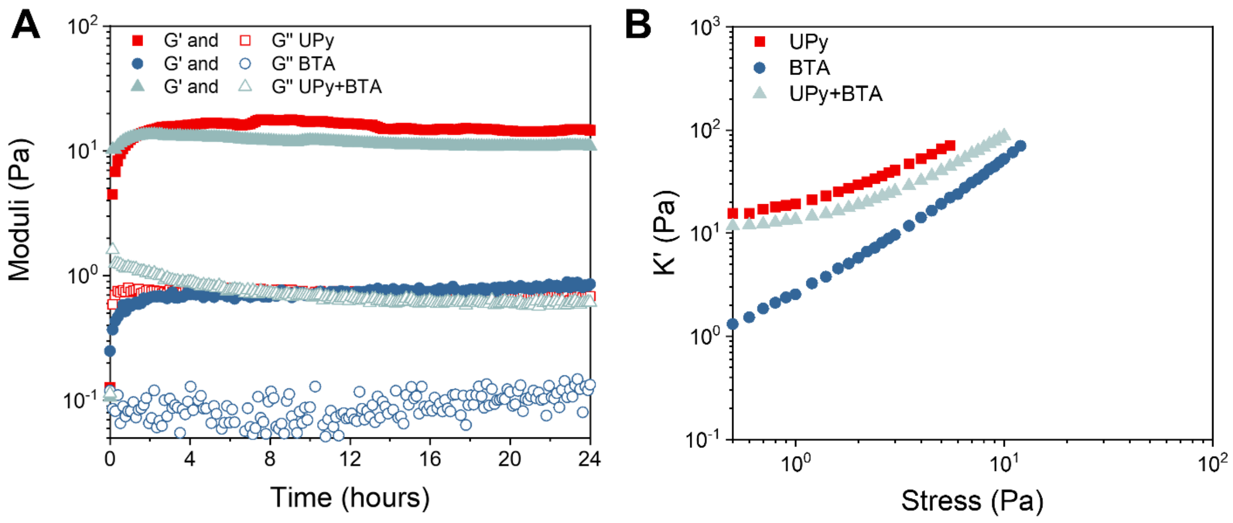

Fig. S47. No synergistic mechanical effects of UPy (0.24 w/v%), combined with BTA (0.30 w/v%), without PIC being present (A) Mixing UPy and BTA showed no synergistic effects in the formation, which is mainly dominated by the UPy network, which is much stiffer than the BTA network. Formation was measured at 37 °C at $\omega = 1$ rad/s and $\gamma = 1\%$ strain. **(B)** Stress-stiffening measurement, showing an intermediate response for the UPy+BTA, with a plateau modulus slightly below UPy and with the stress resistance of the BTA.

11. Characterization of three-component networks in hydrogel state

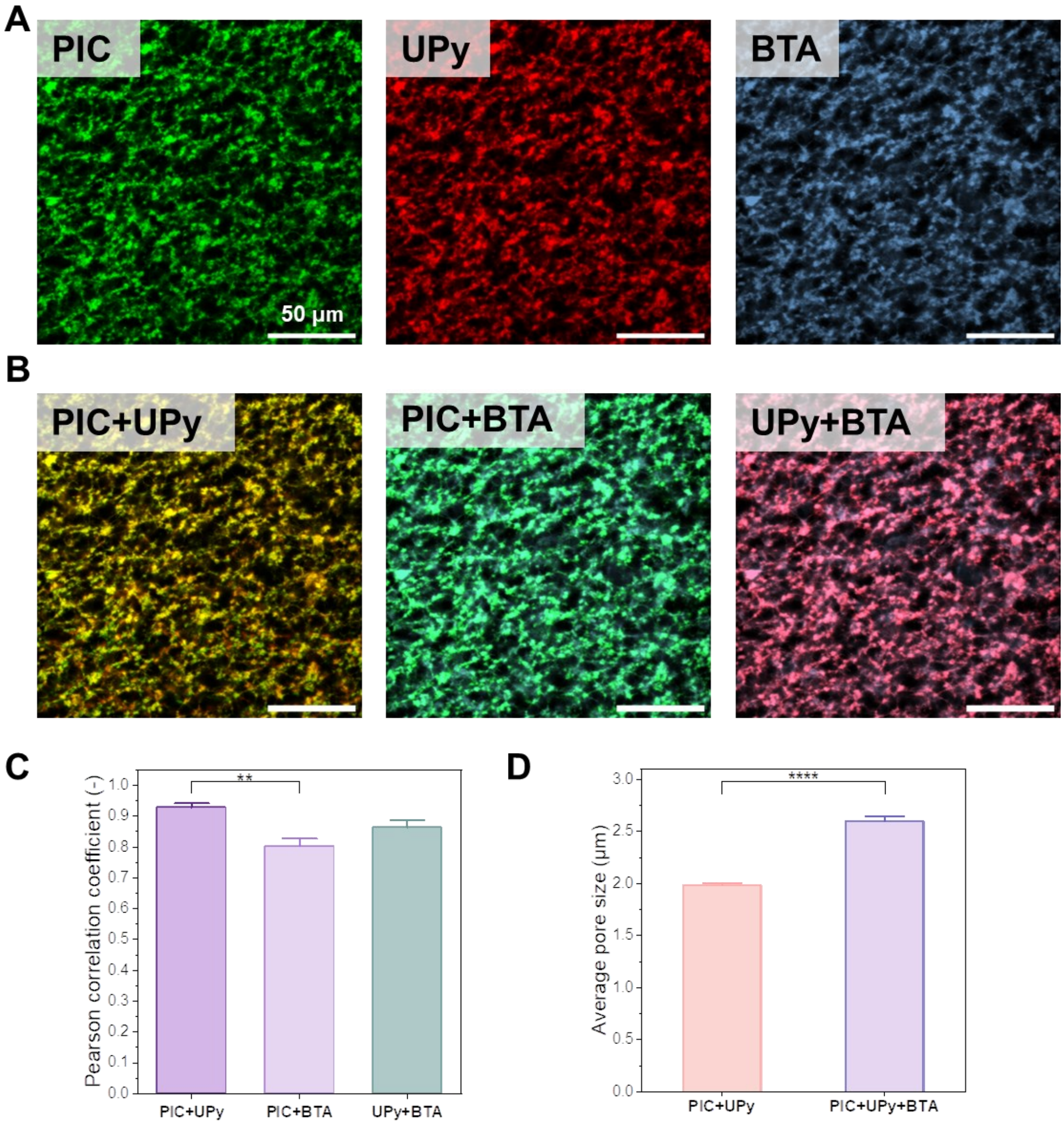

Fig. S48. Confocal images of the PIC+UPy+BTA network. (A) Single channels showing the separate networks. **(B)** Merged channels showing the overlap between PIC (green)**,** UPy (red) and BTA (blue). Scale bar = 50 µm. **(C)** Pearson correlation coefficient to quantify the overlap between the networks by comparing different channels in the three component network. ** $P < 0.01$, by one way ANOVA. **(D)** Average pore size of the three component network in comparison to the two component network, showing larger pores in the three component network. **** $P < 0.0001$, by unpaired t-test.

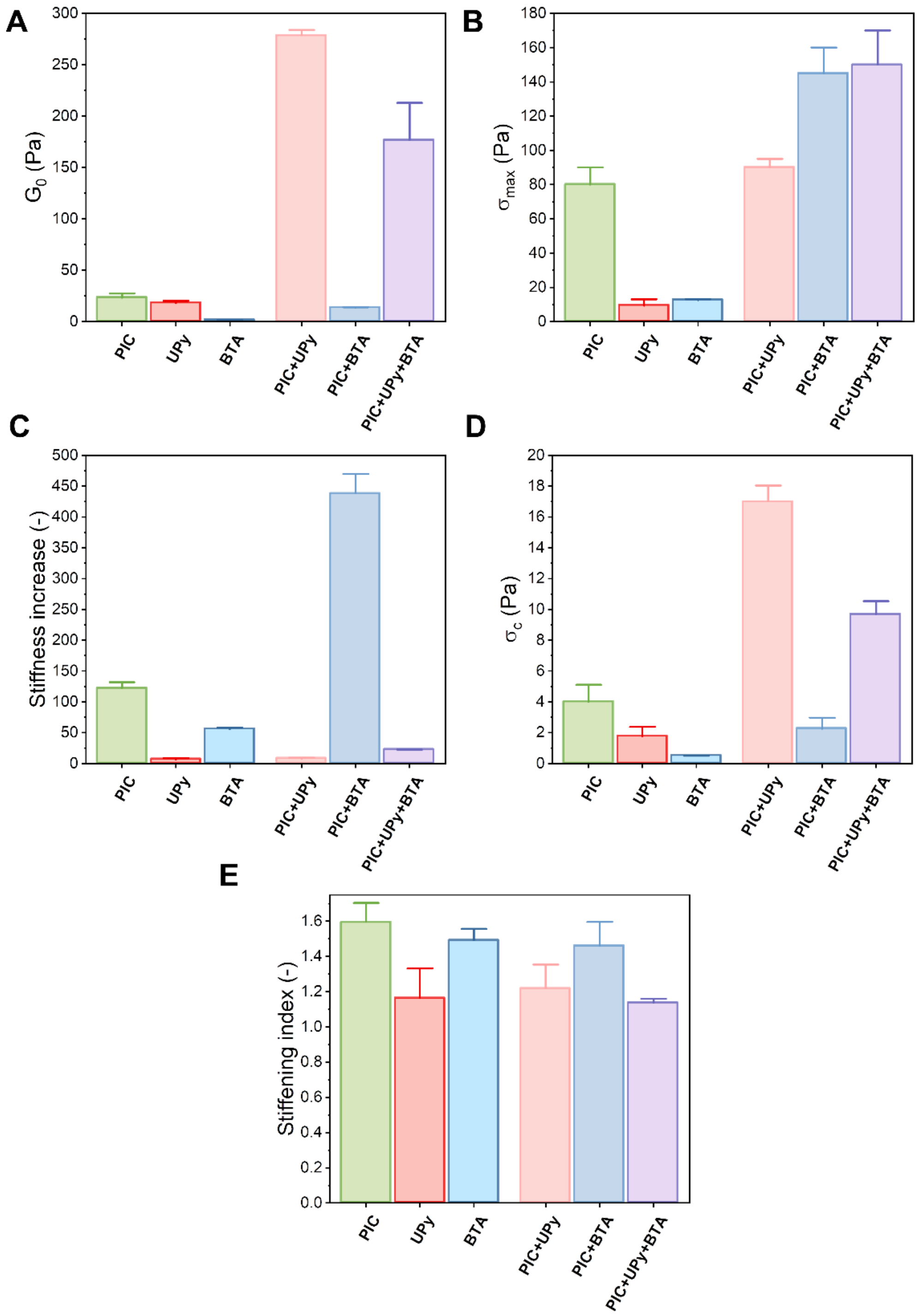

Fig. S49. Stress stiffening quantification of the single, hybrid and three-component networks: PIC (0.05 w/v%), UPy (0.24 w/v%) and BTA (0.30 w/v%). (A) Plateau modulus extracted from the stress-stiffening response. A large increase is observed when UPy is added to the PIC. In the three-component network, this increase in stiffness is still present. **(B)** Maximum stress, measure of the stress resistance. When BTA is present with PIC, almost twice as much stress can be put on the network before rupture. **(C)** Stiffness increase, which is quantified by comparing the maximum obtained stiffness before rupture to the plateau modulus, showing a very large increase for

PIC+BTA. The increase is lower for PIC+UPy+BTA, due to the already higher plateau modulus. **(D)** Critical stress, a measure for the sensitivity, i.e. the start of the stress-stiffening response. The three-component network has increased sensitivity compared to the PIC+UPy network. **(E)** Stiffening index, a measure for the responsiveness of the stress stiffening. Which is slightly reduced when UPy is added to the PIC network.

12. Cellular experiments using the three-component networks.

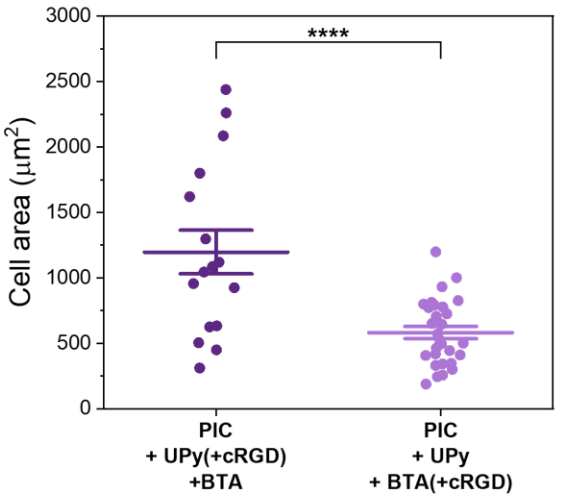

Fig. S50. Comparison of cell area of 3D cell culture between PIC+UPy(+cRGD)+BTA and PIC+UPy+BTA(+cRGD). Data points represent individual cells, plotted with mean and SEM. **** $P < 0.0001$, by unpaired t-test.

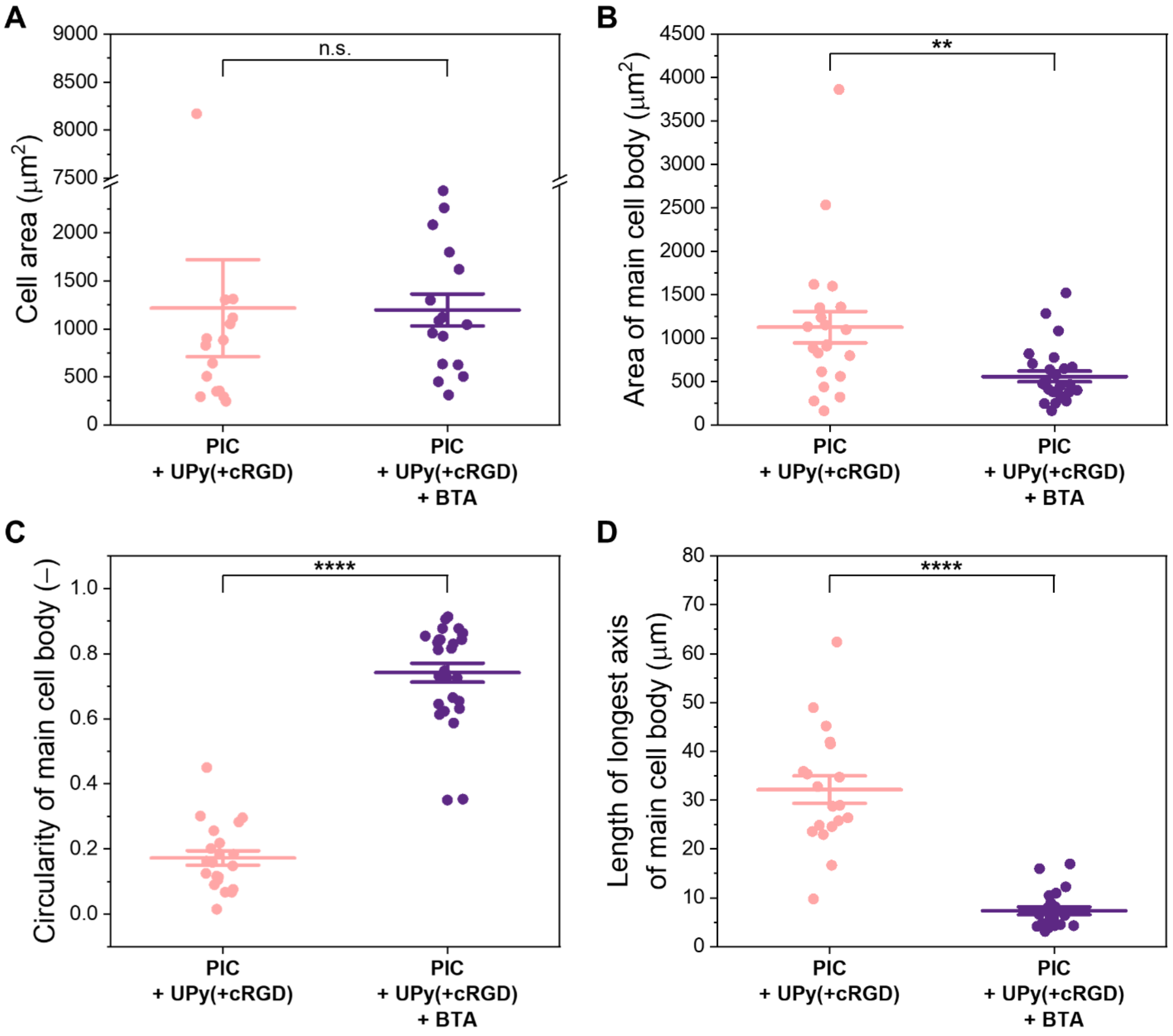

Fig. S51. Comparison of 3D cell culture between PIC+UPy(+cRGD) and PIC+UPy(+cRGD)+BTA. (A) Comparison of cell area, **(B)** main cell body area, **(C)** main cell body circularity and **(D)** length of longest axis. Data points represent individual cells, plotted with mean and SEM. ** $P < 0.01$; **** $P < 0.0001$, by Mann-Whitney test.

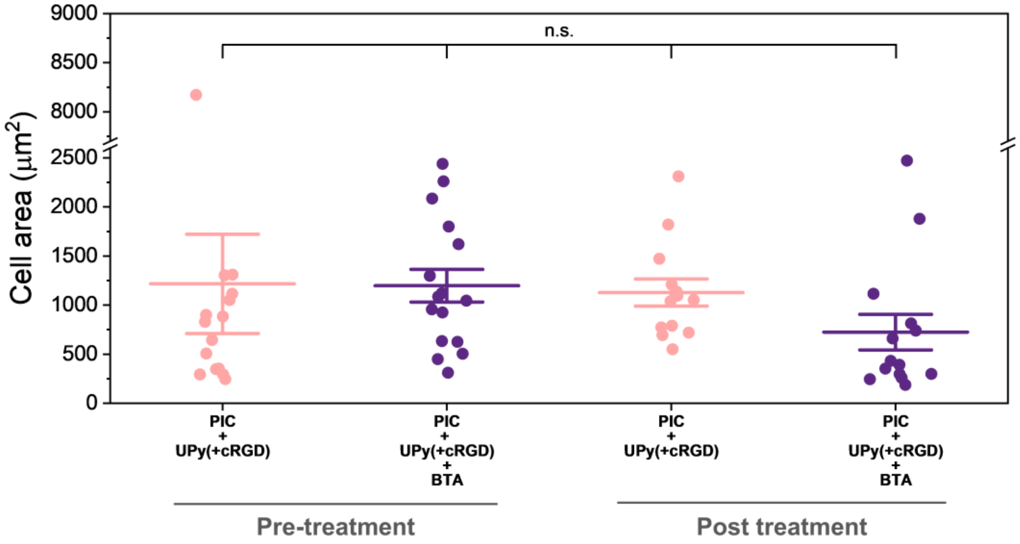

Fig. S52. Quantification cell area before and after ROCK inhibitor on the two-component PIC+UPy hydrogel and three-component PIC+UPy(+cRGD)+BTA hydrogel. Data points represent individual cells, plotted with mean and SEM. Analysis by Kruskal-Wallis.

13. Control three-component network using the more dynamic, bis-urea supramolecular monomer: PIC+UPy+OBO

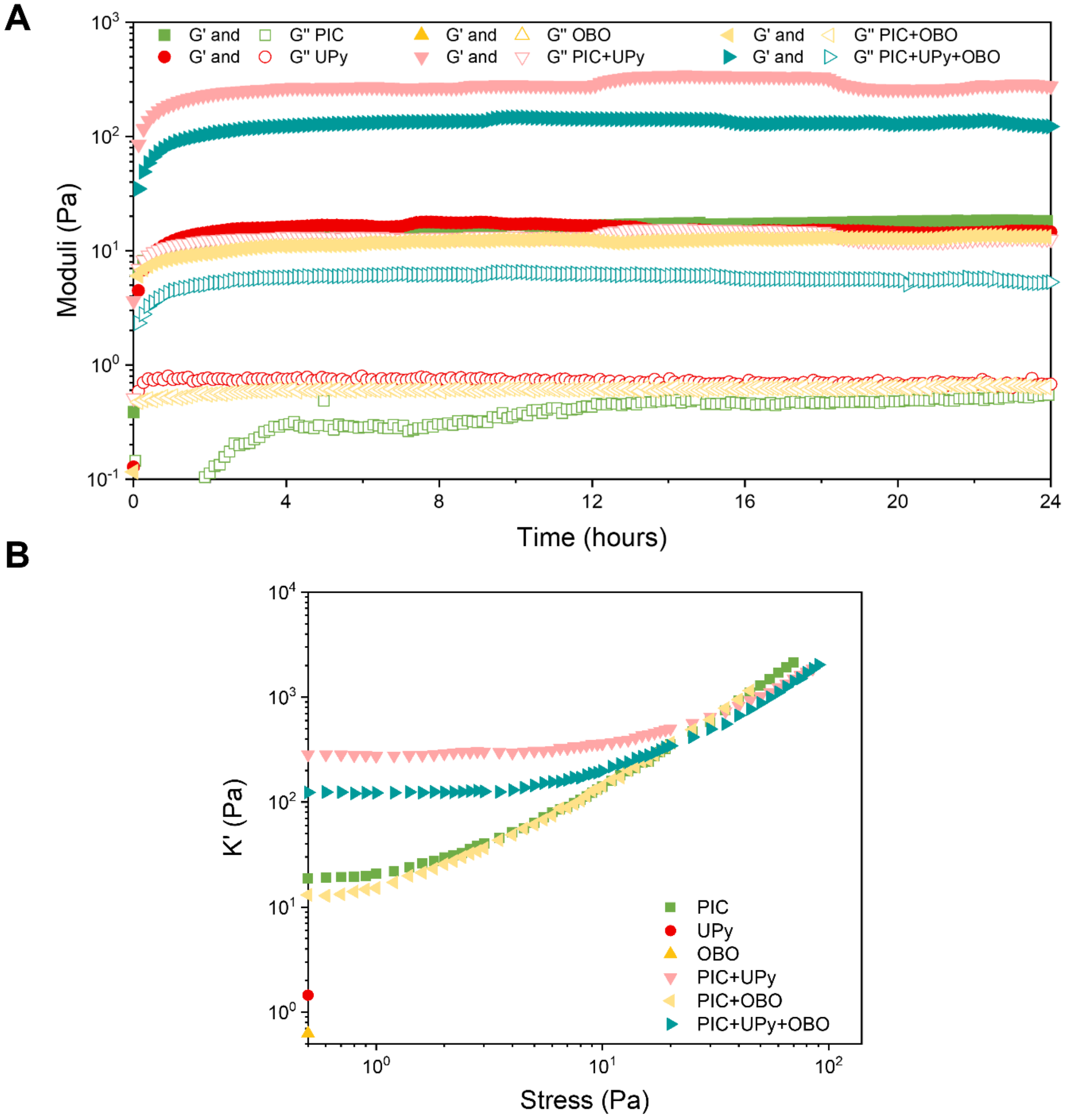

Fig. S53. Three component network composed of PIC (0.05 w/v%), BTA (0.30 w/v%) and OBO (0.40 w/v%), instead of BTA, showing that BTA cannot be replaced by another supramolecular fiber, as OBO lowered the plateau modulus. (A) Network formation of the single, hybrid and three-component network. **(B)** Stress stiffening response of single hybrid and three component networks. When OBO is added to PIC + UPy, the plateau modulus becomes lower.

REFERENCES AND NOTES

1. K. J. Lampe, A. L. Antaris, S. C. Heilshorn, Design of three-dimensional engineered protein hydrogels for tailored control of neurite growth. *Acta Biomater.* **9**, 5590–5599 (2013).

2. O. Chaudhuri, L. Gu, M. Darnell, D. Klumpers, S. A. Bencherif, J. C. Weaver, N. Huebsch, D. J. Mooney, Substrate stress relaxation regulates cell spreading. *Nat. Commun.* **6**, 6365 (2015).

3. Z. Álvarez, A. N. Kolberg-Edelbrock, I. R. Sasselli, J. A. Ortega, R. Qiu, Z. Syrgiannis, P. A. Mirau, F. Chen, S. M. Chin, S. Weigand, E. Kiskinis, S. I. Stupp, Bioactive scaffolds with enhanced supramolecular motion promote recovery from spinal cord injury. *Science* **374**, 848–856 (2021).

4. R. Cruz-Acuña, M. Quirós, S. Huang, D. Siuda, J. R. Spence, A. Nusrat, A. J. García, PEG-4MAL hydrogels for human organoid generation, culture, and in vivo delivery. *Nat. Protoc.* **13**, 2102–2119 (2018).

5. C. Redondo-Gómez, Y. Abdouni, C. R. Becer, A. Mata, Self-assembling hydrogels based on a complementary host-guest peptide amphiphile pair. *Biomacromolecules* **20**, 2276–2285 (2019).

6. J. Silva-Correia, J. M. Oliveira, S. G. Caridade, J. T. Oliveira, R. A. Sousa, J. F. Mano, R. L. Reis, Gellan gum-based hydrogels for intervertebral disc tissue-engineering applications. *J. Tissue Eng. Regen. Med.* **5**, e97–e107 (2011).

7. S. Tang, B. M. Richardson, K. S. Anseth, Dynamic covalent hydrogels as biomaterials to mimic the viscoelasticity of soft tissues. *Prog. Mater. Sci.* **120**, 100738 (2021).

8. S. Khetan, M. Guvendiren, W. R. Legant, D. M. Cohen, C. S. Chen, J. A. Burdick, Degradation-mediated cellular traction directs stem cell fate in covalently crosslinked three-dimensional hydrogels. *Nat. Mater.* **12**, 458–465 (2013).

9. I. K. Piechocka, R. G. Bacabac, M. Potters, F. C. Mackintosh, G. H. Koenderink, Structural hierarchy governs fibrin gel mechanics. *Biophys. J.* **98**, 2281–2289 (2010).

10. S. Nam, K. H. Hu, M. J. Butte, O. Chaudhuri, Strain-enhanced stress relaxation impacts nonlinear elasticity in collagen gels. *Proc. Natl. Acad. Sci. U.S.A.* **113**, 5492–5497 (2016).

11. M. Diba, S. Spaans, S. I. S. Hendrikse, M. M. C. Bastings, M. J. G. Schotman, J. F. Van Sprang, D. J. Wu, F. J. M. Hoeben, H. M. Janssen, P. Y. W. Dankers, Engineering the dynamics of cell adhesion cues in supramolecular hydrogels for facile control over cell encapsulation and behavior. *Adv. Mater.* **33**, e2008111 (2021).

12. L. Rijns, J. W. Peeters, S. I. S. Hendrikse, M. E. J. Vleugels, X. Lou, H. M. Janssen, E. W. Meijer, P. Y. W. Dankers, Importance of molecular and bulk dynamics in supramolecular hydrogels in dictating cellular spreading. *Chem. Mater.* **35**, 8203–8217 (2023).

13. L. Rijns, M. J. Hagelaars, J. J. B. Van Der Tol, S. Loerakker, C. V. C. Bouten, P. Y. W. Dankers, The importance of effective ligand concentration to direct epithelial cell polarity in dynamic hydrogels. *Adv. Mater.* **36**, 2300873 (2023).

14. G. Li, K. Huang, J. Deng, M. Guo, M. Cai, Y. Zhang, C. F. Guo, Highly conducting and stretchable double-network hydrogel for soft bioelectronics. *Adv. Mater.* **34**, e2200261 (2022).

15. M. C. Darnell, J. Y. Sun, M. Mehta, C. Johnson, P. R. Arany, Z. Suo, D. J. Mooney, Performance and biocompatibility of extremely tough alginate/polyacrylamide hydrogels. *Biomaterials* **34**, 8042–8048 (2013).

16. J. P. Gong, Y. Katsuyama, T. Kurokawa, Y. Osada, Double-network hydrogels with extremely high mechanical strength. *Adv. Mater.* **15**, 1155–1158 (2003).

17. P. Lappalainen, T. Kotila, A. Jégou, G. Romet-Lemonne, Biochemical and mechanical regulation of actin dynamics. *Nat. Rev. Mol. Cell Biol.* **23**, 836–852 (2022).

18. F. Huber, A. Boire, M. P. López, G. H. Koenderink, Cytoskeletal crosstalk: When three different personalities team up. *Curr. Opin. Cell Biol.* **32**, 39–47 (2015).

19. G. C. Na, L. J. Butz, R. J. Carroll, Mechanism of in vitro collagen fibril assembly. Kinetic and morphological studies. *J. Biol. Chem.* **261**, 12290–12299 (1986).

20. J. Lou, R. Stowers, S. Nam, Y. Xia, O. Chaudhuri, Stress relaxing hyaluronic acid-collagen hydrogels promote cell spreading, fiber remodeling, and focal adhesion formation in 3D cell culture. *Biomaterials* **154**, 213–222 (2018).

21. M. Dogterom, G. H. Koenderink, Actin-microtubule crosstalk in cell biology. *Nat. Rev. Mol. Cell Biol.* **20**, 38–54 (2019).

22. F. Burla, J. Tauber, S. Dussi, J. van der Gucht, G. H. Koenderink, Stress management in composite biopolymer networks. *Nat. Phys.* **15**, 549–553 (2019).

23. S. H. Kim, J. Turnbull, S. Guimond, Extracellular matrix and cell signalling: The dynamic cooperation of integrin, proteoglycan and growth factor receptor. *J. Endocrinol.* **209**, 139–151 (2011).

24. M. Hu, Z. Ling, X. Ren, Extracellular matrix dynamics: Tracking in biological systems and their implications. *J. Biol. Eng.* **16**, 13 (2022).

25. J. Hervy, D. J. Bicout, Dynamical decoration of stabilized-microtubules by Tau-proteins. *Sci. Rep.* **9**, 12473 (2019).

26. M. Gratuze, G. Cisbani, F. Cicchetti, E. Planel, Is Huntington's disease a tauopathy? *Brain* **139**, 1014–1025 (2016).

27. P. H. J. Kouwer, M. Koepf, V. A. A. le Sage, M. Jaspers, A. M. van Buul, Z. H. Eksteen-Akeroyd, T. Woltinge, E. Schwartz, H. J. Kitto, R. Hoogenboom, S. J. Picken, R. J. M. Nolte, E. Mendes, A. E. Rowan, Responsive biomimetic networks from polyisocyanopeptide hydrogels. *Nature* **493**, 651–655 (2013).

28. S. I. S. Hendrikse, S. P. W. Wijnands, R. P. M. Lafleur, M. J. Pouderoijen, H. M. Janssen, P. Y. W. Dankers, E. W. Meijer, Controlling and tuning the dynamic nature of supramolecular polymers in aqueous solutions. *Chem. Commun.* **53**, 2279–2282 (2017).

29. R. P. Sijbesma, F. H. Beijer, L. Brunsveld, B. J. B. Folmer, J. H. K. K. Hirschberg, R. F. M. Lange, J. K. L. Lowe, E. W. Meijer, Reversible polymers formed from self-complementary monomers using quadruple hydrogen bonding. *Science* **278**, 1601–1604 (1997).

30. R. P. M. Lafleur, S. Herziger, S. M. C. Schoenmakers, A. D. A. Keizer, J. Jahzerah, B. N. S. Thota, L. Su, P. H. H. Bomans, N. A. J. M. Sommerdijk, A. R. A. Palmans, R. Haag, H. Friedrich, C. Böttcher, E. W. Meijer, Supramolecular double helices from small C3-symmetrical molecules aggregated in water. *J. Am. Chem. Soc.* **142**, 17644–17652 (2020).

31. C. M. A. Leenders, L. Albertazzi, T. Mes, M. M. E. Koenigs, A. R. A. Palmans, E. W. Meijer, Supramolecular polymerization in water harnessing both hydrophobic effects and hydrogen bond formation. *Chem. Commun.* **49**, 1963–1965 (2013).

32. A. Van der Flier, A. Sonnenberg, Structural and functional aspects of filamins. *Biochim. Biophys. Acta* **1538**, 99–117 (2001).

33. M. Fernandez-Castano Romera, R. P. M. Lafleur, C. Guibert, I. K. Voets, C. Storm, R. P. Sijbesma, Strain stiffening hydrogels through self-assembly and covalent fixation of semi-flexible fibers. *Angew. Chem. Int. Ed. Engl.* **56**, 8771–8775 (2017).

34. N. Meechai, A. M. Jamieson, J. Blackwell, D. A. Carrino, R. Bansal, Viscoelastic properties of aggrecan aggregate solutions: Dependence on aggrecan concentration and ionic strength. *J. Rheol.* **46**, 685–707 (2002).

35. O. Esue, A. A. Carson, Y. Tseng, D. Wirtz, A direct interaction between actin and vimentin filaments mediated by the tail domain of vimentin. *J. Biol. Chem.* **281**, 30393–30399 (2006).

36. Y.-C. Lin, G. H. Koenderink, F. C. Mackintosh, D. A. Weitz, Control of non-linear elasticity in F-actin networks with microtubules. *Soft Matter* **7**, 902–906 (2011).

37. L. Albertazzi, D. Van Der Zwaag, C. M. A. Leenders, R. Fitzner, R. W. Van Der Hofstad, E. W. Meijer, Probing exchange pathways in one-dimensional aggregates with super-resolution microscopy. *Science* **344**, 491–495 (2014).

38. Z. Gong, S. E. Szczesny, S. R. Caliari, E. E. Charrier, O. Chaudhuri, X. Cao, Y. Lin, R. L. Mauck, P. A. Janmey, J. A. Burdick, V. B. Shenoy, Matching material and cellular timescales maximizes cell spreading on viscoelastic substrates. *Proc. Natl. Acad. Sci. U.S.A.* **115**, E2686–E2695 (2018).

39. A. Elosegui-Artola, I. Andreu, A. E. M. Beedle, A. Lezamiz, M. Uroz, A. J. Kosmalska, R. Oria, J. Z. Kechagia, P. Rico-Lastres, A. L. Le Roux, C. M. Shanahan, X. Trepat, D. Navajas, S. Garcia-Manyes, P. Roca-Cusachs, Force triggers YAP nuclear entry by regulating transport across nuclear pores. *Cell* **171**, 1397–1410.e14 (2017).

40. L. Rijns, M. B. Baker, P. Y. W. Dankers, Using chemistry to recreate the complexity of the extracellular matrix: Guidelines for supramolecular hydrogel–cell interactions. *J. Am. Chem. Soc.* **146**, 17539–17558 (2024).

41. M. A. Wozniak, R. Desai, P. A. Solski, C. J. Der, P. J. Keely, ROCK-generated contractility regulates breast epithelial cell differentiation in response to the physical properties of a three-dimensional collagen matrix. *J. Cell Biol.* **163**, 583–595 (2003).

42. T. Bouzid, E. Kim, B. D. Riehl, A. M. Esfahani, J. Rosenbohm, R. Yang, B. Duan, J. Y. Lim, The LINC complex, mechanotransduction, and mesenchymal stem cell function and fate. *J. Biol. Eng.* **13**, 68 (2019).

43. A. A. Khilan, N. A. Al-Maslamani, H. F. Horn, Cell stretchers and the LINC complex in mechanotransduction. *Arch. Biochem. Biophys.* **702**, 108829 (2021).

44. B. Cheng, M. Li, W. Wan, H. Guo, G. M. Genin, M. Lin, F. Xu, Predicting YAP/TAZ nuclear translocation in response to ECM mechanosensing. *Biophys. J.* **122**, 43–53 (2023).

45. C. P. Broedersz, K. E. Kasza, L. M. Jawerth, S. Münster, D. A. Weitz, F. C. MacKintosh, Measurement of nonlinear rheology of cross-linked biopolymer gels. *Soft Matter* **6**, 4120–4127 (2010).

46. J. Vandaele, B. Louis, K. Liu, R. Camacho, P. H. J. Kouwer, S. Rocha, Structural characterization of fibrous synthetic hydrogels using fluorescence microscopy. *Soft Matter* **16**, 4210–4219 (2020).

47. J. Barrasa-Fano, A. Shapeti, Á. Jorge-Peñas, M. Barzegari, J. A. Sanz-Herrera, H. Van Oosterwyck, TFMLAB: A MATLAB toolbox for 4D traction force microscopy. *SoftwareX* **15**, 100723 (2021).

48. H. Yuan, K. Liu, M. Cóndor, J. Barrasa-Fano, B. Louis, J. Vandaele, P. de Almeida, Q. Coucke, W. Chen, E. Oosterwijk, C. Xing, H. Van Oosterwyck, P. H. J. Kouwer, S. Rocha, Synthetic fibrous hydrogels as a platform to decipher cell–matrix mechanical interactions. *Proc. Natl. Acad. Sci. U.S.A.* **120**, e2216934120 (2023).

49. A. P. Thompson, H. M. Aktulga, R. Berger, D. S. Bolintineanu, W. M. Brown, P. S. Crozier, P. J. in 't Veld, A. Kohlmeyer, S. G. Moore, T. D. Nguyen, R. Shan, M. J. Stevens, J. Tranchida, C. Trott, S. J. Plimpton, LAMMPS–A flexible simulation tool for particle-based materials modeling at the atomic, meso, and continuum scales. *Comput. Phys. Commun.* **271**, 108171 (2022).

50. J. Colombo, E. Del Gado, Stress localization, stiffening, and yielding in a model colloidal gel. *J. Rheol.* **58**, 1089–1116 (2014).

51. M. Bantawa, W. A. Fontaine-Seiler, P. D. Olmsted, E. Del Gado, Microscopic interactions and emerging elasticity in model soft particulate gels. *J. Phys. Condens. Matter* **33**, 414001 (2021).

52. M. Geri, B. Keshavarz, T. Divoux, C. Clasen, D. J. Curtis, G. H. McKinley, Time-resolved mechanical spectroscopy of soft materials via optimally windowed chirps. *Phys. Rev. X* **8**, 041042 (2018).

53. M. Bouzid, B. Keshavarz, M. Geri, T. Divoux, E. Del Gado, G. H. McKinley, Computing the linear viscoelastic properties of soft gels using an optimally windowed chirp protocol. *J. Rheol.* **62**, 1037–1050 (2018).

54. A. W. Lees, S. F. Edwards, The computer study of transport processes under extreme conditions. *J. Phys. C. Solid State Phys.* **5**, 1921–1928 (1972).

55. A. P. Thompson, S. J. Plimpton, W. Mattson, General formulation of pressure and stress tensor for arbitrary many-body interaction potentials under periodic boundary conditions. *J. Chem. Phys.* **131**, 154107 (2009).

56. S. Bhattacharya, K. E. Gubbins, Fast method for computing pore size distributions of model materials. *Langmuir* **22**, 7726–7731 (2006).

57. D. Feng, J. Notbohm, A. Benjamin, S. He, M. Wang, L.-H. Ang, M. Bantawa, M. Bouzid, E. Del Gado, R. Krishnan, M. R. Pollak, Disease-causing mutation in α-actinin-4 promotes podocyte detachment through maladaptation to periodic stretch. *Proc. Natl. Acad. Sci. U.S.A.* **115**, 1517–1522 (2018).

58. E. Vereroudakis, M. Bantawa, R. P. M. Lafleur, D. Parisi, N. M. Matsumoto, J. W. Peeters, E. Del Gado, E. W. Meijer, D. Vlassopoulos, Competitive supramolecular associations mediate the viscoelasticity of binary hydrogels. *ACS Cent. Sci.* **6**, 1401–1411 (2020).

59. A. Stukowski, Visualization and analysis of atomistic simulation data with OVITO–The Open Visualization Tool. *Modelling Simul. Mater. Sci. Eng.* **18**, 015012 (2010).
